# A Recent Prevalence of Hepatitis B Virus (HBV) Genotypes and Subtypes in Asia: A Systematic Review and Meta-Analysis

**DOI:** 10.3390/healthcare11071011

**Published:** 2023-04-01

**Authors:** Kizito Eneye Bello, Tuan Nur Akmalina Mat Jusoh, Ahmad Adebayo Irekeola, Norhidayah Abu, Nur Amalin Zahirah Mohd Amin, Nazri Mustaffa, Rafidah Hanim Shueb

**Affiliations:** 1 Department of Medical Microbiology and Parasitology, School of Medical Sciences, Universiti Sains Malaysia, Health Campus, Kubang Kerian 16150, Kelantan, Malaysia; 2 Department of Microbiology, Faculty of Natural Science, Kogi State University (Prince Abubakar Audu University), Anyigba 1008, Kogi State, Nigeria; 3 Microbiology Unit, Department of Biological Sciences, College of Natural and Applied Sciences, Summit University Offa, Offa 4412, Kwara State, Nigeria; 4 Advanced Materials Research Centre (A.M.R.E.C.), Lot 34 Jalan Hi-Tech 2/3, Kulim Hi-Tech Park, Kulim 09000, Kedah, Malaysia; 5 Department of Medicine, School of Medical Sciences, Universiti Sains Malaysia, Health Campus, Kubang Kerian 16150, Kelantan, Malaysia; nazri.mustaffa@usm.my; 6 Hospital Universiti Sains Malaysia, Kubang Kerian 16150, Kelantan, Malaysia; 7 Institute for Research in Molecular Medicine (I.N.F.O.R.M.M.), Universiti Sains Malaysia, Kubang Kerian 16150, Kelantan, Malaysia

**Keywords:** hepatitis B virus, prevalence, Asia, systematic review, meta-analysis, genotype and subtype

## Abstract

Background and Aim: Despite introducing the hepatitis B virus (HBV) vaccine, the incidence of the Hepatitis B virus globally is still a major health concern. This systematic review and meta-analysis were conducted to provide detailed information on the prevalence of HBV genotypes and subtypes in circulation in Asia. Methods: A systematic search for articles describing the prevalence of HBV genotypes and subtypes in Asia was conducted following the Preferred Reporting Items for Systematic Reviews and Meta-analysis (PRISMA) guidelines. Results: Our search returned 207 eligible articles involving 49,279 genotypes and 7457 subtypes representing 28 Asian countries. A meta-analysis was performed on our eligible studies using the Random effect Model. The pooled prevalence of HBV genotypes showed that genotype C (30.9%) (95% CI, 27.5–34.5%; *I*^2^ = 97.57%; *p* < 0.001) was the most common HBV genotype in Asia, followed by genotype B (17.8%) (95% CI, 15.5–20.4%; *I*^2^ = 97.26%; *p* < 0.001) and genotype D (15.4%) (95% CI, 11.8–19.8%). Vietnam had the highest prevalence of genotype B, Lebanon had the highest prevalence of genotypes C, and Jordan had the highest prevalence of genotype D. There was variation in genotypic prevalence with respect to the target genes for HBV genotyping. Reverse dot blot hybridization had the highest estimate of genotypes B and C. HBV subtype C2 (40.0%) (95% CI, 33.3–47.0) is the most prevalent HBV subtype. Conclusion: Evidence from this study reveals that HBV genotypes C and B are the most dominant HBV genotypes in Asia, and HBV subtype C2 is more endemic in Asia.

## 1. Background

Hepatitis B virus (HBV) infection is still of immense health concern despite the introduction of the HBV vaccine in the late 1990s. Approximately two billion cases have been reported globally from the discovery of the disease to the early 21st century, with over 400 million such cohorts progressing into chronic HBV carriers [1]. Chronic HBV infection has been implicated as a significant cause of many liver-related medical complications, such as hepatocellular carcinoma, cirrhosis, and liver failure [2,3,4]. HBV is a prominent member of Hepadnaviridae, and its genome consists of circular D.N.A. with a length of 3.2 kb [5]. The genome contains an overlapping region that encodes four distinct genes (S, C, P, and X). The S gene solely codes for a surface protein in the HBV envelope within its long open reading frame [6]. The presence of start codons splices the gene into small, medium, and large polypeptides, which are usually used either as a single target gene or in combination with other HBV genes to detect HBV DNA [5]. The HBV core antigen is traditionally coded by the C gene, while the X gene codes for the X protein [7]. The P gene codes for the Polymerase protein, an integral constituent of the reverse transcription process in the HBV replication. The replication cycle of HBV lacks a proofreading trait and, thus, usually gives rise to a high genomic variable progeny [5].

Due to significant genetic diversity, HBV is categorized into ten genotypes (A-J) with a 7.5 percent intergroup variance [8]. Apart from E and G, all genotypes are classified into 25 sub-genotypes, each with a 4 percent variability in amino acids [9,10]. HBV genotypes are distributed differently depending on geographical location: HBV-B, HBV-C, and HBV-E are most common in Oceania and Eastern Asia, whereas HBV-E is most common in Central and Western Africa. HBV-F and HBV h are only found in Alaska and Latin America. In contrast, HBV-D is a global pandemic. In Australia, Europe, Indonesia, North Africa, and Western Asia, HBV-D1 is the most prevalent virus, while HBV-D2 is found in Albania, Japan, Malaysia, North-Eastern Europe, Russia, and the United Kingdom [11,12,13,14,15].

The disease progression and natural course are not the same in different HBV genotypes. The latter could make treating HBV very challenging as the efficiency of known therapeutic drugs is rendered ineffective against certain genotypes and new genotypic variants [8,9]. As such, there is a great demand for genotypic information and inquiry in the case of HBV-affected people globally [15]. There is a good number of reports on the molecular epidemiology of HBV in Asia. However, the general pooled prevalence of HBV genotypes in Asia is yet to be reported, which necessitates this study. This systematic review and meta-analysis were designed to determine the pool prevalence of Hepatitis B genotypes and subtypes in Asia and to explore and compare the prevalence of HBV genotypes across countries, the genomic region of target for HBV detection, and the empirical methods of genotyping within Asia in the last two decades.

## 2. Methods

### 2.1. Search Strategy

Before synthesizing this review, a preliminary key term search was first carried out on two review databases (PROSPERO and DARE) to avoid redundancy or repetition of already published information or ongoing projects. This review adheres to Preferred Reporting Items for Systematic Reviews and Metanalysis guidelines for its synthesis [10]. We searched four international electronic databases (PubMed, Google Scholar, Scopus, and Science Direct) for articles reporting HBV genotypes or/and subtypes across Asia with the key search terms “genotype”, “Hepatitis B”, and “Subtypes” and a list of Asian countries as combinations of search strategies. We used synonymous keyword search, abbreviations, and Boolean operators in the search option where necessary. The search strategy for the four electronic databases is provided in Supplementary File S1. We carried out a final search on 23rd December 2021.

All references of recovered search results from the search strategies across the different databases were imported into Mendeley desktop reference manager software to remove duplicates and further screening. A detailed search strategy is provided in File S1 in the Supplementary material.

### 2.2. Eligibility and Data Extraction

Cross-sectional studies, prospective cohorts, and retrospective cohorts were all included in this review. Original studies with HBV genotyping or subtypes were included if the samples or data were collected in at least one of the Asian countries. We excluded studies that were (1) reviews, (2) editorials, (3) without a well-defined sample source and origins, (4) contained redundant data or duplicate, (5) with a serological and biochemical report only, or (6) that we could not recover its full text. Four authors (B.K.E, A.A.I, T.N.A.M.J., and N.A.Z.M.A.) completed an independent title, abstract screening, and full-text review based on the inclusion criteria. Disputes were handled by consensus among the authors or by a fifth independent author’s decision (R.H.S.). The titles, abstracts, and full texts of the qualified studies were screened under the appropriate headings (title, abstract and full text). Relevant data such as the name of authors, year of publication, country of study, region of the target, method of genotyping, the total number of genotypes and subtypes, and the different genotypes and subtypes were extracted into a structured proforma. Six authors extracted the data (B.K.E., A.A.I., N.A., N.A.Z.M.A., T.N.A.M.J. and R.H.S.).

### 2.3. Statistical Analysis and Quality Assessment

A single-arm random-effects model was used in all analyses to determine the pooled prevalence of various HBV genotypes/subtypes. The latter led us to use the meta-analysis method of DerSimonian and Laird, which is embedded in the OpenMeta and Comprehensive Meta-analysis Software [11]. A funnel plot was used to examine the publication bias. Cochran’s Q test assessed the heterogeneities of subgroups estimates.

Statistically, the heterogeneity index was measured via the Cochran Q test and *I*^2^ values, where an *I*^2^ value of 25, 50, and 75% represented low, moderate, and high degrees of heterogeneity, respectively [12,13]. A subgroup analysis was also conducted to evaluate the prevalence of HBV genotypes among nations, genotypic detection methods, and targeted gene regions. Subgroup analysis was performed using OpenMeta Analyst software version 10.10 [14]. Descriptive statistics were used to summarize data about the genotype and subtypes of HBV within Asia. A *p*-value of <0.001 was statistically significant for all tests. A protocol was developed for this review with PROSPERO ID no. CRD42022321763.

The overall quality of the method of the included articles for this review was assessed using the Joanna Briggs Institute (J.B.I.) critical appraisal checklist for prevalence data [15] (Supplementary File S3). To obtain a total quality score that ranged from 0 to 18, a score of “2” for “yes” and “0” for “no” were assigned. Studies having a quality score of 14–18 were considered adequate. Three authors evaluated the studies (B.K.E., A.A.I. and N.M.). Supplementary Table S4 contains a quality assessment of the 207 studies included.

## 3. Results

### 3.1. Search Results and Eligible Studies

**Figure 1:**
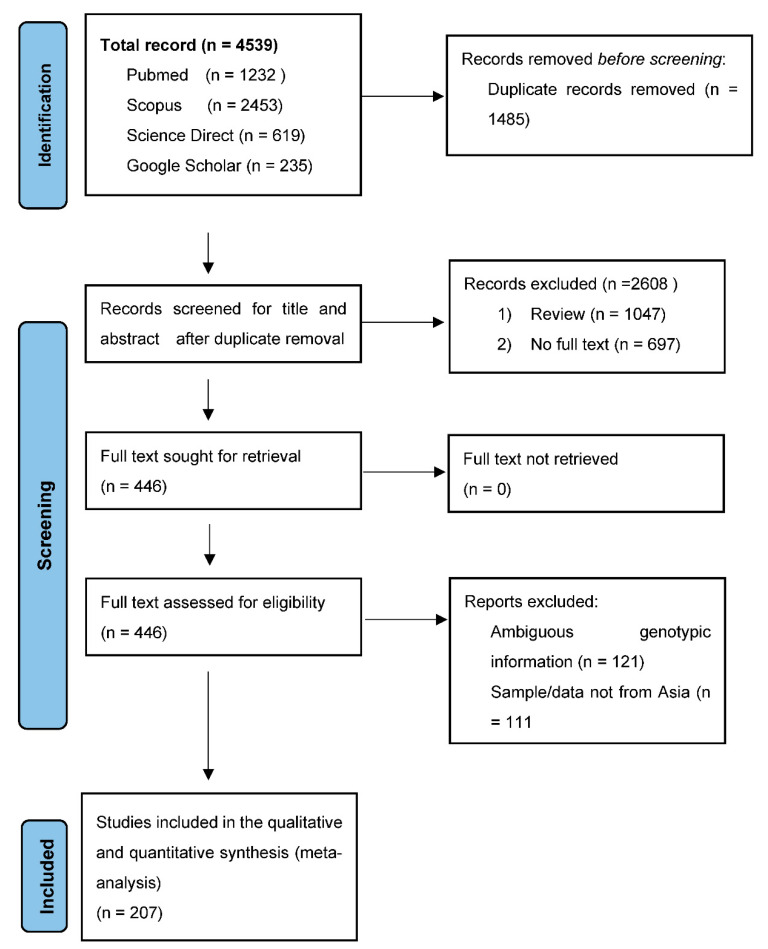
Summary of the article selection process.

An initial 4539 abstracts were recovered from five international electronic databases using our search approach. All duplicates were removed, and 2608 articles were discarded based on their titles and abstracts. A total of 446 papers were eligible for full-text evaluation, but 232 were rejected because they did not meet our inclusion criteria or had a low J.B.I. assessment score. Figure 1 shows a full overview of the selection procedure. This systematic literature review and meta-analysis included 207 publications of 49,279 HBV genotypes.

### 3.2. Characteristics of the Eligible Studies

The included studies were conducted across the 48 countries representing the Asian continent. Based on the inclusion criteria, our study recovered 207 [4,9,12,13,14,15,16,17,18,19,20,21,22,23,24,25,26,27,28,29,30,31,32,33,34,35,36,37,38,39,40,41,42,43,44,45,46,47,48,49,50,51,52,53,54,55,56,57,58,59,60,61,62,63,64,65,66,67,68,69,70,71,72,73,74,75,76,77,78,79,80,81,82,83,84,85,86,87,88,89,90,91,92,93,94,95,96,97,98,99,100,101,102,103,104,105,106,107,108,109,110,111,112,113,114,115,116,117,118,119,120,121,122,123,124,125,126,127,128,129,130,131,132,133,134,135,136,137,138,139,140,141,142,143,144,145,146,147,148,149,150,151,152,153,154,155,156,157,158,159,160,161,162,163,164,165,166,167,168,169,170,171,172,173,174,175,176,177,178,179,180,181,182,183,184,185,186,187,188,189,190,191,192,193,194,195,196,197,198,199,200,201,202,203,204,205,206,207,208,209,210,211,212,213,214,215,216] articles in 27 Asian countries, with China contributing the most significant number of studies (*n* = 68). A total of 49,279 HBV genotypes were reported across 207 studies ranging from 6817 HBV genotypes (China) to 2 HBV genotypes (Iran) (Supplementary Table S5).

While HBV genotype information was available for all included studies, only 29 studies provided data on HBV subtypes (Supplementary File S6). A total of 7457 HBV subtypes were reported, consisting of 27 different HBV subtypes, with HBV subtype C2 having the highest prevalence (40.0%) (Table 1). The reported genotypes and subtypes varied across countries.

A summary of included articles is provided in Supplementary Table S5. The quality assessment shows that most of the included articles are of high methodological quality (Supplementary Table S4).

### 3.3. Prevalence of HBV Genotype in Asia

The pooled prevalence of HBV genotypes was mostly heterogenous in distribution with the exception of genotypes E, G, H, and I, whose heterogeneity indices were less than 50%. Genotype A was estimated as 2.9% (95% CI, 2.1–4.1%, *I*^2^= 86.74%, *p* < 0.001), genotype B as 17.5% (95% CI, 14.0–21.7%; *I*^2^ = 96.83%; *p* < 0.001), genotype C as 27.8% (95% CI, 23.1–33.1%; *I*^2^= 97.17%; *p* < 0.001) (S7, Supplementary File), genotype D as 22.7% (95% CI, 15.6–31.8%; *I*^2^ = 97.30%; *p* < 0.001), genotype E as 0.6% (95% CI, 0.4–0.9%; *I*^2^ = 32.02%; *p* = 0.004), genotype F as 0.6% (95% CI, 0.3–1.2%; *I*^2^ = 82.64%; *p* < 0.001), genotype G as 0.6% (95% CI, 0.4–0.8%; *I*^2^ = 0.00%; *p* = 0.527), genotype H as 0.6% (95% CI, 0.4–0.8%; *I*^2^ = 0.00%, *p* = 0.538), and genotype I as 0.7% (95% CI, 0.5–1.0; *I*^2^ = 0.00%, *p* = 0.215). Corresponding forest plots are shown in file S.F.A 1, which is available in the Supplementary Files.

**Figure 2:**
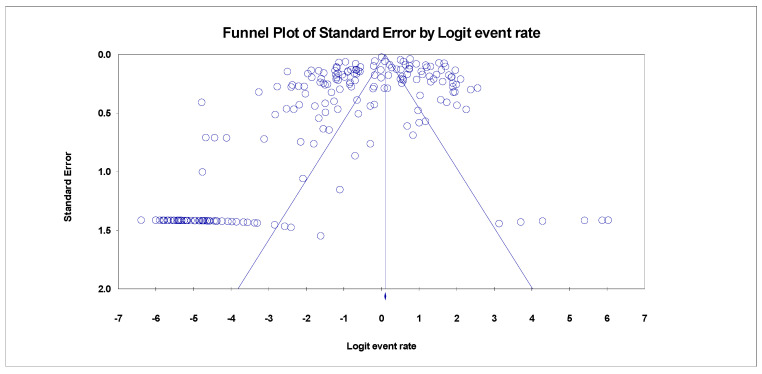
A funnel plot showing no publication bias for HBV genotype C in Asia (Egger’s *p* = 0.00001).

The funnel plot for HBV genotype C in Asia showed no publication bias (Figure 2). However, there was a publication bias in studies reporting other HBV genotypes (Supplementary File FP1 to FP3).

**Figure 3:**
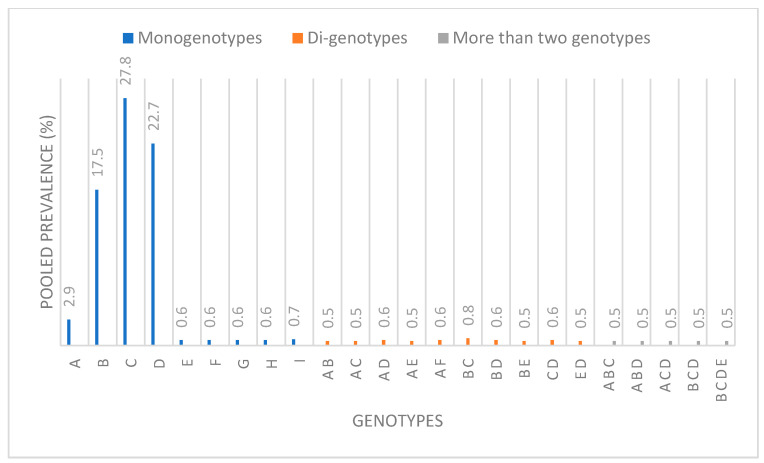
A chart showing the pooled prevalence of HBV genotypes in Asia.

A summary of the genotypic pooled prevalence of HBV in Asia is presented in Figure 3.

### 3.4. Distribution of HBV Subtypes in Asia

In this study, 7457 HBV subtypes were reported across the 29 studies and ranged from 5637 HBV subtypes (in China) to 10 HBV subtypes (in India). Data on HBV subtypes were available in studies from Bangladesh (*n* = 1), Cambodia (*n* = 1), China (*n* = 11), India (*n* = 1), Indonesia (*n* = 2), Iran (*n* = 2), Japan (*n* = 1), Korea (*n* = 1), Malaysia (*n* = 1), Thailand (*n* = 1), Turkey (*n* = 5), and Vietnam (*n* = 2) (Supplementary file S2)

The most prevalent HBV subtypes In Asia were HBV subtype C2, with a pool prevalence of 40% (95% CI, 33.3–47.0%; *I*^2^ = 85.39%), followed by HBV subtype C1 at 33.5% (95% CI, 27.0–40.7%; *I*^2^ = 81.69%) and HBV subtype B3 at 29.9% (95% CI, 22.5–38.5; *I*^2^ = 70.27%). The prevalence of other reported subtypes is reported in Table 1.

### 3.5. Subgroup Analysis for HBV Mono Genotypes

To examine HBV genotype distribution among Asian countries, the gene of targets, and HBV genotyping methods, as well as to determine the possible source of heterogeneity among the studies, a subgroup analysis was carried out.

The distribution of HBV genotypes among nations indicated varying degrees of heterogeneity in the studies (Table 2). Overall, diverse estimates were recorded for the different genotypes. For genotype A, Nepal had the highest estimate (21%). In comparison, Myanmar had the lowest (0.3%) with one study; there was a high heterogeneity index of genotype A from studies from Pakistan, China, Indonesia, Japan, and India (*I*^2^ = >85%) (Table 2). Studies from Vietnam (*I*^2^ = 0.0%), Thailand (*I*^2^ = 0.00%), and Cambodia (*I*^2^ = 0.77%) showed low heterogeneity.

For genotype B, Vietnam had the highest estimate (76.8%), while Serbia (0.3%) and Oman (0.3%) had the lowest (Table 2). Studies from China had the highest heterogeneity (*I*^2^ = 98.39%), while studies from Uzbekistan, Turkey, Vietnam, and Cambodia had the lowest heterogeneity (*I*^2^ = 0.0%) (Table 2).

Although in a single study, the highest estimate (95.8%) for genotype C was from Lebanon. Meanwhile, Serbia had the lowest estimates (0.3%). Studies from China had the highest heterogeneity (*I*^2^ = 98.34%). Iran, Cambodia, Uzbekistan, Turkey, and Malaysia had the lowest heterogeneity (*I*^2^ = 0.0%) compared to other countries’ studies (Table 2).

For genotype D, studies from Jordan had the highest estimate (99.3%), while Korea (0.7%) had the lowest, with only two studies (Table 2). Studies from Japan had the highest heterogeneity (*I*^2^ = 97.79%), while studies from Vietnam and Cambodia had the lowest heterogeneity (*I*^2^ = 0.0%).

The highest estimate (8.2%) for genotype E was from Malaysia, although with only two studies. Meanwhile, studies from Japan, China, Serbia, Myanmar, and Afghanistan had the lowest estimates (0.3% each). Studies from Malaysia had the highest heterogeneity (*I*^2^ = 89.72%) (Table 2).

Genotype F had the highest estimates from Iraq (31.2%), and genotypes G, H, and I had the highest estimates (4.2% each) from Lebanon. In comparison, Japan had the lowest (0.2%) across genotypes G and I; for genotype F, a high heterogeneity index was observed in studies from Taiwan (*I*^2^ = 81.25%); genotype G had the highest heterogeneity in studies from Taiwan (*I*^2^ = 63.64%) and Malaysia (*I*^2^ = 35.48%), respectively. Genotype I had the highest level of heterogeneity observed in studies from Malaysia (*I*^2^ = 35.48%) (Table 2). The corresponding forest plots are provided in file S8 in the Supplementary Files.

The subgroup meta-analysis of HBV genotypes based on the target gene reveals a diverse study heterogeneity, ranging from low to high heterogeneity. For genotype A, the C target gene had the highest estimate (14.8%), while studies that targeted a combination of P and C genes had the lowest estimate (0.3%) (Supplementary Table S9). The highest heterogeneity within the subgroup for genotype A was observed with studies targeting the P and S genes (93.33%). The lowest heterogeneity was observed with studies reporting X gene (0.0%) in HBV genotype A detection. The P, S, and C genes had the highest estimate (28.9%) for genotype B, while P and C had the lowest estimate (0.3%). For genotype C, studies reporting the X gene as the sole gene of the target had the largest estimate (82.6%), while studies that reported P and C had the lowest estimate (0.3%) (Supplementary Table S9).

For genotype D, studies that reported P and C as the sole gene of the target in HBV detection and genotyping had the highest estimate (99.7%). In comparison, studies that reported the X gene as their target gene had the lowest estimate (0.4%). The highest level of heterogeneity for HBV genotype D was observed in studies reporting S and C as their sole target gene (98.29%). In contrast, the X gene target had the lowest in this category (Supplementary Table S9).

Subgroup analysis for genotypes B and C reveals a high level of heterogeneity for the genes of targets within both genotypes (>85%). In contrast, genotypes E, G, H, and I had a low level of heterogeneity (<50%). For genotypes E, F, H, and I, the C-targeted gene had the highest estimates across the various genotypes (0.9%) (Supplementary Table S9). The corresponding forest plots are provided in file S.F 1 in the Supplementary Files.

HBV genotype subgroup analysis based on the genotyping method reveals sparsely heterogenous studies across the varying genotypes. For genotype A, studies that reported using the RFLP method had the highest estimate (4.5%). In comparison, studies that reported using the R.D.B. hybridization method had the least estimate (0.9%). Heterogeneity was high (*I*^2^ = >90%) for studies in this genotypic category (genotype A) (Supplementary Table S10).

For genotype B, R.D.B. hybridization methods had the highest estimate (50.0%), while studies that reported RFLP as their only form of HBV detection had the least estimate (8.9%). All studies in this genotypic subgrouping category (genotype B) were highly heterogenous (*I*^2^ = >90%). R.D.B. hybridization also had the highest estimate (39.8%) for genotype C, while multiplex PCR had the lowest estimate (23.6%). All studies within this genotype subgrouping were highly heterogenous (*I*^2^ = >95%) (Supplementary Table S10).

For genotype D, RFLP had the highest estimate (36.5%). In comparison, R.D.B. hybridization had the lowest estimate (0.6%). The highest level of heterogeneity for genotype D subgrouping was observed in studies reporting sequencing as their method of HBV genotype detection (*I*^2^ = 97.54%). In comparison, the lowest level of heterogeneity was recorded in studies reporting R.D.B. hybridization as their method of HBV genotyping (35.46%) (Supplementary Table S10).

Other than the multiplex PCR method, which had a moderate heterogeneity (*I*^2^ =67.7%), a low level of heterogeneity was observed (*I*^2^<15%) in the genotype E subgroup

RFLP had the highest estimate (0.8%) for genotype F, while R.D.B. hybridization had the lowest estimate (0.3%). Only multiplex PCR and RFLP were the only methods of HBV detection that had a high level of heterogeneity (*I*^2^ = >75%) (Supplementary Table S10).

For genotype G, sequencing and RFLP had the highest estimate (0.6%), while the R.D.B. hybridization method had the least estimate (0.3%). All studies in this category were lowly heterogeneous.

Genotypes H and I had their highest estimates in studies reporting sequencing as their method of HBV genotyping, with an estimate of 0.6% and 0.8%, respectively (Supplementary Table S10).

The corresponding forest plots are provided in file S.F. 2 in the Supplementary Files.

## 4. Discussion

The molecular prevalence and genotypic diversity of HBV globally have been a health burden for several years, even after introducing the HBV vaccine. While there are effective vaccines and established antiviral drug therapies against HBV, the toll of HBV infection is still on the rise for the past decade as vaccine escape mutant HBV incidence is common in Asia [81]. Treatment and management of HBV vary across its diverse and ever-emerging genotypes [24]; therefore, understanding the genotypic distribution of HBV genotypes in Asia is essential for the effective treatment and management of HBV infections within Asia and the entire world. Good genotypic knowledge of HBV within Asia will help develop effective policies, programs, and interventions to prevent and manage the disease. The findings in this review are based on results gathered from a systematic review and meta-analysis of published studies on the molecular epidemiology of HBV genotypes within Asia and related subject themes and titles. This review considered the genotypes of HBV in circulation among the member countries in Asia, the method of genotyping, and the gene target for HBV detection to obtain a comprehensive overview of the molecular prevalence of HBV in Asia. This meta-analysis was carried out on 207 articles, and the results indicated that the random effect model choice was not by chance. The genotyping method, gene obtained, and country of screening showed high heterogeneity.

In this study, twenty-eight countries presented data about the HBV genotype that met our inclusion criteria. Most of the studies were from China, probably due to the heavy HBV burden in the world [171].

HBV genotype C (30.9%) was the most dominant genotype in Asia from our study, followed by genotype B (17.8%). This finding contradicts the report of Velkov et al. (2018) [210], which reported that genotype B was the most dominant HBV genotype in Asia. The variation in the prevalence of HBV genotypes could be due to the different datasets and the variations in geographical locations. Our current finding presents the need for a cautious presentation and interpretations of results conducted globally. It is noteworthy that HBV genotype C is the most dominant HBV genotype in Asia, followed by genotypes B and D (Table 2). The combination of the three genotypes accounts for two-thirds of the entire HBV genotypes reported within Asia, making them the major genotypes in circulation within the Asian continent. The findings of this systematic review and meta-analysis support the report of Rajoriya et al. (2017), who reported that HBV genotype B and genotype C was predominant in Asia and Oceania [211].

We found out that the country with the highest prevalence for HBV genotype C was Lebanon (95.8%), and HBV genotype B was notably highest in Vietnam (76.8%). The latter is probably because of the high prevalence of Hepatitis B within these countries [212]. The pool prevalence of genotype D was highest in Jordon (99.3%). The probable reason for the high prevalence of genotype D in Jordan could be its geographical proximity to Africa, as genotype D is more prevalent in North Africa [213]. Genotype A was more prevalent in Nepal (21%) and Afghanistan (20.5%).

The latter could probably be due to the heavy presence of European and American immigrants in the region due to the security crisis over the past few decades. This report supports the findings of Rajoriya et al. (2017), who reported that genotype A was more dominant in America and Europe. Our study reveals that genotypes E, F, G, H, and I pool prevalence in Asia was less than 1%. The possible reason for the low prevalence could be due to the route of transmission, as most of these genotypes are transmitted horizontally and through immigrants [211,214,215].

The methodological variability of the review was assessed, and the gene of target and method of genotyping was highly heterogenous within groups across the various genotypes. The most common target gene for HBV detection was the S gene, which was reported in 135 studies accounting for more than 60% of the total study included in this systematic review and meta-analysis. The global preference of the S gene over other HBV genes is unclear, but it could be attributed to the high conservation of the pairwise sequence that makes up the S gene. The findings of this study support the reports of Tersa et al. (2014), who reported that the HBV S gene has a lower mutation index than the HBV P gene [6]. Although the S gene is the major targeted gene for HBV detection in Asia, it does not account for the major genotypes in circulation within Asia. The X gene has the highest estimate for genotype C detection (82.6%). The combination of P, S, and C target genes had the highest estimate for genotype B (28.9%). In contrast, genes P and C had the highest estimate for genotype D (99.7%). The latter is probably due to the number of studies and samples, as some studies, such as the study reporting the X gene, can be very few.

Sequencing was our study’s most prominent method of HBV genotyping, and it accounts for over 60% of the studies included in this review. This is probably because of the precision and efficiency of the method. The findings of this study support the report of Mikael et al. (2017), who independently reported that sequencing is a more efficient method of detecting HBV and its associated mutations [216].

Sixteen recombinant genotypes were reported to be in circulation in this review. The most dominant recombinant HBV genotype was genotype B+C (0.8%). The latter is probably due to the high prevalence of HBV genotypes B and C in Asia. This finding collaborates with an earlier report by Peter and Sofie (2005), who reported that genotype B+C is predominant in Asia [217]. The presence of viral hepatitis cirrhosis in Asian patients increases the propensity and generation of HBV recombinant [218]. Since several cases of recombinant genotypes have been reported across 27 Asian countries in this study (Supplementary File S2), for optimal treatment outcomes, such infected patients will likely require more than the normal combinations of current HBV medications. Given the high expense of therapy, the discovery of a ‘recombinant genotype’ in this study reveals a new hurdle that should be considered in creating more effective drugs and drug combinations.

Even though the prevalence of HBV subtypes appears to differ in this study, we found 27 distinct subtypes (Table 1), showing that the virus subtypes in the continent are quite diverse. Subtype C2 (40.0%) is the most common subtype identified in this review. The predominance of subtype C2 complements our findings of genotype C being Asia’s most prevalent HBV genotype. This study is consistent with the report of Choong-Hwan et al. (2009), who reported that HBV subtype C2 is dominant among Chronic HBV patients in Korea [219].

## 5. Strengths and Limitations

This study is the first meta-analysis of the prevalence of HBV genotypes and subtypes in Asia to the best of our knowledge. Its strength includes the large sample size, the rigorous search of published data with precise inclusion criteria and thoroughly analyzed data, and being the first to report mixed HBV genotypic pool prevalence in Asia. However, this study has some limitations. The study’s limitation is the lack of sufficient studies in some of the included countries in Asia; as some countries have just one study that met our inclusion criteria, this could cause an under-representation of cohorts from such countries, thereby giving underestimation or misrepresentation of genotypes and subtypes. Another concern is the variability in the targeted genes for HBV detection and the method of HBV genotyping. We prioritized peer-reviewed articles from four international databases to limit the possibility of unreliable genotype data; these could have made us miss out on some relevant studies and data that are not yet published. Finally, we could not recover genotyping data from 20 countries in Asia because studies from such countries did not meet our inclusion criteria or were not available, which throws more questions on the exact status of HBV genotypes in Asia. This review provides an up-to-date comprehensive report on the prevalence of HBV genotypes and subtypes in Asia despite the above limitations.

## 6. Conclusions

This study is the first to analyze HBV genotypes and subtypes in Asia. Genotype C has the highest pool prevalence at 30.9% (95% CI; 27.5–34.5%, *I*^2^ = 97.57%; *p*< 0.001), and subtype C2 had the highest pooled prevalence at 40.0% (95% CI; 33.3–47.0%, *I*^2^ = 84.39%; *p* < 0.001). The discoveries made in this study may assist government and non-governmental agencies develop policies to tackle the Hepatitis B disease and control its spread globally. This review may also help physicians and health caregivers choose appropriate Hepatitis B antiviral therapy to treat HBV infection in Asia as a predictor of the disease’s treatment choice and preferences.

## Figures and Tables

**Table 1:** Prevalence of HBV subtypes in Asia.

Subtypes	Pooled Prevalence (%)	95% CI
A1	26.7	20.9–33.6
A5	25.6	20.1–32.1
B1	27.6	21.1–35.3
B2	29.5	24.7–34.7
B3	29.9	22.5–38.5
B4	28.6	21.9–36.4
B5	28.1	21.4–35.9
B7	26.3	20.3–33.3
B8	25.8	20.1–32.5
C1	33.5	27.0–40.7
C2	40.0	33.3–47.0
C3	27.0	20.6–34.6
C4	26.7	20.4–34.0
C5	27.9	21.4–35.4
C6	26.1	20.1–33.2
C7	26.0	20.2–32.8
C8	26.4	20.4–33.4
C9	28.2	21.4–36.2
C10	26.1	20.1–33.1
C17	26.1	20.1–33.1
D1	32.5	24.7–41.4
D2	33.1	25.3–41.9
D3	26.0	20.2–32.9
D4	26.0	20.1–33.0
D5	26.1	20.2–33.0
D6	26.0	20.2–32.9
D-DEL	26.0	20.2–32.7

**Table 2:** Subgroup analysis for comparison of prevalence of HBV genotypes across Asian countries.

Country	Number of Studies	Prevalence (%)	95% CI	*I*^2^ (%)	Q	Heterogeneity Test	
						D.F.	*p*
Genotype A							
Iran	20	1.5	0.6–3.6	59.41	46.80	19	<0.001
Uzbekistan	2	7.1	1.9–23.2	78.43	4.64	1	0.031
Pakistan	12	12.1	7.8–18.3	92.23	141.56	11	<0.001
Indonesia	8	2.7	0.4–18.0	92.81	97.42	7	<0.001
Tajikistan	1	5.9	1.9–16.7	-	-	-	-
Japan	19	7.6	4.6–12.3	96.38	497.52	18	<0.001
Bangladesh	4	19.1	9.2–35.4	33.5	4.51	3	0.211
India	21	16.0	10.8–23.2	92.61	270.55	20	<0.001
Turkey	15	2.0	0.9–4.2	55.59	31.53	14	0.005
Vietnam	4	0.8	0.2–3.1	0.00	0.922	3	0.820
Thailand	8	0.9	0.4–2.0	0.00	2.20	7	0.948
China	68	0.5	0.3–0.9	87.15	521.24	67	<0.001
Azerbaijan	1	5.8	2.6–12.4	-	-	-	-
Jordan	1	0.7	0.0–10.3	-	-	-	-
Malaysia	2	2.1	0.2–20.2	35.48	1.55	1	0.213
Korea	2	0.7	0.1–6.0	18.66	1.23	1	0.268
Serbia	1	14.2	9.6–20.5	-	-	-	-
Myanmar	1	0.3	0.0–5.0	-	-	-	-
Taiwan	6	1.4	0.3–6.4	84.44	32.13	5	<0.001
Sri Lanka	1	8.0	2.0–26.9	-	-	-	-
Nepal	3	21.0	8.6–43.1	85.03	13.36	2	0.001
Iraq	1	10.8	7.0–16.1	-	-	-	-
Oman	1	18.8	13.6–25.4	-	-	-	-
Afghanistan	1	20.5	15.0–27.4	-	-	-	-
Cambodia	2	2.9	0.4–17.9	0.00	0.084	1	0.772
Yemen	1	15.4	5.9–34.5	-	-	-	-
Lebanon	1	4.2	0.3–42.5	-	-	-	-
Overall	207	3.1	2.5–3.8	91.79	2507.63	206	<0.001
Genotype B							
Iran	20	2.1	0.7–5.9	80.52	97.55	19	<0.001
Uzbekistan	2	0.6	0.1–4.3	0.00	0.134	1	0.715
Pakistan	12	2.1	0.8–5.2	92.08	138.93	11	<0.001
Indonesia	8	56.4	33.9–76.5	95.05	141.54	7	<0.001
Tajikistan	1	1.0	0.1–13.6	-	-	-	-
Japan	19	13.5	9.7–18.4	96.4	500.07	18	<0.001
Bangladesh	4	8.1	2.0–27.5	45.59	5.51	3	0.138
India	21	1.1	0.4–3.0	80.16	100.80	20	<0.001
Turkey	15	0.8	0.4–1.5	0.00	2.55	14	1.000
Vietnam	4	76.8	71.7–81.1	0.00	2.64	3	0.450
Thailand	8	12.5	8.2–18.7	71.24	24.34	7	<0.001
China	68	36.9	31.7–42.3	98.39	4159.52	67	<0.001
Azerbaijan	1	0.5	0.00–7.2	-	-	-	-
Jordan	1	0.7	0.00–10.3	-	-	-	-
Malaysia	2	36.3	2.1–93.7	80.75	5.19	1	0.023
Korea	2	5.8	0.00–96.5	94.34	17.68	1	<0.001
Serbia	1	0.3	0.00–4.7	-	-	-	-
Myanmar	1	1.3	0.3–5.1	-	-	-	-
Taiwan	6	61.5	43.9–76.6	96.34	136.57	5	<0.001
Sri Lanka	1	36.0	19.9–56.0	-	-	-	-
Nepal	3	3.3	1.3–8.2	1.69	2.03	2	0.362
Iraq	1	31.2	24.9–38.2	-	-	-	-
Oman	1	0.3	0.00–4.5	-	-	-	-
Afghanistan	1	7.5	4.3–12.7	-	-	-	-
Cambodia	2	29.5	16.6–46.7	0.00	0.137	1	0.711
Yemen	1	1.9	0.1–23.6	-	-	-	-
Lebanon	1	4.2	0.3–42.5	-	-	-	-
Overall	207	17.8	15.5–20.4	97.26	7524.23	206	<0.001
Genotype C							
Iran	20	1.2	0.6–2.1	0.00	11.49	19	0.906
Uzbekistan	2	0.6	0.1–4.3	0.00	0.134	1	0.715
Pakistan	12	4.5	2.1–9.6	94.26	191.75	11	<0.001
Indonesia	8	24.1	11.5–43.6	91.91	86.52	7	<0.001
Tajikistan	1	1.0	0.1–13.6	-	-	-	-
Japan	19	71.1	64.4–76.9	96.73	551.20	18	<0.001
Bangladesh	4	31.4	16.8–51.0	53.05	6.39	3	0.094
India	21	10.4	6.7–15.6	88.19	169.41	20	<0.001
Turkey	15	0.9	0.5–1.80	0.00	3.13	14	0.999
Vietnam	4	21.8	16.7–27.9	23.84	3.94	3	0.268
Thailand	8	85.2	78.8–89.9	70.57	23.78	7	0.001
China	68	51.0	45.5–56.5	98.34	4033.91	67	<0.001
Azerbaijan	1	0.5	0.00–7.2	-	-	-	-
Jordan	1	0.7	0.00–1.03	-	-	-	-
Malaysia	2	30.7	22.0–41.1	0.00	0.021	1	0.884
Korea	2	94.2	3.5–100.0	94.34	17.68	1	<0.001
Serbia	1	0.3	0.00–4.7	-	-	-	-
Myanmar	1	66.7	58.8–73.7	-	-	-	-
Taiwan	6	21.1	14.0–30.6	90.13	50.68	5	<0.001
Sri Lanka	1	16.0	6.1–35.7	-	-	-	-
Nepal	3	6.5	3.3–12.3	8.42	2.18	2	0.331
Iraq	1	8.6	5.3–13.6	-	-	-	-
Oman	1	1.2	0.3–4.6	-	-	-	-
Afghanistan	1	34.2	27.3–41.8	-	-	-	-
Cambodia	2	70.5	53.3–83.4	0.00	0.137	1	0.711
Yemen	1	1.9	0.1–23.6	-	-	-	-
Lebanon	1	95.8	57.5–99.7	-	-	-	-
Overall	207	30.9	27.5–34.5	97.57	8476.52	206	<0.001
Genotype D							
Iran	20	97.4	92.7–99.1	80.5	97.43	19	<0.001
Uzbekistan	2	92.9	76.8–98.1	78.43	4.64	1	0.031
Pakistan	12	63.3	48.0–76.4	97.67	472.33	11	<0.001
Indonesia	8	2.0	0.5–7.3	72.41	25.37	7	<0.001
Tajikistan	1	94.1	83.3–98.1	-	-	-	-
Japan	19	1.0	0.3–3.8	97.76	804.30	18	<0.001
Bangladesh	4	39.0	18.0–65.2	73.31	11.24	3	0.010
India	21	68.2	57.8–77.1	94.57	368.46	20	<0.001
Turkey	15	97.4	91.7–99.2	81.59	76.03	14	<0.001
Vietnam	4	0.8	0.2–3.1	0.00	0.922	3	0.820
Thailand	8	1.6	0.8–3.3	6.48	7.49	7	0.380
China	68	1.3	0.8–1.9	94.24	1163.37	67	<0.001
Azerbaijan	1	93.2	86.4–96.7	-	-	-	-
Jordan	1	99.3	89.7–100.0	-	-	-	-
Malaysia	2	3.8	0.1–6.0	73.73	3.81	1	0.051
Korea	2	0.7	0.1–6.0	18.66	1.23	1	0.268
Serbia	1	85.8	79.5–90.4	-	-	-	-
Myanmar	1	32.0	25.1–39.8	-	-	-	-
Taiwan	6	1.7	0.4–7.3	88.51	43.52	5	<0.001
Sri Lanka	1	12.0	3.9–31.3	-	-	-	-
Nepal	3	66.3	55.9–75.3	45.08	3.64	2	0.162
Iraq	1	12.9	8.8–18.5	-	-	-	-
Oman	1	76.5	69.5–82.2	-	-	-	-
Afghanistan	1	37.9	30.7–45.6	-	-	-	-
Cambodia	2	2.9	0.4–17.9	0.00	0.084	1	0.772
Yemen	1	84.6	65.5–94.1	-	-	-	-
Lebanon	1	4.2	0.3–42.5	-	-	-	-
Overall	207	15.4	11.8–19.8	97.37	7819.59	206	<0.001
Genotype E							
Iran	20	1.2	0.6–2.1	0.00	11.50	19	0.906
Uzbekistan	2	0.6	0.1–4.3	0.00	0.134	1	0.715
Pakistan	12	0.4	0.2–0.7	0.00	4.15	11	0.965
Indonesia	8	0.9	0.4–2.4	0.00	2.79	7	0.904
Tajikistan	1	1.0	0.1–13.6	-	-	-	-
Japan	19	0.3	0.2–0.6	12.4	20.55	18	0.303
Bangladesh	4	2.8	0.7–10.5	0.00	1.45	3	0.693
India	21	0.9	0.4–1.8	39.63	33.13	20	0.033
Turkey	15	0.8	0.4–1.5	0.00	2.55	14	1.000
Vietnam	4	0.8	0.2–3.1	0.00	0.92	3	0.820
Thailand	8	0.6	0.2–1.7	0.00	2.34	7	0.939
China	68	0.3	0.2–17.1	0.00	58.50	67	0.939
Azerbaijan	1	0.5	0.00–7.2	-	-	-	-
Jordan	1	0.7	0.0–10.3	-	-	-	-
Malaysia	2	8.2	0.1–92.9	89.72	9.73	1	0.002
Korea	2	0.7	0.1–6.0	18.66	1.23	1	0.268
Serbia	1	0.3	0.00–4.7	-	-	-	-
Myanmar	1	0.3	0.00–5.0	-	-	-	-
Taiwan	6	0.6	0.2–1.5	0.00	3.81	5	0.577
Sri Lanka	1	4.0	0.6–23.5	-	-	-	-
Nepal	3	0.9	0.2–4.3	0.00	0.067	2	0.967
Iraq	1	5.4	2.9–9.7	-	-	-	-
Oman	1	1.2	0.3–4.6	-	-	-	-
Afghanistan	1	0.3	0.0–4.7	-	-	-	-
Cambodia	2	2.9	0.4–17.9	0.00	0.084	1	0.722
Yemen	1	1.9	0.1–23.6	-	-	-	-
Lebanon	1	4.2	0.3–42.5	-	-	-	-
Overall	207	0.6	0.5–0.7	29.79	293.39	206	<0.001
Genotype F							
Iran	20	1.2	0.6–2.1	0.00	11.49	19	0.906
Uzbekistan	2	0.6	0.1–4.3	0.00	0.134	1	0.715
Pakistan	12	0.7	0.3–1.4	21.44	14.00	11	0.233
Indonesia	8	0.8	0.3–2.1	0.00	1.99	7	0.960
Tajikistan	1	1.0	0.1–13.6	-	-	-	-
Japan	19	0.4	0.2–0.6	21.72	22.99	18	0.191
Bangladesh	4	2.8	0.7–10.5	0.00	1.45	3	0.693
India	21	0.9	0.5–1.5	0.00	13.04	20	0.876
Turkey	15	1.4	0.8–2.5	0.00	9.47	14	0.800
Vietnam	4	0.8	0.2–3.1	0.00	0.922	3	0.820
Thailand	8	0.6	0.2–1.7	0.00	2.34	7	0.94
China	68	0.3	0.2–0.4	0.00	58.17	67	0.771
Azerbaijan	1	0.5	0.00–7.2	-	-	-	-
Jordan	1	0.7	0.0–10.3	-	-	-	-
Malaysia	2	2.1	0.2–20.2	35.48	1.55	1	0.213
Korea	2	0.7	0.1–6.0	18.66	1.23	1	0.268
Serbia	1	0.3	0.00–4.7	-	-	-	-
Myanmar	1	0.3	0.00–5.0	-	-	-	-
Taiwan	6	1.8	0.4–6.9	81.25	26.66	5	<0.001
Sri Lanka	1	1.9	0.1–24.4	-	-	-	-
Nepal	3	0.9	0.2–4.3	0.00	0.067	2	0.967
Iraq	1	31.2	24.9–38.2	-	-	-	-
Oman	1	0.3	0.00–4.5	-	-	-	-
Afghanistan	1	0.3	0.00–4.7	-	-	-	-
Cambodia	2	2.9	0.4–17.9	0.00	0.084	1	0.772
Yemen	1	1.9	0.1–23.6	-	-	-	-
Lebanon	1	4.2	0.3–42.5	-	-	-	-
Overall	207	0.6	0.4–0.8	75.26	832.77	206	<0.001
Genotype G							
Iran	20	1.2	0.6–2.1	0.00	11.49	19	0.906
Uzbekistan	2	0.6	0.1–4.3	0.00	0.134	1	0.715
Pakistan	12	0.3	0.1–0.6	0.00	3.49	11	0.983
Indonesia	8	0.8	0.3–2.1	0.00	1.99	7	0.960
Tajikistan	1	1.0	0.1–13.6	-	-	-	-
Japan	19	0.2	0.1–0.4	0.00	16.04	18	0.590
Bangladesh	4	2.8	0.7–10.5	0.00	1.45	3	0.693
India	21	0.7	0.4–1.4	0.00	14.64	20	0.796
Turkey	15	0.8	0.4–1.6	0.00	2.57	14	1.000
Vietnam	4	0.8	0.2–3.1	0.00	0.922	3	0.820
Thailand	8	0.7	0.3–1.8	0.00	2.606	7	0.919
China	68	0.3	0.2–0.4	0.00	58.17	67	0.771
Azerbaijan	1	0.5	0.0–7.2	-	-	-	-
Jordan	1	0.7	0.0–10.3	-	-	-	-
Malaysia	2	2.1	0.2–20.2	35.48	1.55	1	0.213
Korea	2	0.7	0.1–6.0	18.66	1.23	1	0.268
Serbia	1	0.3	0.00–4.7	-	-	-	-
Myanmar	1	0.3	0.00–5.0	-	-	-	-
Taiwan	6	0.8	0.2–3.5	63.64	13.75	5	0.017
Sri Lanka	1	1.9	0.1–24.4	-	-	-	-
Nepal	3	0.9	0.2–4.3	0.00	0.067	2	0.967
Iraq	1	0.3	0.00–4.1	-	-	-	-
Oman	1	0.3	0.00–4.5	-	-	-	-
Afghanistan	1	0.3	0.00–4.7	-	-	-	-
Cambodia	2	2.9	0.4–17.9	0.00	0.084	1	0.772
Yemen	1	1.9	0.1–23.6	-	-	-	-
Lebanon	1	4.2	0.3–42.5	-	-	-	-
Overall	207	0.6	0.5–0.7	0.00	205.13	206	0.504
Genotype H							
Iran	20	1.2	0.6–2.1	0.0.	11.49	19	0.906
Uzbekistan	2	0.6	0.1–4.3	0.00	0.134	1	0.715
Pakistan	12	0.3	0.1–0.6	0.00	3.49	11	0.983
Indonesia	8	0.8	0.3–2.1	0.00	1.99	7	0.960
Tajikistan	1	1.0	0.1–13.6	-	-	-	-
Japan	19	0.3	0.2–0.5	3.1	18.58	18	0.418
Bangladesh	4	2.8	0.7–10.5	0.00	1.45	3	0.693
India	21	0.7	0.4–1.2	0.00	10.56	20	0.957
Turkey	15	0.8	0.4–1.5	0.00	2.55	14	1.000
Vietnam	4	0.8	0.2–3.1	0.00	0.922	3	0.820
Thailand	8	0.7	0.3–1.8	0.00	2.606	7	0.919
China	68	0.3	0.2–0.4	0.00	58.17	67	0.771
Azerbaijan	1	0.5	0.00–7.2	-	-	-	-
Jordan	1	0.7	0.00–10.3	-	-	-	-
Malaysia	2	2.1	0.2–20.2	35.48	1.55	1	0.213
Korea	2	0.7	0.1–6.0	18.66	1.23	1	0.268
Serbia	1	0.3	0.00–4.7	-	-	-	-
Myanmar	1	0.3	0.00–5.0	-	-	-	-
Taiwan	6	0.4	0.1–1.2	0.00	3.22	5	0.666
Sri Lanka	1	1.9	0.1–24.4	-	-	-	-
Nepal	3	0.9	0.2–4.3	0.00	0.067	2	0.967
Iraq	1	0.3	0.00–4.1	-	-	-	-
Oman	1	0.3	0.00–4.5	-	-	-	-
Afghanistan	1	0.3	0.00–4.7	-	-	-	-
Cambodia	2	2.9	0.4–17.9	0.00	0.084	1	0.772
Yemen	1	1.9	0.1–23.6	-	-	-	-
Lebanon	1	4.2	0.3–42.5	-	-	-	-
Overall	207	0.5	0.4–0.6	0.00	162.06	206	0.989
Genotype I							
Iran	20	1.2	0.6–2.1	0.00	11.49	19	0.906
Uzbekistan	2	0.6	0.1–4.3	0.00	0.13	1	0.715
Pakistan	12	0.3	0.1–0.6	0.00	3.49	11	0.983
Indonesia	8	0.8	0.3–2.1	0.00	1.99	7	0.960
Tajikistan	1	1.0	0.1–0.4	-	-	-	-
Japan	19	0.2	0.1–0.4	0.00	16.04	18	0.590
Bangladesh	4	2.8	0.7–10.5	0.00	1.45	3	0.693
India	21	0.9	0.5–1.4	0.00	8.73	20	0.986
Turkey	15	0.8	0.4–1.5	0.00	2.55	14	1.000
Vietnam	4	1.4	0.5–3.8	0.00	1.05	3	0.790
Thailand	8	0.6	0.2–1.7	0.00	2.34	7	0.939
China	68	0.3	0.2–0.5	33.64	100.96	67	0.005
Azerbaijan	1	0.5	0.00–7.2	-	-	-	-
Jordan	1	0.7	0.00–10.3	-	-	-	-
Malaysia	2	2.1	0.2–20.2	35.48	1.55	1	0.213
Korea	2	0.7	0.1–6.0	18.66	1.23	1	0.268
Serbia	1	0.3	0.00–4.7	-	-	-	-
Myanmar	1	0.3	0.00–5.0	-	-	-	-
Taiwan	6	0.4	0.1–1.2	0.00	3.22	5	0.666
Sri Lanka	1	1.9	0.1–24.4	-	-	-	-
Nepal	3	0.9	0.2–4.3	0.00	0.067	2	0.967
Iraq	1	0.3	0.00–4.1	-	-	-	-
Oman	1	0.3	0.00–4.5	-	-	-	-
Afghanistan	1	0.3	0.00–4.7	-	-	-	-
Cambodia	2	2.9	0.4–17.9	0.00	0.084	1	0.772
Yemen	1	1.9	0.1–23.6	-	-	-	-
Lebanon	1	4.2	0.3–42.5	-	-	-	-
Overall	207	0.6	0.5–0.8	0.00	190.91	206	0.767

## Data Availability

The data presented in this study are available in the supplementary material.

## Supplementary Materials

The following supporting information can be downloaded at: https://www.mdpi.com/article/10.3390/healthcare11071011/s1, S1: Search Strategy for Hbv Meta-Analysis; S3: Jbi Checklist for Prevalence Data; Table S4: Quality of Included Studies by Jbi Critical Appraisal Checklist for Studies Reporting Prevalence Data; Table S5: data; Table S6: Data Extraction Sheet for Hbv Subtypes in Asia; S7: supplementary file; S8: Subgroup forest plot by country; Table S9: Subgroup Analysis for Comparison of Prevalence of Hbv Genotypes in Asia Across Targeted Gene(s) Of Detection; Table S10: Subgroup analysis for comparison of prevalence of HBV genotypes in Asia across the methods of genotyping; File FP1 to FP3.
