# Supplementary material for: A Recent Prevalence of Hepatitis B Virus (HBV) Genotypes and Subtypes in Asia: A Systematic Review and Meta-Analysis
Source: Healthcare (Basel). 2023 Apr 1;11(7):1011. doi: 10.3390/healthcare11071011 (PMC10094200; doi:10.3390/healthcare11071011)
Supplement: Supplementary file 1 [file healthcare-11-01011-s001.zip › healthcare-2189410-supplementary.pdf]

Table S9. Subgroup analysis for comparison of prevalence of HBV genotypes in Asia across targeted gene(s) of detection

Target gene	Number of studies	Prevalence (%)	95% CI	I^2 (%)	Q	Heterogeneity test	
						DF	*P*
Genotype A							
P	10	2.6	0.9 – 7.7	79.5	43.90	9	<0.001
S	135	3.2	2.4 – 4.2	92.73	1842.33	134	<0.001
S and C	38	2.8	1.7 – 4.4	89.01	336.53	37	<0.001
C	6	14.8	8.0 – 25.8	73.62	18.96	5	0.002
P and C	1	0.3	0.0 – 4.6	-	-	-	-
P, S and C	5	0.7	0.2 – 3.1	64.59	11.30	4	0.023
P and S	9	4.1	1.5 – 10.6	93.33	119.88	8	<0.001
S, C and X	1	0.6	0.0 – 8.4	-	-	-	-
X	2	0.4	0.1 – 2.6	0.00	0.098	1	0.754
Overall	207	3.1	0.1 – 2.6	91.79	2507.63	206	<0.001
Genotype B							
P	10	2.5	0.2 – 20.4	94.96	178.43	9	<0.001
S	135	20.1	16.9 – 23.7	97.46	5271.55	134	<0.001
S and C	38	14.3	10.5 – 19.1	95.5	822.79	37	<0.001
C	6	7.6	1.2 – 35.6	89.94	49.70	5	<0.001
P and C	1	0.3	0.00 – 4.6	-	-	-	-
P, S and C	5	28.9	18.1 – 42.8	89.95	39.78	4	<0.001
P and S	9	26.2	14.0 – 43.6	97.87	376.17	8	<0.001
S, C and X	1	0.6	0.0 – 8.4	-	-	-	-
X	2	15.5	1.9 – 63.0	97.09	34.33	1	<0.001
Overall	207	17.8	15.5 – 20.4	97.26	7524.23	206	<0.001
Genotype C							
P	10	10.8	4.5 – 24.0	90.29	92.73	9	<0.001
S	135	30.2	26.2 – 34.5	97.56	5500.91	134	<0.001
S and C	38	37.1	29.0 – 46.0	96.82	1163.0	37	<0.001
C	6	30.6	9.8 – 64.1	95.77	118.34	5	<0.001
P and C	1	0.3	0.00 – 4.6	-	-	-	-
P, S and C	5	36.6	21.0 – 55.6	93.96	66.19	4	<0.001
P and S	9	37.6	18.0 – 62.3	98.62	579.36	8	<0.001
S, C and X	1	0.6	0.00 – 8.4	-	-	-	-
X	2	82.6	40.3 – 97.1	96.79	31.15	1	<0.001
Overall	207	30.9	27.5 – 34.5	97.57	8476.52	206	<0.001
Genotype D							
P	10	79.0	53.8 – 92.4	90.16	91.45	9	<0.001
S	135	13.9	10.1 – 18.8	97.0	4467.03	134	<0.001
S and C	38	13.9	6.9 – 26.1	98.29	2167.86	37	<0.001
C	6	44.5	24.1 – 67.0	88.47	43.38	5	<0.001
P and C	1	99.7	95.4 – 100.0	-	-	-	-
P, S and C	5	25.7	0.6 – 95.0	94.86	77.75	4	<0.001
P and S	9	1.6	0.1 – 16.5	98.11	422.56	8	<0.001
S, C and X	1	99.4	91.6 – 100.0	-	-	-	-
X	2	0.4	0.1 – 2.6	0.00	0.098	1	0.754
Overall	207	15.4	11.8 – 19.8	97.37	7819.59	206	<0.001
Genotype E							
P	10	0.8	0.3 – 2.0	0.00	6.11	9	0.729
S	135	0.6	0.4 – 0.8	41.15	227.69	134	<0.001
S and C	38	0.5	0.3 – 0.8	0.00	31.52	37	0.723
C	6	0.9	0.3 – 2.9	0.00	3.75	5	0.586
P and C	1	0.3	0.00 – 4.6	-	-	-	-
P, S and C	5	0.3	0.1 – 1.1	0.00	3.37	4	0.500
P and S	9	0.4	0.2 – 1.1	23.55	10.47	8	0.234
S, C and X	1	0.6	0.00 – 8.4	-	-	-	-
X	2	0.4	0.1 – 2.6	0.00	0.098	1	0.754
Overall	207	0.6	0.5 – 0.7	29.79	29340	206	<0.001
Genotype F							
P	10	0.8	0.3 – 0.20	0.00	6.11	9	0.729
S	135	0.6	0.4 – 0.9	80.14	674.59	134	<0.001
S and C	38	0.5	0.3 – 0.8	0.00	31.84	37	0.709
C	6	0.9	0.3 – 2.9	0.00	3.75	5	0.586
P and C	1	0.3	0.1 – 1.1	-	-	-	-

P, S and C	5	0.3	0.1 – 1.1	0.00	3.356	4	0.500
P and S	9	0.3	0.1 – 0.8	0.72	8.06	8	0.428
S, C and X	1	0.6	0.00 – 8.4	-	-	-	-
X	2	0.4	0.1 – 2.6	0.00	0.098	1	0.754
Overall	207	0.6	0.4 – 0.8	75.26	832.73	206	<0.001
Genotype G							
P	10	0.9	0.4 – 2.1	0.00	6.04	9	0.736
S	135	0.5	0.4 – 0.7	5.19	141.34	134	0.315
S and C	38	0.6	0.4 – 0.9	1.06	37.40	37	0.451
C	6	0.9	0.3 – 2.9	0.00	3.75	5	0.586
P and C	1	0.3	0.00 – 4.6	-	-	-	-
P, S and C	5	0.3	0.00 – 1.1	0.00	3.36	4	0.500
P and S	9	0.3	0.1 – 0.8	0.72	8.06	8	0.428
S, C and X	1	0.6	0.0 – 8.4	-	-	-	-
X	2	0.4	0.1 – 2.6	0.00	0.098	1	0.754
Overall	207	0.6	0.5 – 0.7	0.00	205.13	206	0.504
Genotype H							
P	10	0.8	0.3 – 2.0	0.00	6.11	9	0.729
S	135	0.5	0.4 – 0.6	0.00	103.58	134	0.976
S and C	38	0.5	0.3 – 0.8	0.00	32.52	37	0.679
C	6	0.9	0.3 – 2.9	0.00	3.75	5	0.586
P and C	1	0.3	0.00 – 4.6	-	-	-	-
P, S and C	5	0.3	0.1 – 1.1	0.00	3.36	4	0.500
P and S	9	0.4	0.2 – 1.0	12.18	9.11	8	0.333
S, C and X	1	0.6	0.00 – 8.4	-	-	-	-
X	2	0.4	0.1 – 2.6	0.00	0.098	1	0.754
Overall	207	0.5	0.4 – 0.6	0.00	162.06	206	0.989
Genotype I							
P	10	0.8	0.3 – 2.0	0.00	6.11	9	0.729
S	135	0.6	0.4 – 0.7	0.00	114.47	134	0.888
S and C	38	0.5	0.3 – 0.8	0.00	30.55	37	0.764
C	6	0.9	0.3 – 2.9	0.00	3.75	5	0.586
P and C	1	0.3	0.0 – 4.6	-	-	-	-
P, S and C	5	1.6	1.0 – 2.4	0.00	3.69	4	0.449
P and S	9	0.3	0.1 – 0.8	0.72	8.06	8	0.428
S, C and X	1	0.6	0.00 – 8.4	-	-	-	-
X	2	0.4	0.1 – 2.6	0.00	0.098	1	0.754
Overall	207	0.6	0.5 – 0.8	0.00	190.91	206	0.767

P: Polymerase gene, S: Surface protein gene, C: Core protein gene, X: Regulatory protein gene

name of auth	year of public	country	target region	method of ge	total genotye	A
Abbas et al	2009	Iran	P	sequencing	6	0
Abbasian et a	2021	Iran	P	sequencing	8	0
Abdulkadirov	2020	Uzbekstan	S	multiplex PCF	113	4
Aftab et al	2018	Pakistan	S	sequencing	39	13
Aghakhani et	2009	Iran	S	sequencing	40	0
Alam. et al	2007	Pakistan	S	sequencing	56	15
Alam et al	2007	Pakistan	S	multiplex PCF	110	5
Alavian et al	2006	Iran	P	LIPA	109	0
Anvari et al	2016	Iran	S	RFLP	2	0
Andi. et al	2009	Indonesia	S	sequencing	214	131
Andi et al	2009	Indonesia	S	sequencing	171	0
Anis et al	2008	Tajikistan	S and C	sequencing	51	3
Anvari et al	2013	Iran	S	sequencing	29	0
Aono et al	2007	Japan	S	sequencing	48	11
Arabadadi et	2011	Iran	S	sequencing	57	0
Arifur et al	2016	Bangledesh	S	sequencing	17	3
Arup et al	2006	India	C	RFLP	236	66
Ashrafali et a	2014	India	S	sequencing	295	53
Atalay et al	2011	Turkey	S	sequencing	107	0
Attaran et al	2017	Iran	P and C	sequencing	166	0
Avik. et al	2013	India	P	sequencing	47	4
Avik et al	2009	India	S and C	RFLP	141	29
Babana et al	2017	Pakistan	S	sequencing	337	9
Bhagirathi et	2014	India	S and C	sequencing	17	0
Borkakoty et	2008	India	S	sequencing	29	15
Bozdayo et al	2003	Turkey	S	RFLP	58	0
Brij et al	2018	India	S	sequencing	17	0
Bui et al	2017	Vietnam	S and C	sequencing	135	0
Chamni et al	2014	Thailand	S and C	sequencing	47	0
Chan et al	2002	China	S	sequencing	42	0
Chandana et	2011	India	S	sequencing	71	6
Choudhardy e	2008	Pakistan	S	sequencing	236	2
Ciftci et al	2012	Turkey	P, S and C	sequencing	63	0
Cuong et al	2011	Vietnam	S	sequencing	113	0
D-D Li et al	2010	China	P and S	multiplex PCF	376	0
Davod et al	2019	Iran	S, C and X	sequencing	87	0
Debraj et al	2014	India	S	sequencing	59	19
Ding. et al	2004	China	S	RFLP	166	0
Ding.. et al	2013	China	S	RDB hybridiza	250	0
Ding et al.	2002	China	S	sequencing	141	6
Ding et al	2001	China	S and C	RFLP	220	3
Dong. et al	2009	China	S and C	sequencing	51	0

Dong.. et al	2021	China	S and C	sequencing	649	0
Dong et al	2009	China	S	sequencing	692	6
Du et al	2006	China	S	LIPA	121	0
Edward et al	2005	China	S	multiplex PCF	15	0
Emekdas et a	2012	Turkey	S and C	RFLP	34	0
Etsuro et al	2001	Japan	S	RFLP	720	12
Fang et al	2004	China	S and C	sequencing	36	0
Farah et al	2014	Azerbaijan	S	LIPA	103	6
Farooq et al	2021	Pakistan	P	sequencing	98	21
Fei et al	2009	China	S	sequencing	127	0
Fujie et al	2001	Japan	S	RFLP	109	3
Fujiko et al	2015	Indonesia	S	sequencing	47	0
Fuminaka et a	2002	Thailand	S	LIPA	107	0
Furusyo et al	2004	Japan	S	sequencing	409	0
Ghani et al	2011	Pakistan	S	multiplex PCF	173	43
Gollapudi et a	2008	India	S and C	sequencing	14	6
Gu et al	2004	China	S	multiplex PCF	464	24
Haimanti et a	2014	India	S and C	sequencing	34	0
Haixia et al	2013	China	S	sequencing	7	0
Hakim et al	2016	Pakistan	S and C	sequencing	180	0
Han et al	2009	China	S	sequencing	255	0
Hani et al	2008	Jordan	S	LIPA	70	0
Hayashi et al	2007	Japan	P and S	sequencing	246	30
Hideaki et al	2002	Uzbekstan	S	RFLP	54	7
Hong mei et a	2015	China	P, S and C	sequencing	1148	1
Hooi et al	2005	Malaysia	S	sequencing	6	0
Hu et al	2004	China	S	RFLP	424	0
Hudu et al	2018	Malaysia	S	sequencing	82	0
Hyun-Ju et al	2007	Korea	S	sequencing	209	0
Inui et al	2007	Japan	S	sequencing	116	0
Ivana et al	2013	Serbia	S	sequencing	162	23
Jagdish et al	2020	India	S	sequencing	67	20
Jin et al	2010	China	X	sequencing	182	0
Kailash et al	2011	India	S	multiplex PCF	150	81
Kaklikkaya et	2012	Turkey	S	RFLP	125	0
Kamol et al	2007	Thailand	S	sequencing	147	2
Kana et al	2007	Japan	S	RFLP	508	9
Karimi et al	2014	Iran	S	multiplex PCF	23	6
Kaya et al	2007	Turkey	S	LIPA	124	6
Kazuhiko et a	2009	Vietnam	S and C	sequencing	27	0
kikuchi et al	2000	Japan	P, S and C	sequencing	310	8
Kobayashi et	2002	Japan	S	LIPA	48	13
Komatsu et a	2015	Japan	S	LIPA	261	8

Kwon et al	2018	Korea	S and C	sequencing	22	0
Kyaw et al	2020	Myanmar	S	sequencing	153	0
Leblebicioglu	2004	Turkey	S	RFLP	147	0
Lee et al	2003	Taiwan	S	RFLP	265	3
Lei et al	2005	China	S	multiplex PCF	182	0
Li et al	2010	China	X	sequencing	97	0
Li. et al	2011	China	P and S	sequencing	1301	0
Li ..et al	2011	China	S and C	sequencing	218	0
Li et al.	2007	China	S	multiplex PCF	180	0
Li et al..	2006	China	S	multiplex PCF	142	0
Lin et al	2007	Taiwan	S	sequencing	125	0
Liu. et al	2012	China	S	multiplex PCF	208	9
Liu.. et al	2004	Taiwan	C	RFLP	17	0
Liu et al.	2002	Taiwan	S	RFLP	85	1
Liu et al	2010	China	P and S	sequencing	5	0
Lu. et al	2007	China	S	sequencing	90	0
Lu.. et al	2007	China	S	multiplex PCF	127	0
Lu et al	2011	China	S	multiplex PCF	36	0
Lusida et al	2003	Indonesia	S	LIPA	54	0
Ma et al	2011	China	S and C	sequencing	100	0
Mahmood et	2016	Pakistan	S and C	multiplex PCF	715	55
Manamperi e	2011	Sri Lanka	S	multiplex PCF	25	2
Matsuura et a	2009	Japan	S	RFLP	1271	44
Modhusudon	2016	Bangledesh	S	sequencing	19	0
Mojiri et al	2008	Iran	S	sequencing	89	0
Mousavi. et a	2014	Iran	S	RFLP	17	0
Mousavi et al	2014	Iran	S	sequencing	122	0
Nei et al	2012	China	S	sequencing	556	0
Neisi et al	2011	iran	S and C	sequencing	48	0
Nguyen et al	2008	China	S	sequencing	199	2
Ni et al	2004	Taiwan	S	multiplex PCF	460	0
Norouzi et al	2012	Iran	S	sequencing	19	0
Ogawa et al	2002	Japan	S	sequencing	25	14
Oguzoglu et a	2001	Turkey	S	RFLP	48	5
Onganar et	2006	Turkey	S	RFLP	50	6
Ozdemir et al	2005	Turkey	S	sequencing	54	0
Panicker et al	2021	India	S	sequencing	144	8
Parichat et al	2015	Thailand	S and C	sequencing	111	0
Pattaratida et	2010	Thailand	S and C	sequencing	224	1
Paudel et al	2015	Nepal	S	sequencing	42	17
Perumal et al	2004	India	C	RFLP	167	30
Pinarbasi et a	2008	Turkey	S and C	sequencing	13	0
Prapan et al	2008	Thailand	P	multiplex PCF	53	0

Premashis et	2021	India	S	sequencing	655	34
Priyo et al	2016	Indonesia	S	sequencing	61	0
Rafatpanah e	2008	Iran	S	sequencing	70	0
Rana et al	2019	Iraq	S	multiplex PCF	186	20
Retno et al	1991	Indonesia	S	sequencing	47	4
Saeeda. et al	2008	Pakistan	S	sequencing	201	47
Saeeda et al	2007	Pakistan	S and C	sequencing	295	60
Sahapat et al	2012	Thailand	S	sequencing	15	0
Said et al	2014	Oman	P and S	sequencing	170	32
Saif et al	2017	Bangledesh	S and C	sequencing	53	10
Saket. et al	2006	India	S and C	RFLP	160	12
Saket et al	2006	India	S	RFLP	100	16
Salman et al	2019	India	S and C	sequencing	17	6
Samad et al	2009	Iran	S	multiplex PCF	125	0
Santosh et al	2006	Nepal	S	sequencing	71	16
Sayan & Doga	2012	Turkey	P	sequencing	94	0
Serin et al	2005	Turkey	S	sequencing	47	0
Shahnaz et al	2018	Iran	S	sequencing	26	0
Shamima et a	2017	Bangledesh	S	multiplex PCF	4	2
Shen et al	2015	China	P, S and C	sequencing	57	0
Shi et al	2012	China	C	sequencing	89	0
Smita et al	2020	Nepal	S and C	sequencing	56	4
Sobia et al	2011	Afghanistan	S	multiplex PCF	161	33
Song. et al	2005	China	S	sequencing	480	8
Song et al	2006	China	S	RDB hybridiza	96	0
Srey et al	2006	Cambodia	S	sequencing	22	0
Su et al	2020	China	S	sequencing	369	0
Suda et al	2012	Thailand	S and C	sequencing	86	0
Sugauchi et a	2006	Japan	S and C	RFLP	485	92
Sunbul and Le	2005	Turkey	P, S and C	RFLP	78	0
Sunbul et al	2003	Turkey	S	RFLP	86	0
Suzuki et al	2003	Japan	S and C	sequencing	234	8
Swati et al	2003	India	S	sequencing	62	5
Takako et al	2009	Indonesia	S	sequencing	21	0
Talal et al	2004	Yemen	C	sequencing	26	4
Tamada et al	2011	Japan	S	sequencing	547	136
Tanaka et al	2009	Japan	S	sequencing	1887	106
Tang et al	2007	China	S	sequencing	400	0
Tao et al	2019	China	S	RDB hybridiza	1204	0
Thanh et al	2005	Vietnam	S	sequencing	38	0
Torii et al	2013	Japan	S	sequencing	10	3
Tran et al	2008	Cambodia	C	sequencing	12	0
Usuda et al	1999	Japan	S	LIPA	507	24

Wada et al	2014	Japan	S	sequencing	90	27
Wahyuni et a	2019	Indonesia	S	sequencing	41	0
Wang. et al	2014	China	S	sequencing	71	0
Wang.. et al	2012	China	P and S	sequencing	142	0
Wang et al	2018	China	S	sequencing	73	0
Wei et al	2015	China	S	sequencing	6817	0
Wen and Tan	2004	China	S	multiplex PCF	220	0
Wu et al	2012	China	S	LIPA	319	0
Xia et al	2001	China	S and C	sequencing	280	0
Xiang et al	2012	China	S	sequencing	112	0
Xin et al	2002	China	S	RFLP	135	6
Yan. et al	2010	China	S	sequencing	60	0
yahya et al	2010	Iran	S	sequencing	100	0
Yan et al	2015	China	P and S	multiplex PCF	258	11
Yanag et al	2017	China	S	sequencing	178	0
Yashwant et a	2017	India	P	sequencing	98	4
Ye. et al	2017	China	S	sequencing	9	0
Ye et al	2021	China	S and C	sequencing	14	0
Yeh et al	2004	Taiwan	S	RFLP	213	23
Yin et al	2010	China	S and C	sequencing	3013	0
You et al	2008	China	P and S	RDB hybridiza	126	0
youyun et al	2016	China	P	LIPA	269	0
Zaatari et al	2007	Lebanon	S	RFLP	11	0
Zang. et al	2009	China	S and C	sequencing	269	0
Zang et al	2015	China	S	sequencing	1221	0
Zeng et al	2005	China	S and C	sequencing	1096	13
Zhang et al	2018	China	S	sequencing	232	0
Zhao et al	2001	China	S and C	RDB hybridiza	20	0
Zhili et al	2014	China	S	multiplex PCF	1124	0
Zhong et al	2005	China	S and C	LIPA	131	0
Zhu et al	2008	China	S	multiplex PCF	733	0
Zhu. et al	2008	China	S	sequencing	166	0
Zhu.. et al	2015	China	S and C	sequencing	223	0
Zhuang et al	2007	China	P and S	RDB hybridiza	117	48
Zohreh	2013	Iran	P	sequencing	134	0
Zunaira et al	2010	Pakistan	S	sequencing	269	43
					49279	1786

B	C	D	E	F	G	H	
0	0	6	0	0	0	0	
0	0	7	0	0	0	0	
0	0	109	0	0	0	0	
0	0	26	0	0	0	0	
0	0	40	0	0	0	0	
0	0	35	0	0	0	0	
27	0	66	0	0	0	0	
0	0	109	0	0	0	0	
0	0	2	0	0	0	0	
54	2	0	0	0	0	0	
126	45	0	0	0	0	0	
0	0	48	0	0	0	0	
0	0	29	0	0	0	0	
11	26	0	0	0	0	0	
0	0	57	0	0	0	0	
0	6	4	0	0	0	0	
0	69	140	0	0	0	0	
0	67	175	0	0	0	0	
0	0	107	0	0	0	0	
0	0	166	0	0	0	0	
0	2	41	0	0	0	0	
0	33	79	0	0	0	0	
0	42	286	0	0	0	0	
0	0	17	0	0	0	0	
0	10	4	0	0	0	0	
0	0	58	0	0	0	0	
0	13	4	0	0	0	0	
98	37	0	0	0	0	0	
5	40	2	0	0	0	0	
11	31	0	0	0	0	0	
0	0	59	0	0	0	0	
0	14	220	0	0	0	0	
0	0	63	0	0	0	0	
91	20	0	0	0	0	0	
218	60	0	0	0	0	0	
0	0	87	0	0	0	0	
0	6	34	0	0	0	0	
138	28	0	0	0	0	0	
165	85	0	0	0	0	0	
20	113	2	0	0	0	0	
38	179	0	0	0	0	0	
6	45	0	0	0	0	0	

138	497	12	0	0	0	0
449	233	5	0	0	0	0
40	77	2	0	0	0	0
11	3	0	0	0	0	0
0	0	34	0	0	0	0
88	610	3	0	7	0	0
0	36	0	0	0	0	0
0	0	96	0	0	0	0
0	19	58	0	0	0	0
10	58	59	0	0	0	0
26	86	0	0	0	0	0
8	39	0	0	0	0	0
27	77	3	0	0	0	0
0	184	224	0	0	0	0
48	57	21	0	4	0	0
0	0	7	0	0	1	0
33	358	11	0	0	0	0
0	0	34	0	0	0	0
0	3	0	0	0	0	0
0	0	151	0	0	0	0
94	160	1	0	0	0	0
0	0	70	0	0	0	0
29	183	2	0	0	0	2
0	0	47	0	0	0	0
320	739	26	0	0	0	0
0	2	1	3	0	0	0
273	100	51	0	0	0	0
57	25	0	0	0	0	0
0	209	0	0	0	0	0
11	101	3	1	0	0	0
0	0	139	0	0	0	0
0	5	42	0	0	0	0
10	169	0	0	0	0	0
10	15	32	9	3	0	0
0	0	125	0	0	0	0
17	128	0	0	0	0	0
23	446	30	0	0	0	0
17	0	0	0	0	0	0
0	2	0	0	1	0	0
22	5	0	0	0	0	0
92	210	0	0	0	0	0
8	25	1	1	0	0	0
23	224	4	0	2	0	0

12	10	0	0	0	0	0
2	102	49	0	0	0	0
0	0	147	0	0	0	0
158	90	7	0	0	0	0
140	42	0	0	0	0	0
35	62	0	0	0	0	0
190	1096	15	0	0	0	0
141	77	0	0	0	0	0
40	135	0	0	0	0	0
13	125	4	0	0	0	0
55	29	0	0	0	0	0
134	50	15	0	0	0	0
14	3	0	0	0	0	0
69	10	0	0	5	0	0
5	0	0	0	0	0	0
57	30	3	0	0	0	0
28	21	50	0	0	0	0
28	8	0	0	0	0	0
54	0	0	0	0	0	0
22	50	28	0	0	0	0
4	6	509	1	0	0	0
9	4	3	1	0	0	0
179	1046	2	0	0	0	0
3	2	14	0	0	0	0
0	0	89	0	0	0	0
0	0	17	0	0	0	0
7	0	91	0	0	0	0
72	438	0	0	0	0	0
2	0	46	0	0	0	0
123	72	1	1	0	0	0
335	62	1	0	0	0	0
0	0	19	0	0	0	0
5	6	0	0	0	0	0
0	0	41	0	2	0	0
0	0	42	0	2	0	0
0	0	54	0	0	0	0
0	0	100	0	0	0	0
0	111	0	0	0	0	0
25	194	1	0	0	0	0
0	0	23	0	0	0	0
0	14	121	0	0	0	0
0	0	13	0	0	0	0
4	48	0	0	0	0	0

0	50	570	0	0	0	0
42	19	0	0	0	0	0
0	0	70	0	0	0	0
58	16	24	10	58	0	0
14	21	7	1	0	0	0
0	0	128	0	0	0	0
0	0	208	0	0	0	0
4	11	0	0	0	0	0
0	2	130	2	0	0	0
0	24	19	0	0	0	0
0	0	148	0	0	0	0
0	0	84	0	0	0	0
11	0	0	0	0	0	0
0	0	125	0	0	0	0
1	4	50	0	0	0	0
0	0	93	0	0	1	0
0	0	47	0	0	0	0
0	0	26	0	0	0	0
1	1	0	0	0	0	0
34	12	0	0	0	0	0
11	78	0	0	0	0	0
3	5	40	0	0	0	0
12	55	61	0	0	0	0
360	112	0	0	0	0	0
34	60	0	0	0	0	0
6	16	0	0	0	0	0
166	135	22	0	0	0	0
9	75	0	0	0	1	1
58	330	5	0	0	0	0
0	0	78	0	0	0	0
0	0	86	0	0	0	0
21	203	0	0	0	0	0
0	0	57	0	0	0	0
9	9	3	0	0	0	0
0	0	22	0	0	0	0
48	359	0	0	0	0	0
581	1181	15	2	2	0	0
33	333	0	0	0	0	0
828	336	1	0	0	0	0
30	7	0	0	0	0	0
0	7	0	0	0	0	0
4	8	0	0	0	0	0
196	282	2	3	0	0	0

5	58	0	0	0	0	0
35	6	0	0	0	0	0
24	45	0	0	0	0	0
12	130	0	0	0	0	0
51	22	0	0	0	0	0
3384	3430	0	0	0	0	0
190	30	0	0	0	0	0
137	182	0	0	0	0	0
82	190	8	0	0	0	0
85	27	0	0	0	0	0
20	113	2	0	0	0	0
45	15	0	0	0	0	0
0	0	100	0	0	0	0
92	155	0	0	0	0	0
0	178	0	0	0	0	0
0	18	76	0	0	0	0
8	1	0	0	0	0	0
11	2	1	0	0	0	0
61	62	31	2	25	9	0
767	2059	46	0	0	0	0
48	69	1	0	0	0	0
219	48	0	0	0	0	0
0	11	0	0	0	0	0
0	10	0	0	0	0	0
429	559	204	0	0	0	0
449	575	47	0	0	0	0
25	207	0	0	0	0	0
0	20	0	0	0	0	0
848	276	0	0	0	0	0
9	15	33	0	0	0	0
169	526	0	0	0	0	0
36	130	0	0	0	0	0
112	111	0	0	0	0	0
64	1	2	0	0	0	0
0	0	134	0	0	0	0
54	83	39	2	4	0	0
14902	23116	7726	39	115	12	3

I	A/B	B/C	C/D	A/D	B/D	E/D
0	0	0	0	0	0	0
0	0	0	0	1	0	0
0	0	0	0	0	0	0
0	0	0	0	0	0	0
0	0	0	0	0	0	0
0	0	0	0	16	0	0
0	3	0	0	0	1	0
0	0	0	0	0	0	0
0	0	0	0	0	0	0
0	0	27	0	0	0	0
0	0	45	0	0	0	0
0	0	0	0	0	0	0
0	0	0	0	0	0	0
0	0	0	0	0	0	0
0	0	0	0	0	0	0
0	0	0	2	3	0	0
0	0	0	0	0	0	0
0	0	0	0	0	0	0
0	0	0	0	0	0	0
0	0	0	0	0	0	0
0	0	0	0	0	0	0
0	0	0	0	0	0	0
0	0	0	0	0	0	0
0	0	0	0	0	0	0
0	0	0	0	0	0	0
0	0	0	0	0	0	0
0	0	0	0	0	0	0
0	0	0	0	0	0	0
0	0	0	0	0	0	0
0	0	0	0	0	0	0
0	0	0	0	6	0	0
0	0	0	0	0	0	0
0	0	0	0	0	0	0
2	0	0	0	0	0	0
0	0	98	0	0	0	0
0	0	0	0	0	0	0
0	0	0	0	0	0	0
0	0	0	0	0	0	0
0	0	0	0	0	0	0
0	0	0	0	0	0	0
0	0	0	0	0	0	0
0	0	0	0	0	0	0

2	0	0	0	0	0	0
0	0	0	0	0	0	0
0	0	2	0	0	0	0
0	0	1	0	0	0	0
0	0	0	0	0	0	0
0	0	0	0	0	0	0
0	0	0	0	0	0	0
0	0	0	0	1	0	0
0	0	0	0	0	0	0
0	0	0	0	0	0	0
0	0	0	0	0	0	0
0	0	0	0	0	0	0
0	0	0	0	0	0	0
0	0	0	0	0	0	0
0	0	0	0	0	0	0
0	0	0	0	0	0	0
0	0	14	0	0	0	0
0	0	0	0	0	0	0
0	0	0	4	0	0	0
0	0	0	1	0	28	0
0	0	0	0	0	0	0
0	0	0	0	0	0	0
0	0	0	0	0	0	0
0	0	0	0	0	0	0
20	0	0	42	0	0	0
0	0	0	0	0	0	0
0	0	0	0	0	0	0
0	0	0	0	0	0	0
0	0	0	0	0	0	0
0	0	0	0	0	0	0
0	0	0	0	0	0	0
0	0	0	0	0	0	0
0	0	3	0	0	0	0
0	0	0	0	0	0	0
0	0	0	0	0	0	0
0	0	0	0	0	0	0
0	0	0	0	0	0	0
0	0	0	0	0	0	0
0	0	0	0	0	0	0
0	0	0	0	0	0	0
0	0	0	0	0	0	0
0	0	0	0	0	0	0
0	0	0	0	0	0	0

0	0	0	0	0	0	0
0	0	0	0	0	0	0
0	0	0	0	0	0	0
0	0	0	0	0	0	0
0	0	0	0	0	0	0
0	0	0	0	0	0	0
0	0	0	0	0	0	0
0	0	0	0	0	0	0
0	0	5	0	0	0	0
0	0	0	0	0	0	0
0	0	41	0	0	0	0
0	0	0	0	0	0	0
0	0	0	0	0	0	0
0	0	0	0	0	0	0
0	0	0	0	0	0	0
0	0	0	0	0	0	0
0	0	0	11	0	12	0
0	0	0	0	0	0	0
0	0	0	0	0	0	0
0	0	0	0	0	0	0
0	2	0	7	124	14	1
0	0	3	0	1	2	0
0	0	0	0	0	0	0
0	0	0	0	0	0	0
0	0	0	0	0	0	0
0	0	0	0	0	0	0
0	0	0	0	0	27	0
0	0	0	0	0	0	0
0	0	0	0	0	0	0
0	0	0	0	0	0	0
0	0	0	0	0	0	0
0	0	0	0	0	0	0
0	0	0	0	0	0	0
0	0	0	0	0	0	0
0	0	0	0	0	0	0
0	0	0	0	0	0	0
0	0	2	16	3	1	0
0	0	0	0	0	0	0
0	0	2	1	0	0	0
0	0	0	2	0	0	0
0	0	0	0	2	0	0
0	0	0	0	0	0	0
0	0	1	0	0	0	0

7	0	0	0	0	0	0
0	0	0	0	0	0	0
0	0	0	0	0	0	0
0	0	0	0	0	0	0
0	0	0	0	0	0	0
0	0	0	0	26	0	0
0	0	0	0	27	0	0
0	0	0	0	0	0	0
0	0	0	1	3	0	0
0	0	0	0	0	0	0
0	0	0	0	0	0	0
0	0	0	0	3	0	0
0	0	0	0	0	0	0
0	0	0	0	0	0	0
0	0	0	0	0	0	0
0	0	0	0	0	0	0
0	0	0	0	0	0	0
0	0	0	0	0	0	0
0	0	0	0	0	0	0
0	0	11	0	0	0	0
0	0	0	0	0	0	0
0	0	4	0	0	0	0
0	0	0	0	0	0	0
0	0	0	0	0	0	0
0	0	2	0	0	0	0
0	0	0	0	0	0	0
0	0	44	36	0	0	0
0	0	0	0	0	0	0
0	0	0	0	0	0	0
0	0	0	0	0	0	0
0	0	0	0	0	0	0
0	0	0	0	0	0	0
0	0	0	0	0	0	0
0	0	0	0	0	0	0
0	0	0	0	0	0	0
0	0	4	0	0	0	0
0	0	0	0	0	0	0
0	0	34	0	0	0	0
0	0	18	0	0	5	0
0	0	1	0	0	0	0
0	0	0	0	0	0	0
0	0	0	0	0	0	0
0	0	0	0	0	0	0

0	0	0	0	0	0	0
0	0	0	0	0	0	0
2	0	0	0	0	0	0
0	0	0	0	0	0	0
0	0	0	0	0	0	0
0	0	0	0	0	0	0
0	0	0	0	0	0	0
0	0	0	0	0	0	0
0	0	0	0	0	0	0
0	0	0	0	0	0	0
0	0	1	0	0	0	0
0	0	0	0	0	0	0
0	0	0	0	0	0	0
0	0	0	0	0	0	0
0	0	0	0	0	0	0
0	0	0	0	0	0	0
0	0	0	0	0	0	0
0	0	0	0	0	0	0
0	0	0	0	0	0	0
0	0	0	0	0	0	0
0	0	2	0	0	0	0
0	0	2	0	0	0	0
0	0	0	0	0	0	0
0	0	0	259	0	0	0
0	0	0	29	0	0	0
0	0	0	0	0	0	0
0	0	0	0	0	0	0
0	0	0	0	0	0	0
0	0	0	0	0	0	0
0	0	16	43	0	15	0
0	0	0	0	0	0	0
0	0	0	0	0	0	0
0	0	0	0	0	0	0
0	0	2	0	0	0	0
0	0	0	0	0	0	0
0	0	9	0	16	0	0
33	5	394	454	232	105	1

B/E	A/F	A/E	A/C	A/B/C	B/D/F	A/C/D
0	0	0	0	0	0	0
0	0	0	0	0	0	0
0	0	0	0	0	0	0
0	0	0	0	0	0	0
0	0	0	0	0	0	0
0	0	0	0	0	0	0
0	0	0	0	0	0	0
0	0	0	0	0	0	0
0	0	0	0	0	0	0
0	0	0	0	0	0	0
0	0	0	0	0	0	0
0	0	0	0	0	0	0
0	0	0	0	0	0	0
0	0	0	0	0	0	0
0	0	0	0	0	0	0
0	0	0	0	0	0	0
0	0	0	0	0	0	0
0	0	0	0	0	0	0
0	0	0	0	0	0	0
0	0	0	0	0	0	0
0	0	0	0	0	0	0
0	0	0	0	0	0	0
0	0	0	0	0	0	0
0	0	0	0	0	0	0
0	0	0	0	0	0	0
0	0	0	0	0	0	0
0	0	0	0	0	0	0
0	0	0	0	0	0	0
0	0	0	0	0	0	0
0	0	0	0	0	0	0
0	0	0	0	0	0	0
0	0	0	0	0	0	0
0	0	0	0	0	0	0
0	0	0	0	0	0	0
0	0	0	0	0	0	0
0	0	0	0	0	0	0
0	0	0	0	0	0	0
0	0	0	0	0	0	0
0	0	0	0	0	0	0
0	0	0	0	0	0	0
0	0	0	0	0	0	0
0	0	0	0	0	0	0

0	0	0	0	0	0	0
0	0	0	0	0	0	0
0	0	0	0	0	0	0
0	0	0	0	0	0	0
0	0	0	0	0	0	0
0	0	0	0	0	0	0
0	0	0	0	0	0	0
0	0	0	0	0	0	0
0	0	0	0	0	0	0
0	0	0	0	0	0	0
0	0	0	0	0	0	0
0	0	0	0	0	0	0
0	0	0	0	0	0	0
0	0	0	0	0	0	0
0	0	0	0	0	0	0
0	0	0	0	0	0	0
0	0	0	0	0	0	0
0	0	0	0	0	0	0
0	0	0	0	0	0	0
0	0	0	0	0	0	0
0	0	0	0	0	0	0
0	0	0	0	0	0	0
0	0	0	0	0	0	0
0	0	0	0	0	0	0
0	0	0	0	0	0	0
0	0	0	0	0	0	0
0	0	0	0	0	0	0
0	0	0	0	0	0	0
0	0	0	0	0	0	0
0	0	0	0	0	0	0
0	0	0	0	0	0	0
0	0	0	0	0	0	0
0	0	0	0	0	0	0
0	0	0	0	0	0	0
0	0	0	0	0	0	0
0	0	0	0	0	0	0
0	0	0	0	0	0	0
0	0	0	0	0	0	0
115	0	0	0	0	0	0
0	0	0	0	0	0	0
0	0	0	0	0	0	0
0	0	0	0	0	0	0
0	0	0	0	0	0	0

0	0	0	0	0	0	0
0	0	0	0	0	0	0
0	0	0	0	0	0	0
0	0	0	0	0	0	0
0	0	0	0	0	0	0
0	0	0	0	0	0	0
0	0	0	0	0	0	0
0	0	0	0	0	0	0
0	0	0	0	0	0	0
0	0	0	0	0	0	0
0	0	0	0	0	0	0
0	0	0	0	0	0	0
0	0	0	0	0	0	0
0	0	0	0	0	0	0
0	0	0	0	0	0	0
0	0	0	0	0	0	0
0	0	0	0	0	0	0
0	0	0	0	0	0	0
0	0	0	0	0	0	0
0	0	0	0	0	0	0
0	0	0	0	0	0	0
0	0	0	0	0	0	0
0	0	0	0	0	0	0
0	0	0	0	0	0	0
0	0	0	0	0	0	0
0	0	0	0	0	0	0
0	0	0	0	0	0	0
0	0	0	0	0	0	0
0	0	0	0	0	0	0
0	0	0	0	0	0	0
0	0	0	0	0	0	0
0	0	0	0	0	0	0
0	0	0	0	0	0	0
0	0	0	0	0	0	0
0	0	0	0	0	0	0
0	0	0	0	0	0	0
0	0	0	0	2	2	1
0	0	0	0	0	0	0
0	0	0	0	0	0	0
0	0	0	0	0	0	0
0	0	0	0	0	0	0
0	0	0	0	0	0	0
0	0	0	0	0	0	0

0	0	0	0	0	0	0
0	0	0	0	0	0	0
0	0	0	0	0	0	0
0	0	0	0	0	0	0
0	0	0	0	0	0	0
0	0	0	0	0	0	0
0	0	0	0	0	0	0
0	0	0	0	0	0	0
0	0	0	0	0	0	0
0	0	0	0	0	0	0
0	0	0	0	0	0	0
0	0	0	0	0	0	0
0	0	0	0	0	0	0
0	0	0	0	0	0	0
0	0	0	0	0	0	0
0	0	0	0	0	0	0
0	0	0	0	0	0	0
0	0	0	0	0	0	0
0	0	0	0	0	0	0
0	0	0	0	0	0	0
0	0	0	0	0	0	0
0	0	0	0	0	0	0
0	0	0	0	0	0	0
0	0	0	0	0	0	0
0	0	0	0	0	0	0
0	0	0	0	0	0	0
0	0	0	0	0	0	0
0	0	0	0	0	0	0
0	0	0	0	0	0	0
0	0	0	0	0	0	0
0	0	0	0	0	0	0
0	0	0	0	0	0	0
0	0	0	0	0	0	0
0	0	0	0	0	0	0
0	0	0	0	0	0	0
0	0	0	0	0	0	0
0	0	0	0	0	0	0
0	0	0	0	0	0	0
0	0	0	0	0	0	0
0	0	0	0	0	0	0
0	0	0	0	0	0	0
0	0	0	0	0	0	0
0	0	0	0	0	0	0

0	0	0	0	0	0	0
0	0	0	0	0	0	0
0	0	0	0	0	0	0
0	0	0	0	0	0	0
0	0	0	0	0	0	0
0	0	0	0	0	0	0
0	0	0	0	0	0	0
0	0	0	0	0	0	0
0	0	0	0	0	0	0
0	0	0	0	0	0	0
0	0	0	0	0	0	0
0	0	0	0	0	0	0
0	0	0	0	0	0	0
0	0	0	0	0	0	0
0	0	0	0	0	0	0
0	0	0	0	0	0	0
0	0	0	0	0	0	0
0	0	0	0	0	0	0
0	0	0	0	0	0	0
0	0	0	0	0	0	0
0	0	0	2	0	0	0
0	0	0	0	0	0	0
0	0	0	0	0	0	0
0	0	0	0	0	0	0
0	0	0	0	0	0	0
0	0	0	0	0	0	0
0	0	0	0	0	0	0
0	0	0	0	0	0	0
0	0	0	0	0	0	0
0	0	0	0	0	0	0
0	0	0	0	0	0	0
0	0	0	0	0	0	0
0	0	0	0	0	0	0
0	0	0	0	0	0	0
0	0	0	0	0	0	0
2	5	1	1	0	0	0
117	5	1	3	2	2	1

A/B/D	B/C/D	B/C/D/E	TOTAL SUBGE	A1	A5	B1
0	0	0	0	0	0	0
0	0	0	0	0	0	0
0	0	0	0	0	0	0
0	0	0	0	0	0	0
0	0	0	0	0	0	0
0	0	0	0	0	0	0
0	0	0	0	0	0	0
0	0	0	0	0	0	0
0	0	0	0	0	0	0
0	0	0	0	0	0	0
0	0	0	0	0	0	0
0	0	0	0	0	0	0
0	0	0	0	0	0	0
0	0	0	0	0	0	0
0	0	0	0	0	0	0
0	0	0	0	0	0	0
0	0	0	0	0	0	0
0	0	0	0	0	0	0
0	0	0	0	0	0	0
0	0	0	0	0	0	0
0	0	0	0	0	0	0
0	0	0	0	0	0	0
0	0	0	0	0	0	0
0	0	0	0	0	0	0
0	0	0	0	0	0	0
0	0	0	0	0	0	0
0	0	0	0	0	0	0
0	0	0	135	0	0	0
0	0	0	0	0	0	0
0	0	0	0	0	0	0
0	0	0	0	0	0	0
0	0	0	0	0	0	0
0	0	0	0	0	0	0
0	0	0	112	0	0	0
0	0	0	0	0	0	0
0	0	0	87	0	0	0
0	0	0	0	0	0	0
0	0	0	0	0	0	0
0	0	0	0	0	0	0
0	0	0	0	0	0	0
0	0	0	0	0	0	0
0	0	0	0	0	0	0

0	0	0	636	0	0	1
0	0	0	0	0	0	0
0	0	0	0	0	0	0
0	0	0	0	0	0	0
0	0	0	0	0	0	0
0	0	0	0	0	0	0
0	0	0	0	0	0	0
0	0	0	0	0	0	0
0	0	0	0	0	0	0
0	0	0	0	0	0	0
0	0	0	0	0	0	0
0	0	0	0	0	0	0
0	0	0	0	0	0	0
0	0	0	0	0	0	0
0	0	0	0	0	0	0
0	0	0	10	7	0	0
0	0	0	0	0	0	0
0	0	0	0	0	0	0
0	0	0	0	0	0	0
0	0	0	0	0	0	0
0	0	0	0	0	0	0
0	0	0	0	0	0	0
0	0	0	242	0	30	8
0	0	0	0	0	0	0
0	0	0	0	0	0	0
0	0	0	0	0	0	0
0	0	0	0	0	0	0
0	0	0	75	0	0	0
0	0	0	209	0	0	0
0	0	0	0	0	0	0
0	0	0	0	0	0	0
0	0	0	0	0	0	0
0	0	0	179	0	0	0
0	0	0	0	0	0	0
0	0	0	125	0	0	0
0	0	0	0	0	0	0
0	0	0	0	0	0	0
0	0	0	0	0	0	0
0	0	0	0	0	0	0
0	0	0	0	0	0	0
0	0	0	0	0	0	0
0	0	0	0	0	0	0
0	0	0	0	0	0	0

0	0	0	0	0	0	0
0	0	0	0	0	0	0
0	0	0	0	0	0	0
0	0	0	0	0	0	0
0	0	0	0	0	0	0
0	0	0	96	0	0	0
0	0	0	1290	0	0	0
0	0	0	0	0	0	0
0	0	0	0	0	0	0
0	0	0	0	0	0	0
0	0	0	0	0	0	0
0	0	0	0	0	0	0
0	0	0	0	0	0	0
0	0	0	0	0	0	0
0	0	0	0	0	0	0
0	0	0	0	0	0	0
0	5	0	0	0	0	0
0	0	0	0	0	0	0
0	0	0	0	0	0	0
0	0	0	0	0	0	0
2	0	0	0	0	0	0
0	0	0	0	0	0	0
0	0	0	0	0	0	0
0	0	0	0	0	0	0
0	0	0	0	0	0	0
0	0	0	0	0	0	0
0	0	0	0	0	0	0
0	0	0	470	0	0	0
0	0	0	0	0	0	0
0	0	0	0	0	0	0
0	0	0	0	0	0	0
0	0	0	0	0	0	0
0	0	0	0	0	0	0
0	0	0	0	0	0	0
0	0	0	0	0	0	0
0	0	0	54	0	0	0
0	7	2	0	0	0	0
0	0	0	0	0	0	0
0	0	0	219	0	0	0
0	0	0	0	0	0	0
0	0	0	0	0	0	0
0	0	0	0	0	0	0
0	0	0	0	0	0	0

0	0	0	0	0	0	0
0	0	0	0	0	0	0
0	0	0	0	0	0	0
0	0	0	0	0	0	0
0	0	0	0	0	0	0
0	0	0	0	0	0	0
0	0	0	0	0	0	0
0	0	0	0	0	0	0
0	0	0	0	0	0	0
0	0	0	45	9	0	0
0	0	0	0	0	0	0
0	0	0	0	0	0	0
0	0	0	0	0	0	0
0	0	0	0	0	0	0
0	0	0	0	0	0	0
0	0	0	93	0	0	0
0	0	0	0	0	0	0
0	0	0	26	0	0	0
0	0	0	0	0	0	0
0	0	0	37	0	0	26
0	0	0	0	0	0	0
0	0	0	0	0	0	0
0	0	0	0	0	0	0
0	0	0	0	0	0	0
0	0	0	0	0	0	0
0	0	0	0	0	0	0
0	0	0	323	0	0	0
0	0	0	0	0	0	0
0	0	0	0	0	0	0
0	0	0	78	0	0	0
0	0	0	86	0	0	0
0	0	0	0	0	0	0
0	0	0	0	0	0	0
0	0	0	0	0	0	0
0	0	0	0	0	0	0
0	0	0	0	0	0	0
0	0	0	0	0	0	0
0	0	0	0	0	0	0
0	0	0	0	0	0	0
0	0	0	0	0	0	0
0	0	0	0	0	0	0
0	0	0	12	0	0	0
0	0	0	0	0	0	0

0	0	0	0	0	0	0
0	0	0	0	0	0	0
0	0	0	45	0	0	0
0	0	0	0	0	0	0
0	0	0	0	0	0	0
0	0	0	0	0	0	0
0	0	0	0	0	0	0
0	0	0	0	0	0	0
0	0	0	0	0	0	0
0	0	0	112	0	0	0
0	0	0	0	0	0	0
0	0	0	0	0	0	0
0	0	0	0	0	0	0
0	0	0	247	0	0	0
0	0	0	0	0	0	0
0	0	0	0	0	0	0
0	0	0	0	0	0	0
0	0	0	0	0	0	0
0	0	0	0	0	0	0
0	0	0	2202	0	0	2
0	0	0	0	0	0	0
0	0	0	0	0	0	0
0	0	0	0	0	0	0
0	0	0	0	0	0	0
0	0	0	0	0	0	0
0	0	0	0	0	0	0
0	0	0	0	0	0	0
0	0	0	0	0	0	0
0	0	0	0	0	0	0
0	0	0	0	0	0	0
0	0	0	0	0	0	0
0	0	0	0	0	0	0
0	0	0	0	0	0	0
0	0	0	0	0	0	0
0	0	0	0	0	0	0
2	0	0	0	0	0	0
4	12	2	7245	16	30	37

B2	B3	B4	B5	B7	B8	C1
0	0	0	0	0	0	0
0	0	0	0	0	0	0
0	0	0	0	0	0	0
0	0	0	0	0	0	0
0	0	0	0	0	0	0
0	0	0	0	0	0	0
0	0	0	0	0	0	0
0	0	0	0	0	0	0
0	0	0	0	0	0	0
0	0	0	0	0	0	0
5	107	1	5	8	0	37
0	0	0	0	0	0	0
0	0	0	0	0	0	0
0	0	0	0	0	0	0
0	0	0	0	0	0	0
0	0	0	0	0	0	0
0	0	0	0	0	0	0
0	0	0	0	0	0	0
0	0	0	0	0	0	0
0	0	0	0	0	0	0
0	0	0	0	0	0	0
0	0	0	0	0	0	0
0	0	0	0	0	0	0
0	0	0	0	0	0	0
0	0	0	0	0	0	0
0	0	0	0	0	0	0
0	0	0	0	0	0	0
12	0	86	0	0	0	37
0	0	0	0	0	0	0
0	0	0	0	0	0	0
0	0	0	0	0	0	0
0	0	0	0	0	0	0
0	0	0	0	0	0	0
1	0	0	90	0	0	20
0	0	0	0	0	0	0
0	0	0	0	0	0	0
0	0	0	0	0	0	0
0	0	0	0	0	0	0
0	0	0	0	0	0	0
0	0	0	0	0	0	0
0	0	0	0	0	0	0
0	0	0	0	0	0	0

118	7	7	0	2	1	394
0	0	0	0	0	0	0
0	0	0	0	0	0	0
0	0	0	0	0	0	0
0	0	0	0	0	0	0
0	0	0	0	0	0	0
0	0	0	0	0	0	0
0	0	0	0	0	0	0
0	0	0	0	0	0	0
0	0	0	0	0	0	0
0	0	0	0	0	0	0
0	0	0	0	0	0	0
0	0	0	0	0	0	0
0	0	0	0	0	0	0
0	0	0	0	0	0	0
0	0	0	0	0	0	0
0	0	0	0	0	0	0
0	0	0	0	0	0	0
0	0	0	0	0	0	0
0	0	0	0	0	0	0
0	0	0	0	0	0	0
0	0	0	0	0	0	0
0	0	0	0	0	21	0
0	0	0	0	0	0	0
0	0	0	0	0	0	0
0	0	0	0	0	0	0
0	0	0	0	0	0	0
37	0	13	0	0	0	0
0	0	0	0	0	0	0
0	0	0	0	0	0	0
0	0	0	0	0	0	0
0	0	0	0	0	0	0
10	0	0	0	0	0	0
0	0	0	0	0	0	0
0	0	0	0	0	0	0
0	0	0	0	0	0	0
0	0	0	0	0	0	0
0	0	0	0	0	0	0
0	0	0	0	0	0	0
0	0	0	0	0	0	0
0	0	0	0	0	0	0
0	0	0	0	0	0	0
0	0	0	0	0	0	0

0	0	0	0	0	0	0
0	0	0	0	0	0	0
0	0	0	0	0	0	0
0	0	0	0	0	0	0
0	0	0	0	0	0	0
35	0	0	0	0	0	4
182	0	0	0	0	0	0
0	0	0	0	0	0	0
0	0	0	0	0	0	0
0	0	0	0	0	0	0
0	0	0	0	0	0	0
0	0	0	0	0	0	0
0	0	0	0	0	0	0
0	0	0	0	0	0	0
0	0	0	0	0	0	0
0	0	0	0	0	0	0
0	0	0	0	0	0	0
0	0	0	0	0	0	0
0	0	0	0	0	0	0
0	0	0	0	0	0	0
0	0	0	0	0	0	0
0	0	0	0	0	0	0
0	0	0	0	0	0	0
0	0	0	0	0	0	0
0	0	0	0	0	0	0
0	0	0	0	0	0	0
0	0	0	0	0	0	0
63	0	0	0	0	0	0
0	0	0	0	0	0	0
0	0	0	0	0	0	0
0	0	0	0	0	0	0
0	0	0	0	0	0	0
0	0	0	0	0	0	0
0	0	0	0	0	0	0
0	0	0	0	0	0	0
0	0	0	0	0	0	0
0	0	0	0	0	0	0
0	0	0	0	0	0	0
8	9	8	0	0	0	192
0	0	0	0	0	0	0
0	0	0	0	0	0	0
0	0	0	0	0	0	0
0	0	0	0	0	0	0

0	0	0	0	0	0	0
0	0	0	0	0	0	0
0	0	0	0	0	0	0
0	0	0	0	0	0	0
0	0	0	0	0	0	0
0	0	0	0	0	0	0
0	0	0	0	0	0	0
0	0	0	0	0	0	0
0	0	0	0	0	0	0
0	0	0	0	0	0	21
0	0	0	0	0	0	0
0	0	0	0	0	0	0
0	0	0	0	0	0	0
0	0	0	0	0	0	0
0	0	0	0	0	0	0
0	0	0	0	0	0	0
0	0	0	0	0	0	0
0	0	0	0	0	0	0
0	0	0	0	0	0	0
0	0	0	0	0	0	0
0	0	0	0	0	0	0
0	0	0	0	0	0	0
0	0	0	0	0	0	0
0	0	0	0	0	0	0
0	0	0	0	0	0	0
0	0	0	0	0	0	0
162	2	2	0	0	0	27
			0	0	0	0
0	0	0	0	0	0	0
0	0	0	0	0	0	0
0	0	0	0	0	0	0
0	0	0	0	0	0	0
0	0	0	0	0	0	0
0	0	0	0	0	0	0
0	0	0	0	0	0	0
0	0	0	0	0	0	0
0	0	0	0	0	0	0
0	0	0	0	0	0	0
0	0	0	0	0	0	0
0	0	0	0	0	0	0
0	0	0	0	0	0	0
0	0	4	0	0	0	12
0	0	0	0	0	0	0

0	0	0	0	0	0	0
0	35	0	0	0	0	6
0	0	0	0	0	0	10
0	0	0	0	0	0	0
0	0	0	0	0	0	0
0	0	0	0	0	0	0
0	0	0	0	0	0	0
0	0	0	0	0	0	0
0	0	0	0	0	0	0
85	0	0	0	0	0	0
0	0	0	0	0	0	0
0	0	0	0	0	0	0
0	0	0	0	0	0	0
92	0	0	0	0	0	30
0	0	0	0	0	0	0
0	0	0	0	0	0	0
0	0	0	0	0	0	0
0	0	0	0	0	0	0
0	0	0	0	0	0	0
601	6	2	0	0	0	236
0	0	0	0	0	0	0
0	0	0	0	0	0	0
0	0	0	0	0	0	0
0	0	0	0	0	0	0
0	0	0	0	0	0	0
0	0	0	0	0	0	0
0	0	0	0	0	0	0
0	0	0	0	0	0	0
0	0	0	0	0	0	0
0	0	0	0	0	0	0
0	0	0	0	0	0	0
0	0	0	0	0	0	0
0	0	0	0	0	0	0
0	0	0	0	0	0	0
0	0	0	0	0	0	0
0	0	0	0	0	0	0
1411	166	123	95	10	22	1026

C2	C3	C4	C5	C6	C7	C8
0	0	0	0	0	0	0
0	0	0	0	0	0	0
0	0	0	0	0	0	0
0	0	0	0	0	0	0
0	0	0	0	0	0	0
0	0	0	0	0	0	0
0	0	0	0	0	0	0
0	0	0	0	0	0	0
0	0	0	0	0	0	0
0	0	0	0	0	0	0
1	7	0	0	0	0	0
0	0	0	0	0	0	0
0	0	0	0	0	0	0
0	0	0	0	0	0	0
0	0	0	0	0	0	0
0	0	0	0	0	0	0
0	0	0	0	0	0	0
0	0	0	0	0	0	0
0	0	0	0	0	0	0
0	0	0	0	0	0	0
0	0	0	0	0	0	0
0	0	0	0	0	0	0
0	0	0	0	0	0	0
0	0	0	0	0	0	0
0	0	0	0	0	0	0
0	0	0	0	0	0	0
0	0	0	0	0	0	0
0	0	0	0	0	0	0
0	0	0	0	0	0	0
0	0	0	0	0	0	0
0	0	0	0	0	0	0
0	0	0	0	0	0	0
0	0	0	0	0	0	0
0	0	0	0	0	0	0
0	0	0	0	0	0	0
0	0	0	0	0	0	0
0	0	0	0	0	0	0
0	0	0	0	0	0	0
0	0	0	0	0	0	0
0	0	0	0	0	0	0
0	0	0	0	0	0	0
0	0	0	0	0	0	0

60	0	0	17	0	0	24
0	0	0	0	0	0	0
0	0	0	0	0	0	0
0	0	0	0	0	0	0
0	0	0	0	0	0	0
0	0	0	0	0	0	0
0	0	0	0	0	0	0
0	0	0	0	0	0	0
0	0	0	0	0	0	0
0	0	0	0	0	0	0
0	0	0	0	0	0	0
0	0	0	0	0	0	0
0	0	0	0	0	0	0
0	0	0	0	0	0	0
0	0	0	0	0	0	0
0	0	0	0	0	0	0
0	0	0	0	0	0	0
0	0	0	0	0	0	0
0	0	0	0	0	0	0
0	0	0	0	0	0	0
0	0	0	0	0	0	0
0	0	0	0	0	0	0
176	0	0	7	0	0	0
0	0	0	0	0	0	0
0	0	0	0	0	0	0
0	0	0	0	0	0	0
0	0	0	0	0	0	0
22	0	3	0	0	0	0
209	0	0	0	0	0	0
0	0	0	0	0	0	0
0	0	0	0	0	0	0
0	0	0	0	0	0	0
169	0	0	0	0	0	0
0	0	0	0	0	0	0
0	0	0	0	0	0	0
0	0	0	0	0	0	0
0	0	0	0	0	0	0
0	0	0	0	0	0	0
0	0	0	0	0	0	0
0	0	0	0	0	0	0
0	0	0	0	0	0	0
0	0	0	0	0	0	0
0	0	0	0	0	0	0

0	0	0	0	0	0	0
0	0	0	0	0	0	0
0	0	0	0	0	0	0
0	0	0	0	0	0	0
0	0	0	0	0	0	0
57	0	0	0	0	0	0
1096	0	0	0	0	0	0
0	0	0	0	0	0	0
0	0	0	0	0	0	0
0	0	0	0	0	0	0
0	0	0	0	0	0	0
0	0	0	0	0	0	0
0	0	0	0	0	0	0
0	0	0	0	0	0	0
0	0	0	0	0	0	0
0	0	0	0	0	0	0
0	0	0	0	0	0	0
0	0	0	0	0	0	0
0	0	0	0	0	0	0
0	0	0	0	0	0	0
0	0	0	0	0	0	0
0	0	0	0	0	0	0
0	0	0	0	0	0	0
0	0	0	0	0	0	0
0	0	0	0	0	0	0
0	0	0	0	0	0	0
0	0	0	0	0	0	0
407	0	0	0	0	0	0
0	0	0	0	0	0	0
0	0	0	0	0	0	0
0	0	0	0	0	0	0
0	0	0	0	0	0	0
0	0	0	0	0	0	0
0	0	0	0	0	0	0
0	0	0	0	0	0	0
0	0	0	0	0	0	0
0	0	0	0	0	0	0
0	0	0	0	0	0	0
0	0	0	2	0	0	0
0	0	0	0	0	0	0
0	0	0	0	0	0	0
0	0	0	0	0	0	0
0	0	0	0	0	0	0

0	0	0	0	0	0	0
0	0	0	0	0	0	0
0	0	0	0	0	0	0
0	0	0	0	0	0	0
0	0	0	0	0	0	0
0	0	0	0	0	0	0
0	0	0	0	0	0	0
0	0	0	0	0	0	0
0	0	0	0	0	0	0
0	0	0	0	0	0	0
0	0	0	0	0	0	0
0	0	0	0	0	0	0
0	0	0	0	0	0	0
0	0	0	0	0	0	0
0	0	0	0	0	0	0
0	0	0	0	0	0	0
0	0	0	0	0	0	0
0	0	0	0	0	0	0
0	0	0	0	0	0	0
0	0	0	11	0	0	0
0	0	0	0	0	0	0
0	0	0	0	0	0	0
0	0	0	0	0	0	0
0	0	0	0	0	0	0
0	0	0	0	0	0	0
0	0	0	0	0	0	0
93	3	6	4	2	0	0
0	0	0	0	0	0	0
0	0	0	0	0	0	0
0	0	0	0	0	0	0
0	0	0	0	0	0	0
0	0	0	0	0	0	0
0	0	0	0	0	0	0
0	0					
0	0	0	0	0	0	0
0	0	0	0	0	0	0
0	0	0	0	0	0	0
0	0	0	0	0	0	0
0	0	0	0	0	0	0
0	0	0	0	0	0	0
0	0	0	0	0	0	0
0	0	0	0	0	0	0
0	0	0	0	0	0	0

0	0	0	0	0	0	0
0	0	0	0	0	0	0
28	0	0	3	0	4	0
0	0	0	0	0	0	0
0	0	0	0	0	0	0
0	0	0	0	0	0	0
0	0	0	0	0	0	0
0	0	0	0	0	0	0
0	0	0	0	0	0	0
27	0	0	0	0	0	0
0	0	0	0	0	0	0
0	0	0	0	0	0	0
0	0	0	0	0	0	0
125	0	0	0	0	0	0
0	0	0	0	0	0	0
0	0	0	0	0	0	0
0	0	0	0	0	0	0
0	0	0	0	0	0	0
0	0	0	0	0	0	0
1277	4	2	15	0	0	0
0	0	0	0	0	0	0
0	0	0	0	0	0	0
0	0	0	0	0	0	0
0	0	0	0	0	0	0
0	0	0	0	0	0	0
0	0	0	0	0	0	0
0	0	0	0	0	0	0
0	0	0	0	0	0	0
0	0	0	0	0	0	0
0	0	0	0	0	0	0
0	0	0	0	0	0	0
0	0	0	0	0	0	0
0	0	0	0	0	0	0
0	0	0	0	0	0	0
0	0	0	0	0	0	0
0	0	0	0	0	0	0
3747	14	11	59	2	4	24

C9	C10	C17	D1	D2	D3	D4
0	0	0	0	0	0	0
0	0	0	0	0	0	0
0	0	0	0	0	0	0
0	0	0	0	0	0	0
0	0	0	0	0	0	0
0	0	0	0	0	0	0
0	0	0	0	0	0	0
0	0	0	0	0	0	0
0	0	0	0	0	0	0
0	0	0	0	0	0	0
0	0	0	0	0	0	0
0	0	0	0	0	0	0
0	0	0	0	0	0	0
0	0	0	0	0	0	0
0	0	0	0	0	0	0
0	0	0	0	0	0	0
0	0	0	0	0	0	0
0	0	0	0	0	0	0
0	0	0	0	0	0	0
0	0	0	0	0	0	0
0	0	0	0	0	0	0
0	0	0	0	0	0	0
0	0	0	0	0	0	0
0	0	0	0	0	0	0
0	0	0	0	0	0	0
0	0	0	0	0	0	0
0	0	0	0	0	0	0
0	0	0	0	0	0	0
0	0	0	0	0	0	0
0	0	0	0	0	0	0
0	0	0	0	0	0	0
0	0	0	0	0	0	0
0	0	0	0	0	0	0
0	0	0	0	0	0	0
0	0	0	0	0	0	0
0	0	0	87	0	0	0
0	0	0	0	0	0	0
0	0	0	0	0	0	0
0	0	0	0	0	0	0
0	0	0	0	0	0	0
0	0	0	0	0	0	0
0	0	0	0	0	0	0

	1	1	0	0	0	0
0	0	0	0	0	0	0
0	0	0	0	0	0	0
0	0	0	0	0	0	0
0	0	0	0	0	0	0
0	0	0	0	0	0	0
0	0	0	0	0	0	0
0	0	0	0	0	0	0
0	0	0	0	0	0	0
0	0	0	0	0	0	0
0	0	0	0	0	0	0
0	0	0	0	0	0	0
0	0	0	0	0	0	0
0	0	0	0	0	0	0
0	0	0	0	0	0	0
0	0	0	1	2	0	0
0	0	0	0	0	0	0
0	0	0	0	0	0	0
0	0	0	0	0	0	0
0	0	0	0	0	0	0
0	0	0	0	0	0	0
0	0	0	0	0	0	0
0	0	0	0	0	0	0
0	0	0	0	0	0	0
0	0	0	0	0	0	0
0	0	0	0	0	0	0
0	0	0	0	0	0	0
0	0	0	0	0	0	0
0	0	0	0	0	0	0
0	0	0	0	0	0	0
0	0	0	0	0	0	0
0	0	0	0	0	0	0
0	0	0	0	0	0	0
0	0	0	0	0	0	0
0	0	0	2	122	0	0
0	0	0	0	0	0	0
0	0	0	0	0	0	0
0	0	0	0	0	0	0
0	0	0	0	0	0	0
0	0	0	0	0	0	0
0	0	0	0	0	0	0
0	0	0	0	0	0	0
0	0	0	0	0	0	0

0	0	0	0	0	0	0
0	0	0	0	0	0	0
0	0	0	0	0	0	0
0	0	0	0	0	0	0
0	0	0	0	0	0	0
0	0	0	0	0	0	0
0	0	0	12	0	0	0
0	0	0	0	0	0	0
0	0	0	0	0	0	0
0	0	0	0	0	0	0
0	0	0	0	0	0	0
0	0	0	0	0	0	0
0	0	0	0	0	0	0
0	0	0	0	0	0	0
0	0	0	0	0	0	0
0	0	0	0	0	0	0
0	0	0	0	0	0	0
0	0	0	0	0	0	0
0	0	0	0	0	0	0
0	0	0	0	0	0	0
0	0	0	0	0	0	0
0	0	0	0	0	0	0
0	0	0	0	0	0	0
0	0	0	0	0	0	0
0	0	0	0	0	0	0
0	0	0	0	0	0	0
0	0	0	0	0	0	0
0	0	0	0	0	0	0
0	0	0	0	0	0	0
0	0	0	0	0	0	0
0	0	0	0	0	0	0
0	0	0	0	0	0	0
0	0	0	0	0	0	0
0	0	0	0	0	0	0
0	0	0	0	0	0	0
0	0	0	7	44	0	0
0	0	0	0	0	0	0
0	0	0	0	0	0	0
0	0	0	0	0	0	0
0	0	0	0	0	0	0
0	0	0	0	0	0	0
0	0	0	0	0	0	0
0	0	0	0	0	0	0

0	0	0	0	0	0	0
0	0	0	0	0	0	0
0	0	0	0	0	0	0
0	0	0	0	0	0	0
0	0	0	0	0	0	0
0	0	0	0	0	0	0
0	0	0	0	0	0	0
0	0	0	0	0	0	0
0	0	0	0	0	0	0
0	0	0	0	15	0	0
0	0	0	0	0	0	0
0	0	0	0	0	0	0
0	0	0	0	0	0	0
0	0	0	0	0	0	0
0	0	0	0	0	0	0
0	0	0	76	9	8	0
0	0	0	0	0	0	0
0	0	0	24	2	0	0
0	0	0	0	0	0	0
0	0	0	0	0	0	0
0	0	0	0	0	0	0
0	0	0	0	0	0	0
0	0	0	0	0	0	0
0	0	0	0	0	0	0
0	0	0	0	0	0	0
0	0	0	0	0	0	0
0	0	0	19	0	2	1
0	0	0	0	0	0	0
0	0	0	0	0	0	0
0	0	0	3	67	1	0
0	0	0	4	74	2	0
0	0	0	0	0	0	0
0	0	0	0	0	0	0
0	0	0	0	0	0	0
0	0	0	0	0	0	0
0	0	0	0	0	0	0
0	0	0	0	0	0	0
0	0	0	0	0	0	0
0	0	0	0	0	0	0
0	0	0	0	0	0	0
0	0	0	0	0	0	0
0	0	0	0	0	0	0

0	0	0	0	0	0	0
0	0	0	0	0	0	0
0	0	0	0	0	0	0
0	0	0	0	0	0	0
0	0	0	0	0	0	0
0	0	0	0	0	0	0
0	0	0	0	0	0	0
0	0	0	0	0	0	0
0	0	0	0	0	0	0
0	0	0	0	0	0	0
0	0	0	0	0	0	0
0	0	0	0	0	0	0
0	0	0	0	0	0	0
0	0	0	0	0	0	0
0	0	0	0	0	0	0
0	0	0	0	0	0	0
0	0	0	0	0	0	0
0	0	0	0	0	0	0
0	0	0	0	0	0	0
31	0	0	21	5	0	0
0	0	0	0	0	0	0
0	0	0	0	0	0	0
0	0	0	0	0	0	0
0	0	0	0	0	0	0
0	0	0	0	0	0	0
0	0	0	0	0	0	0
0	0	0	0	0	0	0
0	0	0	0	0	0	0
0	0	0	0	0	0	0
0	0	0	0	0	0	0
0	0	0	0	0	0	0
0	0	0	0	0	0	0
0	0	0	0	0	0	0
0	0	0	0	0	0	0
0	0	0	0	0	0	0
0	0	0	0	0	0	0
31	1	1	256	340	13	1

D5	D6	D/Ddel
0	0	0
0	0	0
0	0	0
0	0	0
0	0	0
0	0	0
0	0	0
0	0	0
0	0	0
0	0	0
0	0	0
0	0	0
0	0	0
0	0	0
0	0	0
0	0	0
0	0	0
0	0	0
0	0	0
0	0	0
0	0	0
0	0	0
0	0	0
0	0	0
0	0	0
0	0	0
0	0	0
0	0	0
0	0	0
0	0	0
0	0	0
0	0	0
0	0	0
0	0	0
0	0	0
0	0	0
0	0	0
0	0	0
0	0	0
0	0	0
0	0	0
0	0	0

0	0	0
0	0	0
0	0	0
0	0	0
0	0	0
0	0	0
0	0	0
0	0	0
0	0	0
0	0	0
0	0	0
0	0	0
0	0	0
0	0	0
0	0	0
2	0	0
0	0	0
0	0	0
0	0	0
0	0	0
0	0	0
0	0	0
0	0	0
0	0	0
0	0	0
0	0	0
0	0	0
0	0	0
0	0	0
0	0	0
0	0	0
0	0	0
0	0	0
0	0	0
0	0	1
0	0	0
0	0	0
0	0	0
0	0	0
0	0	0
0	0	0
0	0	0
0	0	0

0	0	0
0	0	0
0	0	0
0	0	0
0	0	0
0	0	0
0	0	0
0	0	0
0	0	0
0	0	0
0	0	0
0	0	0
0	0	0
0	0	0
0	0	0
0	0	0
0	0	0
0	0	0
0	0	0
0	0	0
0	0	0
0	0	0
0	0	0
0	0	0
0	0	0
0	0	0
0	0	0
0	0	0
0	0	0
0	0	0
0	0	0
0	0	0
0	0	0
0	0	0
0	0	0
0	3	0
0	0	0
0	0	0
0	0	0
0	0	0
0	0	0
0	0	0
0	0	0

0	0	0
0	0	0
0	0	0
0	0	0
0	0	0
0	0	0
0	0	0
0	0	0
0	0	0
0	0	0
0	0	0
0	0	0
0	0	0
0	0	0
0	0	0
0	0	0
0	0	0
0	0	0
0	0	0
0	0	0
0	0	0
0	0	0
0	0	0
0	0	0
0	0	0
0	0	0
0	0	0
0	0	0
0	0	0
0	0	7
0	0	0
0	0	0
0	0	0
0	0	0
0	0	0
0	0	0
0	0	0
0	0	0
0	0	0
0	0	0
0	0	0
0	0	0

0	0	0
0	0	0
0	0	0
0	0	0
0	0	0
0	0	0
0	0	0
0	0	0
0	0	0
0	0	0
0	0	0
0	0	0
0	0	0
0	0	0
0	0	0
0	0	0
0	0	0
0	0	0
0	0	0
0	0	0
0	0	0
0	0	0
0	0	0
0	0	0
0	0	0
0	0	0
0	0	0
0	0	0
0	0	0
0	0	0
0	0	0
0	0	0
0	0	0
0	0	0
0	0	0
0	0	0
2	3	8

PUBMED

(tajikistan[Title/Abstract]) OR (uzbekistan[Title/Abstract])) OR (kazakhstan[Title/Abstract])) OR (turkmenistan[Title/Abstract])) OR (kyrgyzstan[Title/Abstract])) OR (georgia[Title/Abstract])) OR (armenia[Title/Abstract])) OR (azerbaijan[Title/Abstract])) OR (turkey[Title/Abstract])) OR (cyprus[Title/Abstract])) OR (syria[Title/Abstract])) OR (lebanon[Title/Abstract])) OR (israel[Title/Abstract])) OR (jordan[Title/Abstract])) OR (iraq[Title/Abstract])) OR (oman[Title/Abstract])) OR (yemen[Title/Abstract])) OR (kuwait[Title/Abstract])) OR (bahrain[Title/Abstract])) OR (qatar[Title/Abstract])) OR (brunei[Title/Abstract])) OR (cambodia[Title/Abstract])) OR (indonesia[Title/Abstract])) OR (laos[Title/Abstract])) OR (malaysia[Title/Abstract])) OR (myanmar[Title/Abstract])) OR (philippine[Title/Abstract])) OR (singapore[Title/Abstract])) OR (sri lanka[Title/Abstract])) OR (thailand[Title/Abstract])) OR (timor-leste[Title/Abstract])) OR (vietnam[Title/Abstract])) OR (bangladesh[Title/Abstract])) OR (india[Title/Abstract])) OR (afghanistan[Title/Abstract])) OR (pakistan[Title/Abstract])) OR (bhutan[Title/Abstract])) OR (nepal[Title/Abstract])) OR (maldives[Title/Abstract])) OR (iran[Title/Abstract])) OR (china[Title/Abstract])) OR (mongolia[Title/Abstract])) OR (north korea[Title/Abstract])) OR (south korea[Title/Abstract])) OR (japan[Title/Abstract])) OR (hong kong[Title/Abstract])) OR (taiwan[Title/Abstract])) OR (macau[Title/Abstract]))) OR (timor[Title/Abstract])) AND ("genot*"[Title/Abstract])) AND ((HBV[Title/Abstract]) OR ("Hepatitis B"[Title/Abstract]))

SCOPUS

Scopus

1. (genotype OR genotypes) (HBV OR "Hepatitis B")(tajikistan OR uzbekistan OR kazakhstan OR turkmenistan)
2. (genotype OR genotypes) (HBV OR "Hepatitis B") (kyrgyzstan OR georgia OR armenia OR azerbaijan)
3. (genotype OR genotypes) (HBV OR "Hepatitis B") (turkey OR cyprus OR syria OR lebanon)
4. (genotype OR genotypes)(HBV OR "Hepatitis B") (israel OR jordan OR iraq OR oman)
5. (genotype OR genotypes) (HBV OR "Hepatitis B") (yemen OR kuwait OR bahrain OR qatar)
6. (genotype OR genotypes) (HBV OR "Hepatitis B") (brunei OR cambodia OR indonesia OR laos)
7. (genotype OR genotypes) (HBV OR "Hepatitis B") (malaysia OR myanmar OR philippine OR singapore)
8. (genotype OR genotypes) (HBV OR "Hepatitis B") (sri lanka OR thailand OR timor-leste OR vietnam)
9. (genotype OR genotypes) (HBV OR "Hepatitis B") (bangladesh OR india OR afghanistan OR pakistan)
10. (genotype OR genotypes) (HBV OR "Hepatitis B") (bhutan OR nepal OR maldives OR iran)
11. (genotype OR genotypes) (HBV OR "Hepatitis B") (china OR mongolia OR “north korea” OR “south korea”)
12. (genotype OR genotypes) (HBV OR "Hepatitis B") (japan OR “hong kong” OR taiwan OR macau)
13. (genotype OR genotypes) (HBV OR "Hepatitis B") (timor)

ScienceDirect

1. (genotype OR genotypes) (HBV OR "Hepatitis B")(tajikistan OR uzbekistan OR kazakhstan OR turkmenistan)
2. (genotype OR genotypes) (HBV OR "Hepatitis B") (kyrgyzstan OR georgia OR armenia OR azerbaijan)-1
3. (genotype OR genotypes) (HBV OR "Hepatitis B") (turkey OR cyprus OR syria OR lebanon)-24
4. (genotype OR genotypes)(HBV OR "Hepatitis B") (israel OR jordan OR iraq OR oman)-6
5. (genotype OR genotypes) (HBV OR "Hepatitis B") (yemen OR kuwait OR bahrain OR qatar)-1
6. (genotype OR genotypes) (HBV OR "Hepatitis B") (brunei OR cambodia OR indonesia OR laos)-10
7. (genotype OR genotypes) (HBV OR "Hepatitis B") (malaysia OR myanmar OR philippine OR singapore) - 3
8. (genotype OR genotypes) (HBV OR "Hepatitis B") (sri lanka OR thailand OR timor-leste OR vietnam)-13
9. (genotype OR genotypes) (HBV OR "Hepatitis B") (bangladesh OR india OR afghanistan OR pakistan)-49
10. (genotype OR genotypes) (HBV OR "Hepatitis B") (bhutan OR nepal OR maldives OR iran)-22
11. (genotype OR genotypes) (HBV OR "Hepatitis B") (china OR mongolia OR “north korea” OR “south korea”)-144
12. (genotype OR genotypes) (HBV OR "Hepatitis B") (japan OR “hong kong” OR taiwan OR macau)-106
13. (genotype OR genotypes) (HBV OR "Hepatitis B") (timor)-237

Google Scholar

GROUP A

allintitle: hepatitis b genotype tajikistan OR uzbekistan OR kazakhstan OR turkmenistan OR kyrgyzstan OR georgia OR armenia OR azerbaijan OR turkey OR cyprus OR syria OR lebanon OR israel OR jordan OR iraq OR oman OR yemen OR kuwait OR bahrain OR Qatar

GROUP B

allintitle: hepatitis b genotype myanmar OR Philippine OR singapore OR sri-lanka OR thailand OR timor-leste OR vietnam OR bangladesh OR india OR afghanistan OR pakistan OR bhutan OR nepal OR maldives OR iran "Hepatitis B" -"hepatitis c"

GROUP C

allintitle: hepatitis B genotype china OR mongolia OR "north korea" OR "south korea" OR japan OR "hong kong" OR taiwan OR macau OR timor OR brunei OR cambodia OR indonesia OR laos OR Malaysia "hepatitis b"

QUALITY OF INCLUDED STUDIES BY JBI CRITICAL APPRAISAL CHECKLIST FOR STUDIES REPORTING PREVALENCE DATA

S/N	Name of authors and year of publication		JBI checklist*									Total
1	Abbas et al	2009	1	2	3	4	5	6	7	8	9	
2	Abbasian et al	2021	Yes	No	Yes	Yes	Yes	Yes	Yes	Yes	Yes	16
3	Abdulkadirova et al	2020	Yes	No	Yes	Yes	Yes	Yes	Yes	Yes	Yes	16
4	Aftab et al	2018	Yes	No	Yes	Yes	Yes	Yes	Yes	Yes	Yes	16
5	Aghakhani et al	2009	Yes	No	Yes	Yes	Yes	Yes	Yes	Yes	Yes	16
6	Alam. et al	2007	Yes	No	Yes	Yes	Yes	Yes	Yes	Yes	Yes	16
7	Alam et al	2007	Yes	No	Yes	Yes	Yes	Yes	Yes	Yes	Yes	16
8	Alavian et al	2006	Yes	No	Yes	Yes	Yes	Yes	Yes	Yes	Yes	16
9	Anvari et al	2016	Yes	No	Yes	Yes	Yes	Yes	Yes	Yes	Yes	16
10	Andi. et al	2009	Yes	No	Yes	Yes	Yes	Yes	Yes	Yes	Yes	16
11	Andi et al	2009	Yes	No	Yes	Yes	Yes	Yes	Yes	Yes	Yes	16
12	Anis et al	2008	Yes	No	Yes	Yes	Yes	Yes	Yes	Yes	Yes	16
13	Anvari et al	2013	Yes	No	Yes	Yes	Yes	Yes	Yes	Yes	Yes	16
14	Aono et al	2007	Yes	No	Yes	Yes	Yes	Yes	Yes	Yes	Yes	16
15	Arabadadi et al	2011	Yes	No	Yes	Yes	Yes	Yes	Yes	Yes	Yes	16
16	Arifur et al	2016	Yes	No	Yes	Yes	Yes	Yes	Yes	Yes	Yes	16
17	Arup et al	2006	Yes	No	Yes	Yes	Yes	Yes	Yes	Yes	Yes	16
18	Ashrafali et al	2014	Yes	No	Yes	Yes	Yes	Yes	Yes	Yes	Yes	16
19	Atalay et al	2011	Yes	No	Yes	Yes	Yes	Yes	Yes	Yes	Yes	16
20	Attaran et al	2017	Yes	No	Yes	Yes	Yes	Yes	Yes	Yes	Yes	16
21	Avik. et al	2013	Yes	No	Yes	Yes	Yes	Yes	Yes	Yes	Yes	16
22	Avik et al	2009	Yes	No	Yes	Yes	Yes	Yes	Yes	Yes	Yes	16
23	Babana et al	2017	Yes	No	Yes	Yes	Yes	Yes	Yes	Yes	Yes	16
24	Bhagirathi et al	2014	Yes	No	Yes	Yes	Yes	Yes	Yes	Yes	Yes	16
25	Borkakoty et al	2008	Yes	No	Yes	Yes	Yes	Yes	Yes	Yes	Yes	16
26	Bozdayo et al	2003	Yes	No	Yes	Yes	Yes	Yes	Yes	Yes	Yes	16
27	Brij et al	2018	Yes	Yes	Yes	Yes	Yes	Yes	Yes	Yes	Yes	18
28	Bui et al	2017	Yes	Yes	Yes	Yes	Yes	Yes	Yes	Yes	Yes	18
29	Chamni et al	2014	Yes	No	Yes	Yes	Yes	Yes	Yes	Yes	Yes	16
30	Chan et al	2002	Yes	No	Yes	Yes	Yes	Yes	Yes	Yes	Yes	16
31	Chandana et al	2011	Yes	No	Yes	Yes	Yes	Yes	Yes	Yes	Yes	16
32	Choudhardy et al	2008	Yes	No	Yes	Yes	Yes	Yes	Yes	Yes	Yes	16
33	Ciftci et al	2012	Yes	No	Yes	Yes	Yes	Yes	Yes	Yes	Yes	16
34	Cuong et al	2011	Yes	No	Yes	Yes	Yes	Yes	Yes	Yes	Yes	16
35	D-D Li et al	2010	Yes	No	Yes	Yes	Yes	Yes	Yes	Yes	Yes	16
36	Davod et al	2019	Yes	No	Yes	Yes	Yes	Yes	Yes	Yes	Yes	16
37	Debraj et al	2014	Yes	Yes	Yes	Yes	Yes	Yes	Yes	Yes	Yes	18
38	Ding. et al	2004	Yes	No	Yes	Yes	Yes	Yes	Yes	Yes	Yes	16
39	Ding.. et al	2013	Yes	Yes	Yes	Yes	Yes	Yes	Yes	Yes	Yes	18
40	Ding et al.	2002	Yes	No	Yes	Yes	Yes	Yes	Yes	Yes	Yes	16
41	Ding et al	2001	Yes	Yes	Yes	Yes	Yes	Yes	Yes	Yes	Yes	18
42	Dong. et al	2009	Yes	No	Yes	Yes	Yes	Yes	Yes	Yes	Yes	16
43	Dong.. et al	2021	Yes	No	Yes	Yes	Yes	Yes	Yes	Yes	Yes	16
44	Dong et al	2009	Yes	No	Yes	Yes	Yes	Yes	Yes	Yes	Yes	16
45	Du et al	2006	Yes	Yes	Yes	Yes	Yes	Yes	Yes	Yes	Yes	18
46	Edward et al	2005	Yes	No	Yes	Yes	Yes	Yes	Yes	Yes	Yes	16
47	Emekdas et al	2012	Yes	Yes	Yes	Yes	Yes	Yes	Yes	Yes	Yes	18
48	Etsuro et al	2001	Yes	No	Yes	Yes	Yes	Yes	Yes	Yes	Yes	16
49	Fang et al	2004	Yes	Yes	Yes	Yes	Yes	Yes	Yes	Yes	Yes	18
50	Farah et al	2014	Yes	No	Yes	Yes	Yes	Yes	Yes	Yes	Yes	16
51	Farooq et al	2021	Yes	No	Yes	Yes	Yes	Yes	Yes	Yes	Yes	16
52	Fei et al	2009	Yes	No	Yes	Yes	Yes	Yes	Yes	Yes	Yes	16
53	Fujie et al	2001	Yes	No	Yes	Yes	Yes	Yes	Yes	Yes	Yes	16
54	Fujiko et al	2015	Yes	Yes	Yes	Yes	Yes	Yes	Yes	Yes	Yes	18
55	Fuminaka et al	2002	Yes	Yes	Yes	Yes	Yes	Yes	Yes	Yes	Yes	18
56	Furusyo et al	2004	Yes	No	Yes	Yes	Yes	Yes	Yes	Yes	Yes	16
57	Ghani et al	2011	Yes	No	Yes	Yes	Yes	Yes	Yes	Yes	Yes	16
58	Gollapudi et al	2008	Yes	No	Yes	Yes	Yes	Yes	Yes	Yes	Yes	16
59	Gu et al	2004	Yes	No	Yes	Yes	Yes	Yes	Yes	Yes	Yes	16
60	Haimanti et al	2014	Yes	No	Yes	Yes	Yes	Yes	Yes	Yes	Yes	16
61	Haixia et al	2013	Yes	No	Yes	Yes	Yes	Yes	Yes	Yes	Yes	16

62	Hakim et al	2016	Yes	No	Yes	Yes	Yes	Yes	Yes	Yes	Yes	16
63	Han et al	2009	Yes	No	Yes	Yes	Yes	Yes	Yes	Yes	Yes	16
64	Hani et al	2008	Yes	No	Yes	Yes	Yes	Yes	Yes	Yes	Yes	16
65	Hayashi et al	2007	Yes	No	Yes	Yes	Yes	Yes	Yes	Yes	Yes	16
66	Hideaki et al	2002	Yes	No	Yes	Yes	Yes	Yes	Yes	Yes	Yes	16
67	Hong mei et al	2015	Yes	No	Yes	Yes	Yes	Yes	Yes	Yes	Yes	16
68	Hooi et al	2005	Yes	No	Yes	Yes	Yes	Yes	Yes	Yes	Yes	16
69	Hu et al	2004	Yes	No	Yes	Yes	Yes	Yes	Yes	Yes	Yes	16
70	Hudu et al	2018	Yes	No	Yes	Yes	Yes	Yes	Yes	Yes	Yes	16
71	Hyun-Ju et al	2007	Yes	No	Yes	Yes	Yes	Yes	Yes	Yes	Yes	16
72	Inui et al	2007	Yes	No	Yes	Yes	Yes	Yes	Yes	Yes	Yes	16
73	Ivana et al	2013	Yes	No	Yes	Yes	Yes	Yes	Yes	Yes	Yes	16
74	Jagdish et al	2020	Yes	No	Yes	Yes	Yes	Yes	Yes	Yes	Yes	16
75	Jin et al	2010	Yes	No	Yes	Yes	Yes	Yes	Yes	Yes	Yes	16
76	Kailash et al	2011	Yes	No	Yes	Yes	Yes	Yes	Yes	Yes	Yes	16
77	Kaklikkaya et al	2012	Yes	No	Yes	Yes	Yes	Yes	Yes	Yes	Yes	16
78	Kamol et al	2007	Yes	No	Yes	Yes	Yes	Yes	Yes	Yes	Yes	16
79	Kana et al	2007	Yes	No	Yes	Yes	Yes	Yes	Yes	Yes	Yes	16
80	Karimi et al	2014	Yes	No	Yes	Yes	Yes	Yes	Yes	Yes	Yes	16
81	Kaya et al	2007	Yes	No	Yes	Yes	Yes	Yes	Yes	Yes	Yes	16
82	Kazuhiko et al	2009	Yes	No	Yes	Yes	Yes	Yes	Yes	Yes	Yes	16
83	kikuchi et al	2000	Yes	No	Yes	Yes	Yes	Yes	Yes	Yes	Yes	16
84	Kobayashi et al	2002	Yes	No	Yes	Yes	Yes	Yes	Yes	Yes	Yes	16
85	Komatsu et al	2015	Yes	No	Yes	Yes	Yes	Yes	Yes	Yes	Yes	16
86	Kwon et al	2018	Yes	No	Yes	Yes	Yes	Yes	Yes	Yes	Yes	16
87	Kyaw et al	2020	Yes	No	Yes	Yes	Yes	Yes	Yes	Yes	Yes	16
88	Leblebicioglu et al	2004	Yes	No	Yes	Yes	Yes	Yes	Yes	Yes	Yes	16
89	Lee et al	2003	Yes	No	Yes	Yes	Yes	Yes	Yes	Yes	Yes	16
90	Lei et al	2005	Yes	No	Yes	Yes	Yes	Yes	Yes	Yes	Yes	16
91	Li et al	2010	Yes	No	Yes	Yes	Yes	Yes	Yes	Yes	Yes	16
92	Li. et al	2011	Yes	No	Yes	Yes	Yes	Yes	Yes	Yes	Yes	16
93	Li ..et al	2011	Yes	No	Yes	Yes	Yes	Yes	Yes	Yes	Yes	16
94	Li et al.	2007	Yes	No	Yes	Yes	Yes	Yes	Yes	Yes	Yes	16
95	Li et al..	2006	Yes	No	Yes	Yes	Yes	Yes	Yes	Yes	Yes	16
96	Lin et al	2007	Yes	No	Yes	Yes	Yes	Yes	Yes	Yes	Yes	16
97	Liu. et al	2012	Yes	No	Yes	Yes	Yes	Yes	Yes	Yes	Yes	16
98	Liu.. et al	2004	Yes	No	Yes	Yes	Yes	Yes	Yes	Yes	Yes	16
99	Liu et al.	2002	Yes	No	Yes	Yes	Yes	Yes	Yes	Yes	Yes	16
100	Liu et al	2010	Yes	No	Yes	Yes	Yes	Yes	Yes	Yes	Yes	16
101	Lu. et al	2007	Yes	No	Yes	Yes	Yes	Yes	Yes	Yes	Yes	16
102	Lu.. et al	2007	Yes	No	Yes	Yes	Yes	Yes	Yes	Yes	Yes	16
103	Lu et al	2011	Yes	No	Yes	Yes	Yes	Yes	Yes	Yes	Yes	16
104	Lusida et al	2003	Yes	No	Yes	Yes	Yes	Yes	Yes	Yes	Yes	16
105	Ma et al	2011	Yes	No	Yes	Yes	Yes	Yes	Yes	Yes	Yes	16
106	Mahmood et al	2016	Yes	No	Yes	Yes	Yes	Yes	Yes	Yes	Yes	16
107	Manamperi et al	2011	Yes	No	Yes	Yes	Yes	Yes	Yes	Yes	Yes	16
108	Matsuura et al	2009	Yes	No	Yes	Yes	Yes	Yes	Yes	Yes	Yes	16
109	Modhusudon et al	2016	Yes	No	Yes	Yes	Yes	Yes	Yes	Yes	Yes	16
110	Mojiri et al	2008	Yes	No	Yes	Yes	Yes	Yes	Yes	Yes	Yes	16
111	Mousavi. et al	2014	Yes	No	Yes	Yes	Yes	Yes	Yes	Yes	Yes	16
112	Mousavi et al	2014	Yes	No	Yes	Yes	Yes	Yes	Yes	Yes	Yes	16
113	Nei et al	2012	Yes	No	Yes	Yes	Yes	Yes	Yes	Yes	Yes	16
114	Neisi et al	2011	Yes	No	Yes	Yes	Yes	Yes	Yes	Yes	Yes	16
115	Nguyen et al	2008	Yes	No	Yes	Yes	Yes	Yes	Yes	Yes	Yes	16
116	Ni et al	2004	Yes	No	Yes	Yes	Yes	Yes	Yes	Yes	Yes	16
117	Norouzi et al	2012	Yes	No	Yes	Yes	Yes	Yes	Yes	Yes	Yes	16
118	Ogawa et al	2002	Yes	Yes	Yes	Yes	Yes	Yes	Yes	Yes	Yes	18
119	Oguzoglu et al	2001	Yes	No	Yes	Yes	Yes	Yes	Yes	Yes	Yes	16
120	Onganar et	2006	Yes	No	Yes	Yes	Yes	Yes	Yes	Yes	Yes	16
121	Ozdemir et al	2005	Yes	No	Yes	Yes	Yes	Yes	Yes	Yes	Yes	16
122	Panicker et al	2021	Yes	Yes	Yes	Yes	Yes	Yes	Yes	Yes	Yes	18
123	Parichat et al	2015	Yes	No	Yes	Yes	Yes	Yes	Yes	Yes	Yes	16
124	Pattaratida et al	2010	Yes	No	Yes	Yes	Yes	Yes	Yes	Yes	Yes	16
125	Paudel et al	2015	Yes	Yes	Yes	Yes	Yes	Yes	Yes	Yes	Yes	18
126	Perumal et al	2004	Yes	No	Yes	Yes	Yes	Yes	Yes	Yes	Yes	16
127	Pinarbasi et al	2008	Yes	No	Yes	Yes	Yes	Yes	Yes	Yes	Yes	16

128	Prapan et al	2008	Yes	Yes	Yes	Yes	Yes	Yes	Yes	Yes	Yes	18
129	Premashis et al	2021	Yes	Yes	Yes	Yes	Yes	Yes	Yes	Yes	Yes	18
130	Priyo et al	2016	Yes	Yes	Yes	Yes	Yes	Yes	Yes	Yes	Yes	18
131	Rafatpanah et al	2008	Yes	No	Yes	Yes	Yes	Yes	Yes	Yes	Yes	16
132	Rana et al	2019	Yes	No	Yes	Yes	Yes	Yes	Yes	Yes	Yes	16
133	Retno et al	1991	Yes	No	Yes	Yes	Yes	Yes	Yes	Yes	Yes	16
134	Saeeda. et al	2008	Yes	No	Yes	Yes	Yes	Yes	Yes	Yes	Yes	16
135	Saeeda et al	2007	Yes	No	Yes	Yes	Yes	Yes	Yes	Yes	Yes	16
136	Sahapat et al	2012	Yes	No	Yes	Yes	Yes	Yes	Yes	Yes	Yes	16
137	Said et al	2014	Yes	No	Yes	Yes	Yes	Yes	Yes	Yes	Yes	16
138	Saif et al	2017	Yes	No	Yes	Yes	Yes	Yes	Yes	Yes	Yes	16
139	Saket. et al	2006	Yes	No	Yes	Yes	Yes	Yes	Yes	Yes	Yes	16
140	Saket et al	2006	Yes	No	Yes	Yes	Yes	Yes	Yes	Yes	Yes	16
141	Salman et al	2019	Yes	Yes	Yes	Yes	Yes	Yes	Yes	Yes	Yes	18
142	Samad et al	2009	Yes	No	Yes	Yes	Yes	Yes	Yes	Yes	Yes	16
143	Santosh et al	2006	Yes	No	Yes	Yes	Yes	Yes	Yes	Yes	Yes	16
144	Sayan & Dogan	2012	Yes	No	Yes	Yes	Yes	Yes	Yes	Yes	Yes	16
145	Serin et al	2005	Yes	No	Yes	Yes	Yes	Yes	Yes	Yes	Yes	16
146	Shahnaz et al	2018	Yes	No	Yes	Yes	Yes	Yes	Yes	Yes	Yes	16
147	Shamima et al	2017	Yes	No	Yes	Yes	Yes	Yes	Yes	Yes	Yes	16
148	Shen et al	2015	Yes	No	Yes	Yes	Yes	Yes	Yes	Yes	Yes	16
149	Shi et al	2012	Yes	No	Yes	Yes	Yes	Yes	Yes	Yes	Yes	16
150	Smita et al	2020	Yes	No	Yes	Yes	Yes	Yes	Yes	Yes	Yes	16
151	Sobia et al	2011	Yes	No	Yes	Yes	Yes	Yes	Yes	Yes	Yes	16
152	Song. et al	2005	Yes	No	Yes	Yes	Yes	Yes	Yes	Yes	Yes	16
153	Song et al	2006	Yes	No	Yes	Yes	Yes	Yes	Yes	Yes	Yes	16
154	Srey et al	2006	Yes	No	Yes	Yes	Yes	Yes	Yes	Yes	Yes	16
155	Su et al	2020	Yes	No	Yes	Yes	Yes	Yes	Yes	Yes	Yes	16
156	Suda et al	2012	Yes	No	Yes	Yes	Yes	Yes	Yes	Yes	Yes	16
157	Sugauchi et al	2006	Yes	No	Yes	Yes	Yes	Yes	Yes	Yes	Yes	16
158	Sunbul and Leblebicioglu	2005	Yes	No	Yes	Yes	Yes	Yes	Yes	Yes	Yes	16
159	Sunbul et al	2003	Yes	No	Yes	Yes	Yes	Yes	Yes	Yes	Yes	16
160	Suzuki et al	2003	Yes	Yes	Yes	Yes	Yes	Yes	Yes	Yes	Yes	18
161	Swati et al	2003	Yes	No	Yes	Yes	Yes	Yes	Yes	Yes	Yes	16
162	Takako et al	2009	Yes	No	Yes	Yes	Yes	Yes	Yes	Yes	Yes	16
163	Talal et al	2004	Yes	No	Yes	Yes	Yes	Yes	Yes	Yes	Yes	16
164	Tamada et al	2011	Yes	Yes	Yes	Yes	Yes	Yes	Yes	Yes	Yes	18
165	Tanaka et al	2009	Yes	No	Yes	Yes	Yes	Yes	Yes	Yes	Yes	16
166	Tang et al	2007	Yes	No	Yes	Yes	Yes	Yes	Yes	Yes	Yes	16
167	Tao et al	2019	Yes	No	Yes	Yes	Yes	Yes	Yes	Yes	Yes	16
168	Thanh et al	2005	Yes	No	Yes	Yes	Yes	Yes	Yes	Yes	Yes	16
169	Torii et al	2013	Yes	No	Yes	Yes	Yes	Yes	Yes	Yes	Yes	16
170	Tran et al	2008	Yes	No	Yes	Yes	Yes	Yes	Yes	Yes	Yes	16
171	Usuda et al	1999	Yes	No	Yes	Yes	Yes	Yes	Yes	Yes	Yes	16
172	Wada et al	2014	Yes	No	Yes	Yes	Yes	Yes	Yes	Yes	Yes	16
173	Wahyuni et al	2019	Yes	No	Yes	Yes	Yes	Yes	Yes	Yes	Yes	16
174	Wang. et al	2014	Yes	No	Yes	Yes	Yes	Yes	Yes	Yes	Yes	16
175	Wang.. et al	2012	Yes	No	Yes	Yes	Yes	Yes	Yes	Yes	Yes	16
176	Wang et al	2018	Yes	No	Yes	Yes	Yes	Yes	Yes	Yes	Yes	16
177	Wei et al	2015	Yes	No	Yes	Yes	Yes	Yes	Yes	Yes	Yes	16
178	Wen and Tan	2004	Yes	No	Yes	Yes	Yes	Yes	Yes	Yes	Yes	16
179	Wu et al	2012	Yes	No	Yes	Yes	Yes	Yes	Yes	Yes	Yes	16
180	Xia et al	2001	Yes	No	Yes	Yes	Yes	Yes	Yes	Yes	Yes	16
181	Xiang et al	2012	Yes	No	Yes	Yes	Yes	Yes	Yes	Yes	Yes	16
182	Xin et al	2002	Yes	No	Yes	Yes	Yes	Yes	Yes	Yes	Yes	16
183	Yan. et al	2010	Yes	Yes	Yes	Yes	Yes	Yes	Yes	Yes	Yes	18
184	yahya et al	2010	Yes	No	Yes	Yes	Yes	Yes	Yes	Yes	Yes	16
185	Yan et al	2015	Yes	No	Yes	Yes	Yes	Yes	Yes	Yes	Yes	16
186	Yanag et al	2017	Yes	No	Yes	Yes	Yes	Yes	Yes	Yes	Yes	16
187	Yashwant et al	2017	Yes	No	Yes	Yes	Yes	Yes	Yes	Yes	Yes	16
188	Ye. et al	2017	Yes	No	Yes	Yes	Yes	Yes	Yes	Yes	Yes	16
189	Ye et al	2021	Yes	No	Yes	Yes	Yes	Yes	Yes	Yes	Yes	16
190	Yeh et al	2004	Yes	No	Yes	Yes	Yes	Yes	Yes	Yes	Yes	16
191	Yin et al	2010	Yes	No	Yes	Yes	Yes	Yes	Yes	Yes	Yes	16
192	You et al	2008	Yes	No	Yes	Yes	Yes	Yes	Yes	Yes	Yes	16
193	youyun et al	2016	Yes	No	Yes	Yes	Yes	Yes	Yes	Yes	Yes	16

194	Zaatari et al	2007	Yes	No	Yes	Yes	Yes	Yes	Yes	Yes	Yes	16
195	Zang. et al	2009	Yes	No	Yes	Yes	Yes	Yes	Yes	Yes	Yes	16
196	Zang et al	2015	Yes	No	Yes	Yes	Yes	Yes	Yes	Yes	Yes	16
197	Zeng et al	2005	Yes	No	Yes	Yes	Yes	Yes	Yes	Yes	Yes	16
198	Zhang et al	2018	Yes	No	Yes	Yes	Yes	Yes	Yes	Yes	Yes	16
199	Zhao et al	2001	Yes	No	Yes	Yes	Yes	Yes	Yes	Yes	Yes	16
200	Zhili et al	2014	Yes	No	Yes	Yes	Yes	Yes	Yes	Yes	Yes	16
201	Zhong et al	2005	Yes	No	Yes	Yes	Yes	Yes	Yes	Yes	Yes	16
202	Zhu et al	2008	Yes	No	Yes	Yes	Yes	Yes	Yes	Yes	Yes	16
203	Zhu. et al	2008	Yes	No	Yes	Yes	Yes	Yes	Yes	Yes	Yes	16
204	Zhu.. et al	2015	Yes	No	Yes	Yes	Yes	Yes	Yes	Yes	Yes	16
205	Zhuang et al	2007	Yes	No	Yes	Yes	Yes	Yes	Yes	Yes	Yes	16
206	Zohreh	2013	Yes	Yes	Yes	Yes	Yes	Yes	Yes	Yes	Yes	18
207	Zunaira et al	2010	Yes	No	Yes	Yes	Yes	Yes	Yes	Yes	Yes	16

JBI CHECKLIST* 1. Appropriate sampling frame to address target population, **2.** Appropriate sampling way of study participants, **3.** Adequate sample size, **4.** Detail description of study participants and settings, **5.** Data analysis with sufficient coverage of identified sample, **6.** Use of valid methods to identify the condition, **7.** Standard, reliable way of measurement of condition for all participants, **8.** Availability of appropriate statistical analysis, **9.** Adequate response rate and management of low response rate.

Scores are coded as Yes=2 and No=0.

Table S10. Subgroup analysis for comparison of prevalence of HBV genotypes in Asia across the methods of genotyping

Method	Number of studies	Prevalence (%)	95% CI	I^2 (%)	Q	Heterogeneity test	
						DF	*P*
Genotype A							
Sequencing	132	2.9	2.2 – 3.8	91.43	1529.03	131	<0.001
Multiplex PCR	26	3.5	1.9 – 6.3	93.49	383.88	25	<0.001
LIPA	13	2.2	1.0 – 4.7	81.83	66.03	12	<0.001
RFLP	30	4.5	2.9 – 7.1	91.91	358.254	29	<0.001
RDB hybridization	6	0.9	0.00 – 17.1	92.82	69.66	5	<0.001
Overall	207	3.1	2.5 – 3.8	91.79	2507.63	206	<0.001
Genotype B							
Sequencing	132	16.7	14.2 – 19.6	96.53	3773.10	131	<0.001
Multiplex PCR	26	29.3	19.5 – 41.4	98.29	1464.16	25	<0.001
LIPA	13	19.5	10.5 – 33.4	96.53	345.79	12	<0.001
RFLP	30	8.9	5.1 – 15.1	97.34	1088.76	29	<0.001
RDB hybridization	6	50.0	36.4 – 63.6	94.39	89.14	5	<0.001
Overall	207	17.8	15.5 – 20.4	97.26	7524.23	206	<0.001
Genotype C							
Sequencing	132	35.1	31.0 – 39.4	97.03	4407.51	131	<0.001
Multiplex PCR	26	23.6	15.1 – 34.7	98.23	1411.05	25	<0.001
LIPA	13	26.0	14.8 – 41.4	96.77	371.82	12	<0.001
RFLP	30	20.6	12.5 – 31.9	98.1	1530.08	29	<0.001
RDB hybridization	6	39.8	25.0 – 56.8	94.93	98.60	5	<0.001
Overall	207	30.9	27.5 – 34.5	97.57	8476.52	206	<0.001
Genotype D							
Sequencing	132	17.3	12.4 – 23.7	97.54	5325.73	131	<0.001
Multiplex PCR	26	6.8	3.3 – 13.5	97.13	872.11	25	<0.001
LIPA	13	7.7	1.5 – 31.6	95.82	286.75	12	<0.001
RFLP	30	36.5	20.3 – 56.6	97.51	1165.14	29	<0.001
RDB hybridization	6	0.6	0.2 – 1.7	35.46	7.75	5	0.171
Overall	207	15.4	11.8 – 19.8	97.37	7819.59	206	<0.001
Genotype E							
Sequencing	132	0.6	0.5 – 0.8	11.42	147.89	131	0.149
Multiplex PCR	26	0.6	0.3 – 1.2	67.7	77.40	25	<0.001
LIPA	13	0.5	0.3 – 1.0	0.00	4.08	12	0.982
RFLP	30	0.5	0.3 – 0.8	0.00	23.63	29	0.747
RDB hybridization	6	0.3	0.1 – 1.1	0.00	4.37	5	0.497
Overall	207	0.6	0.5 – 0.7	29.79	293.39	206	<0.001
Genotype F							
Sequencing	132	0.6	0.4 – 0.7	0.00	112.86	131	0.872
Multiplex PCR	26	0.5	0.2 – 1.7	89.9	247.41	25	<0.001
LIPA	13	0.5	0.3 – 1.0	0.00	3.66	12	0.989
RFLP	30	0.8	0.4 – 1.7	76.29	122.32	29	<0.001
RDB hybridization	6	0.3	0.1 – 1.1	0.00	4.37	5	0.497
Overall	207	0.6	0.4 – 0.8	75.26	832.73	206	<0.001
Genotype G							
Sequencing	132	0.6	0.4 – 0.7	0.00	114.454	131	0.848
Multiplex PCR	26	0.4	0.2 – 0.8	0.00	19.35	25	0.780
LIPA	13	0.4	0.2 – 0.8	0.00	2.93	12	0.996
RFLP	30	0.6	0.3 – 1.0	41.9	49.91	29	0.009
RDB hybridization	6	0.3	0.1 – 1.1	0.00	4.37	5	0.497
Overall	207	0.6	0.5 – 0.7	0.00	205.13	206	0.504
Genotype H							
Sequencing	132	0.6	0.4 – 0.7	0.00	109.04	131	0.919
Multiplex PCR	26	0.4	0.2 – 0.6	0.00	19.35	25	0.780
LIPA	13	0.4	0.2 – 0.8	0.00	2.93	12	0.996
RFLP	30	0.5	0.3 – 0.8	0.00	22.99	29	0.777
RDB hybridization	6	0.3	0.1 – 1.1	0.00	4.37	5	0.497
Overall	207	0.5	0.4 – 0.6	0.00	162.06	206	0.989
Genotype I							
Sequencing	132	0.8	0.6 – 1.0	0.00	127.99	131	0.558
Multiplex PCR	26	0.4	0.2 – 0.6	0.00	19.35	25	0.780
LIPA	13	0.4	0.2 – 0.8	0.00	2.93	12	0.996
RFLP	30	0.5	0.3 – 0.8	0.00	22.99	29	0.777
RDB hybridization	6	0.3	0.1 – 1.1	0.00	4.37	5	0.497

Overall	207	0.6	0.5 – 0.8	0.00	190.91	206	0.767

LIPA: Line probe assay, RFLP: restriction fragment length polymorphism, RDB: reverse dot blot

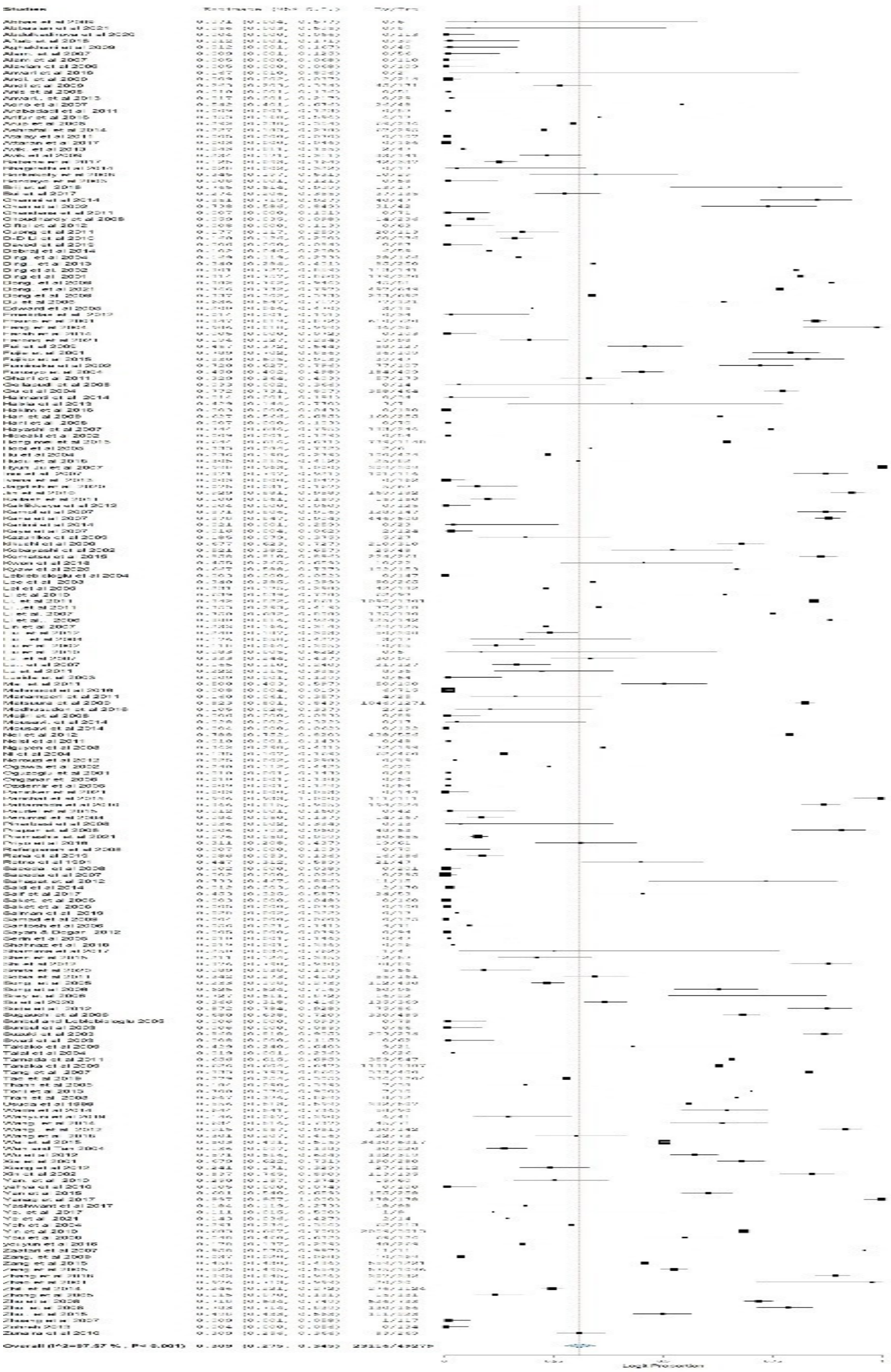

Figure 2. Forest plot showing the pooled prevalence of HBV genotype C in Asia.

PRISMA 2009 Checklist

Section/topic	#	Checklist item	Reported on page #
**TITLE**			
Title	1	Identify the report as a systematic review, meta-analysis, or both.	1
**ABSTRACT**			
Structured summary	2	Provide a structured summary including, as applicable: background; objectives; data sources; study eligibility criteria, participants, and interventions; study appraisal and synthesis methods; results; limitations; conclusions and implications of key findings; systematic review registration number.	2
**INTRODUCTION**			
Rationale	3	Describe the rationale for the review in the context of what is already known.	3-4
Objectives	4	Provide an explicit statement of questions being addressed with reference to participants, interventions, comparisons, outcomes, and study design (PICOS).	3-4
**METHODS**			
Protocol and registration	5	Indicate if a review protocol exists, if and where it can be accessed (e.g., Web address), and, if available, provide registration information including registration number.	6
Eligibility criteria	6	Specify study characteristics (e.g., PICOS, length of follow-up) and report characteristics (e.g., years considered, language, publication status) used as criteria for eligibility, giving rationale.	5
Information sources	7	Describe all information sources (e.g., databases with dates of coverage, contact with study authors to identify additional studies) in the search and date last searched.	5-6
Search	8	Present full electronic search strategy for at least one database, including any limits used, such that it could be repeated.	5
Study selection	9	State the process for selecting studies (i.e., screening, eligibility, included in systematic review, and, if applicable, included in the meta-analysis).	5-6
Data collection process	10	Describe method of data extraction from reports (e.g., piloted forms, independently, in duplicate) and any processes for obtaining and confirming data from investigators.	5-6
Data items	11	List and define all variables for which data were sought (e.g., PICOS, funding sources) and any assumptions and simplifications made.	5-6
Risk of bias in individual studies	12	Describe methods used for assessing risk of bias of individual studies (including specification of whether this was done at the study or outcome level), and how this information is to be used in any data synthesis.	5-6
Summary measures	13	State the principal summary measures (e.g., risk ratio, difference in means).	6
Synthesis of results	14	Describe the methods of handling data and combining results of studies, if done, including measures of consistency (e.g., I^2) for each meta-analysis.	6

PRISMA 2009 Checklist

Section/topic	#	Checklist item	Reported on page #
Risk of bias across studies	15	Specify any assessment of risk of bias that may affect the cumulative evidence (e.g., publication bias, selective reporting within studies).	6
Additional analyses	16	Describe methods of additional analyses (e.g., sensitivity or subgroup analyses, meta-regression), if done, indicating which were pre-specified.	6
**RESULTS**			
Study selection	17	Give numbers of studies screened, assessed for eligibility, and included in the review, with reasons for exclusions at each stage, ideally with a flow diagram.	7
Study characteristics	18	For each study, present characteristics for which data were extracted (e.g., study size, PICOS, follow-up period) and provide the citations.	7-11
Risk of bias within studies	19	Present data on risk of bias of each study and, if available, any outcome level assessment (see item 12).	12
Results of individual studies	20	For all outcomes considered (benefits or harms), present, for each study: (a) simple summary data for each intervention group (b) effect estimates and confidence intervals, ideally with a forest plot.	12
Synthesis of results	21	Present results of each meta-analysis done, including confidence intervals and measures of consistency.	12
Risk of bias across studies	22	Present results of any assessment of risk of bias across studies (see Item 15).	12
Additional analysis	23	Give results of additional analyses, if done (e.g., sensitivity or subgroup analyses, meta-regression [see Item 16]).	12-16
**DISCUSSION**			
Summary of evidence	24	Summarize the main findings including the strength of evidence for each main outcome; consider their relevance to key groups (e.g., healthcare providers, users, and policy makers).	19-21
Limitations	25	Discuss limitations at study and outcome level (e.g., risk of bias), and at review-level (e.g., incomplete retrieval of identified research, reporting bias).	20-21
Conclusions	26	Provide a general interpretation of the results in the context of other evidence, and implications for future research.	19-21
**FUNDING**			
Funding	27	Describe sources of funding for the systematic review and other support (e.g., supply of data); role of funders for the systematic review.	21

GENOTPE A

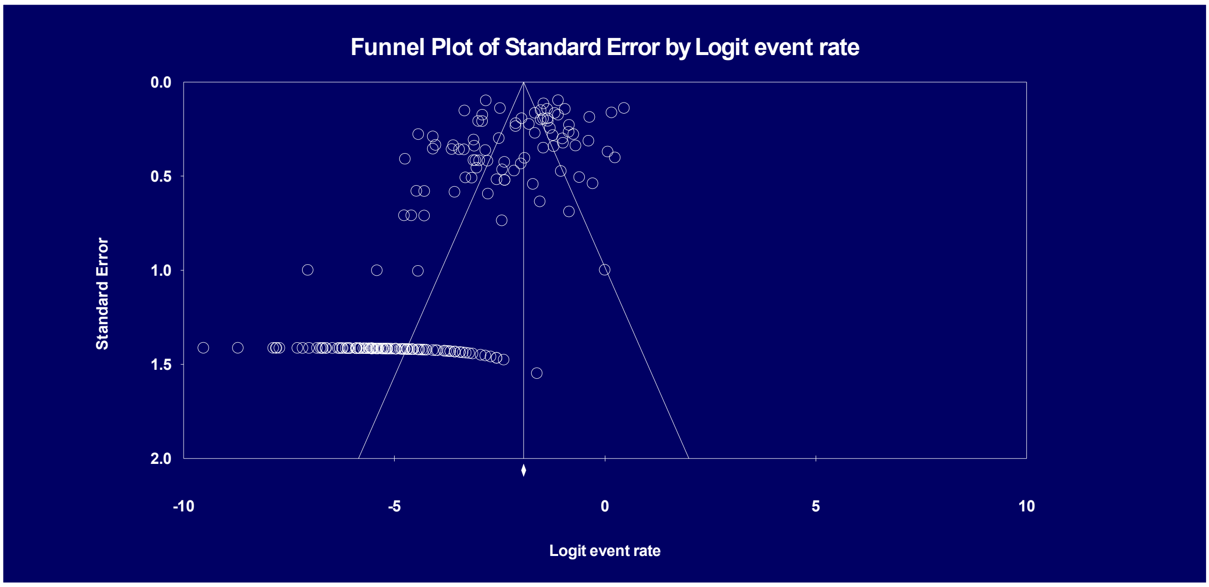

Egger p = 0.00000

GENPTYPE B

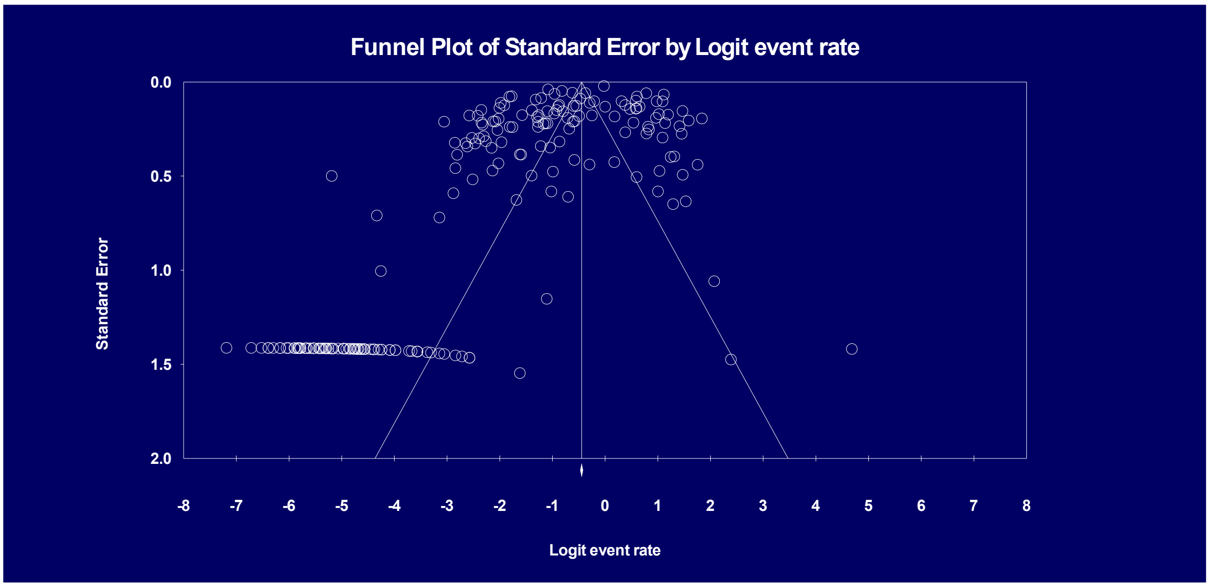

Egger p = 0.00000

GENOTYPE C

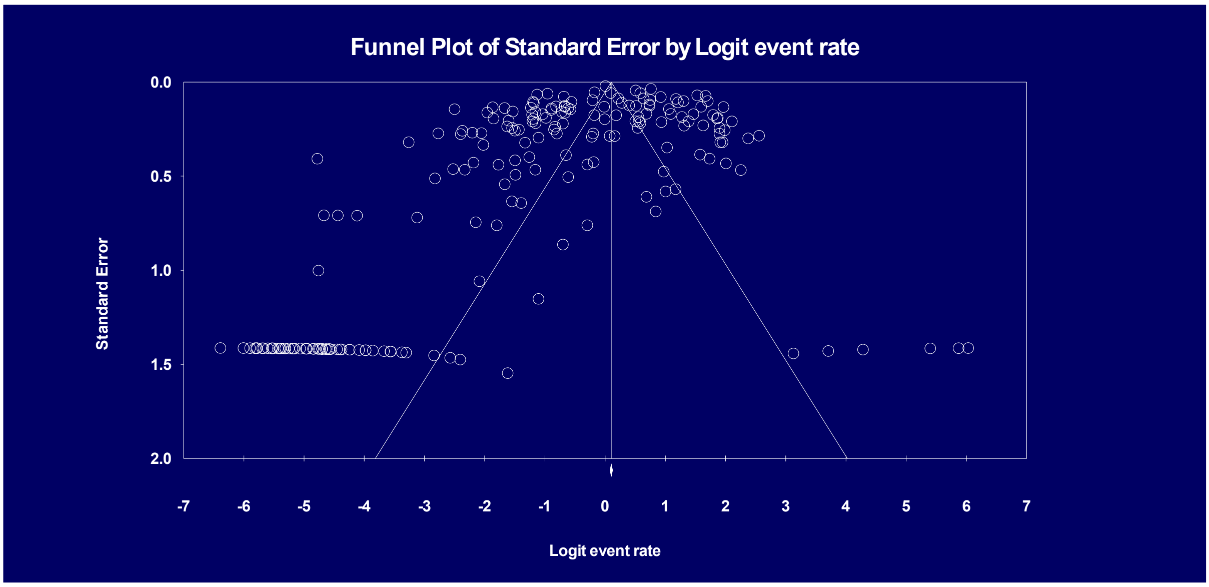

Egger p = 0.00001

GENOTYPE D

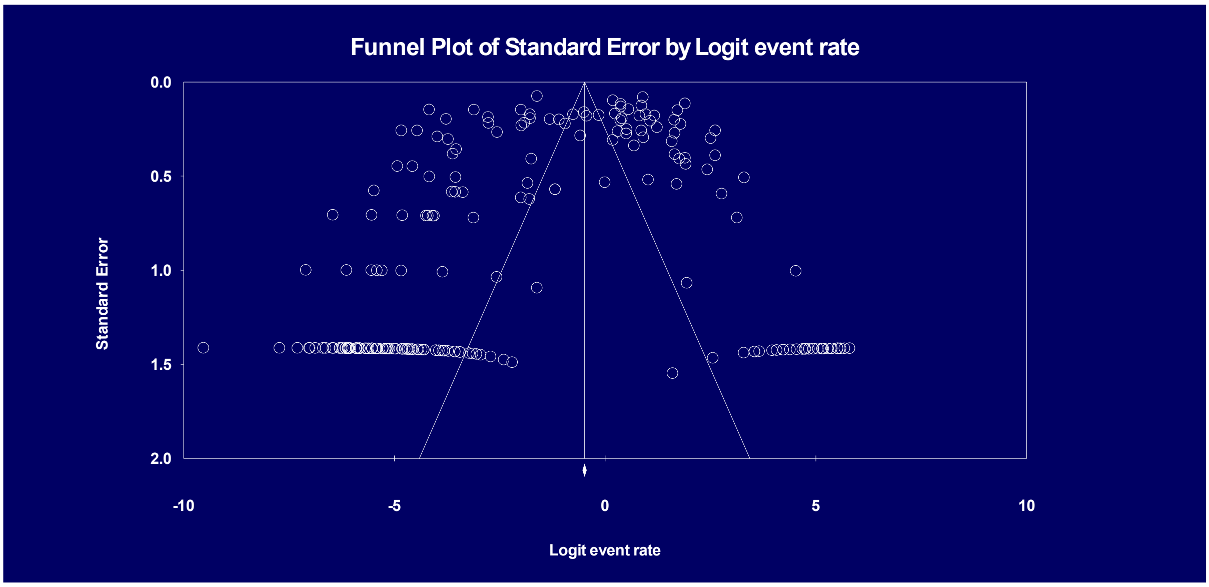

Egger p = 0.00043

GENOTYPE E

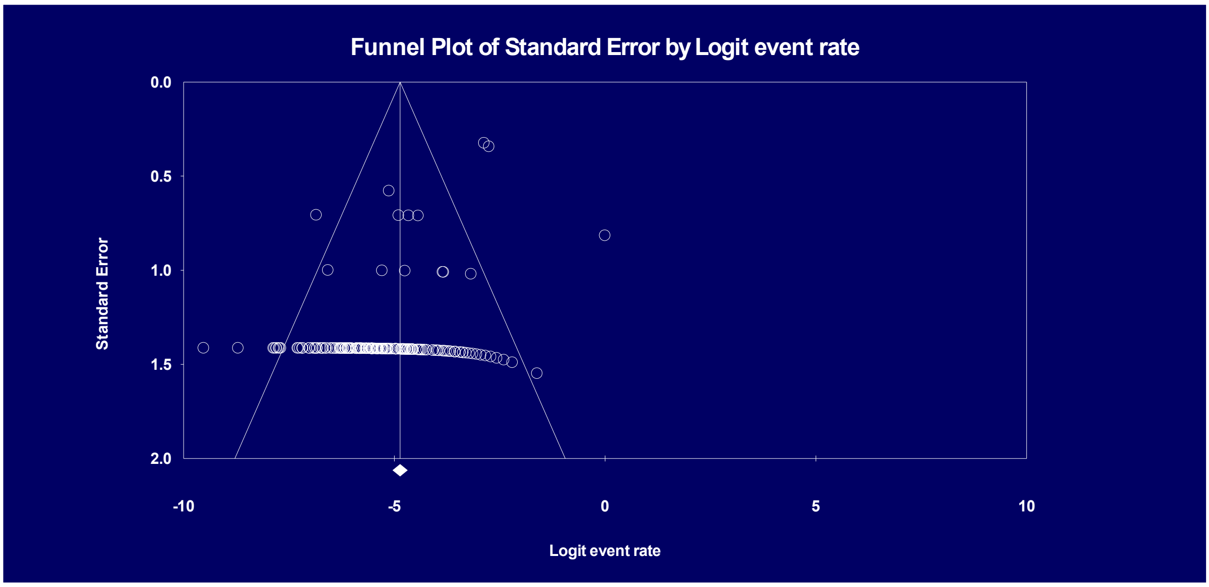

Egger p = 0.00000

GENOTYPE F

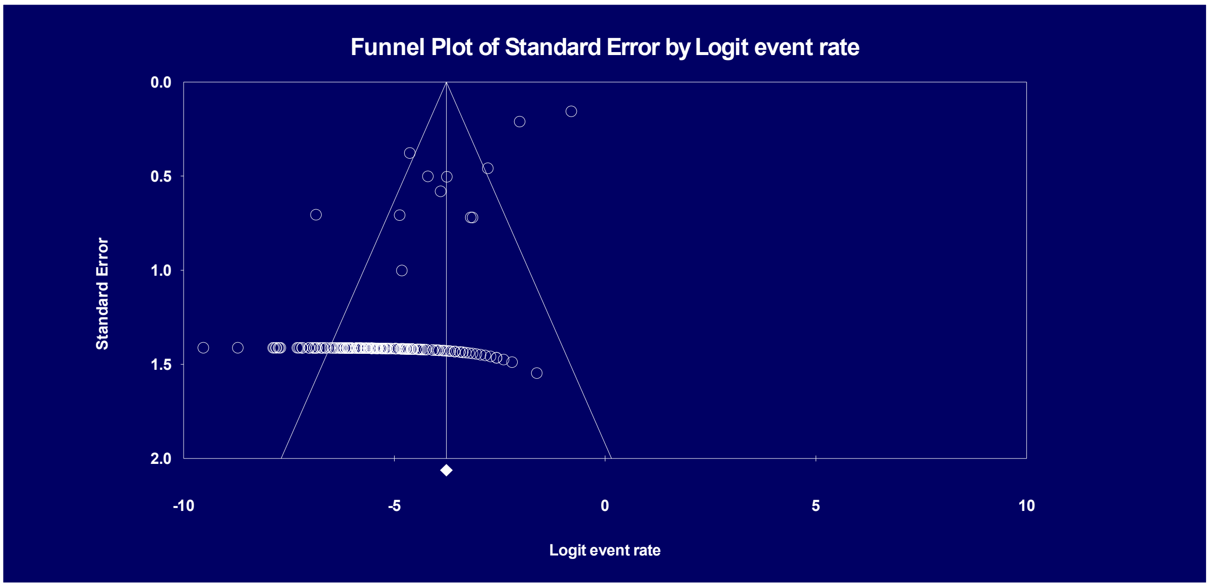

Egger p = 0.00000

GENOTYPE G

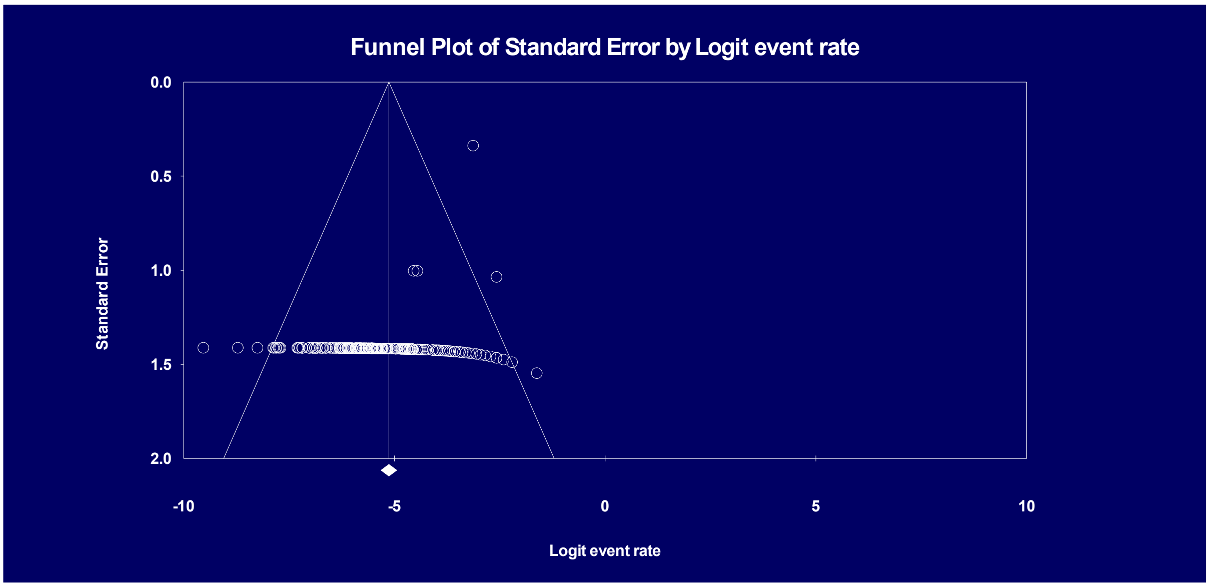

Egger p = 0.00000

GENOTYPE H

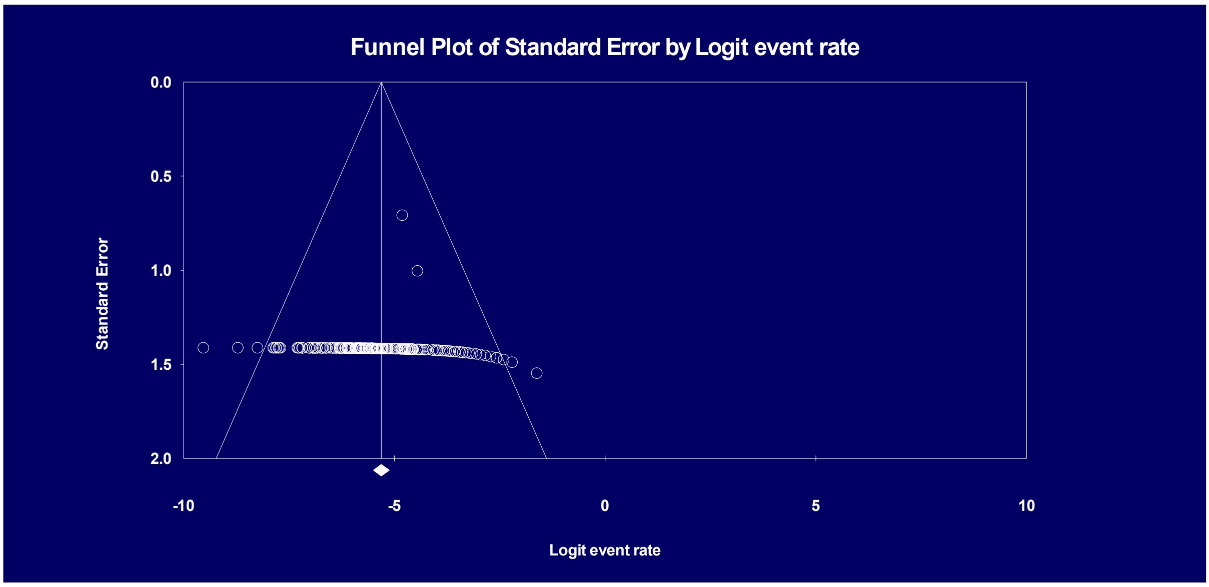

Egger p = 0.88283

GENOTYPE I

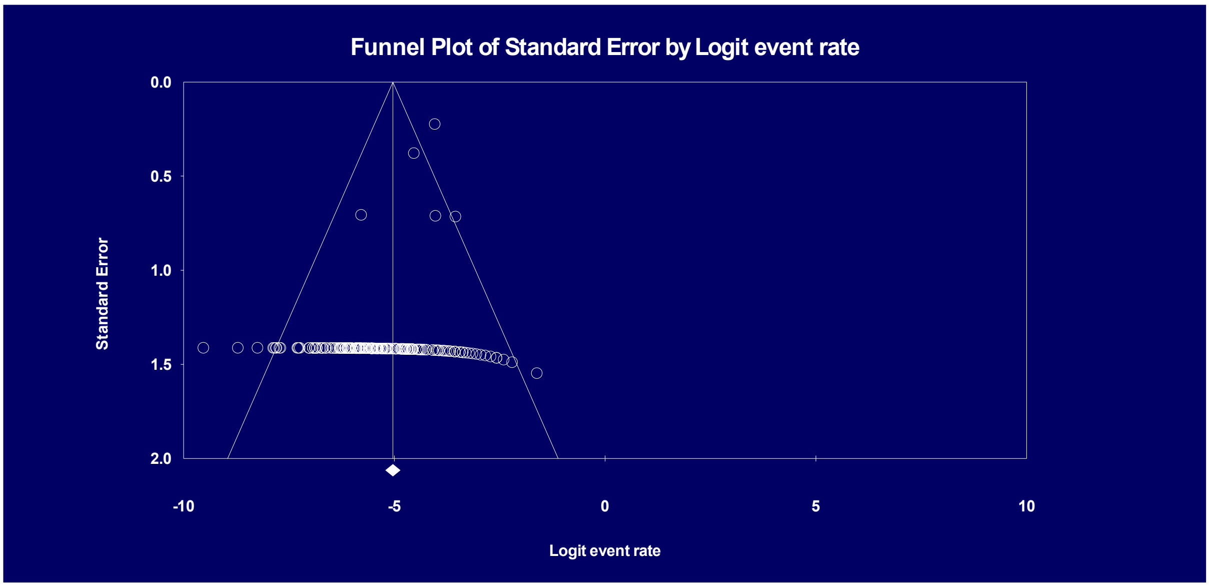

Egger p = 0.00000

FOREST PLOT A

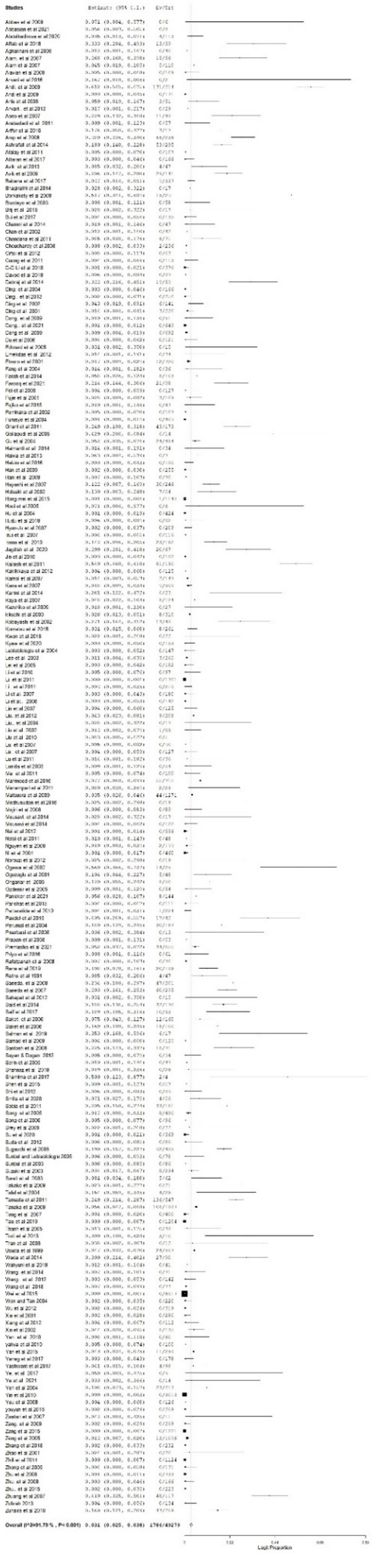

FOREST PLOT B

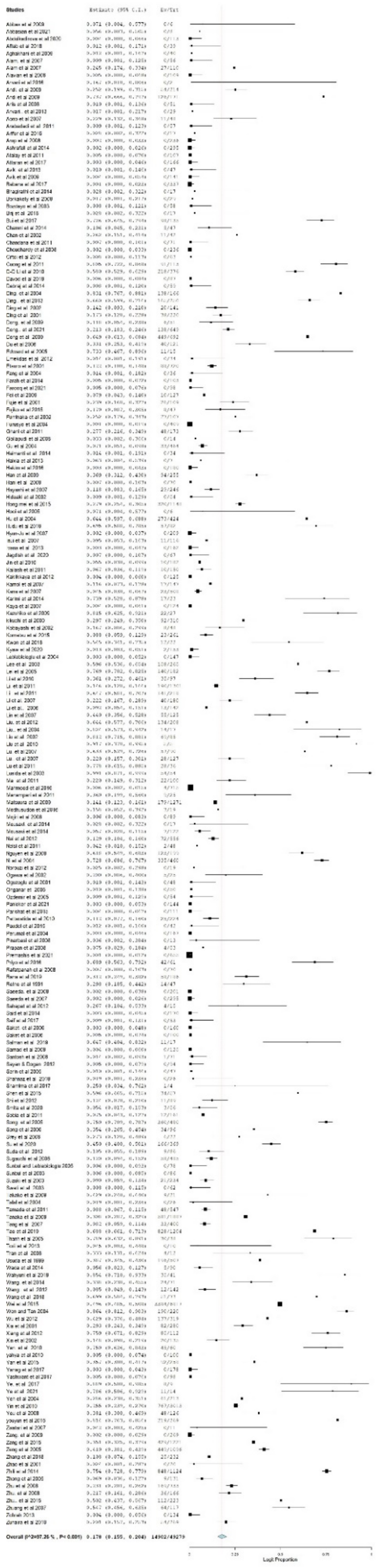

FOREST PLOT C

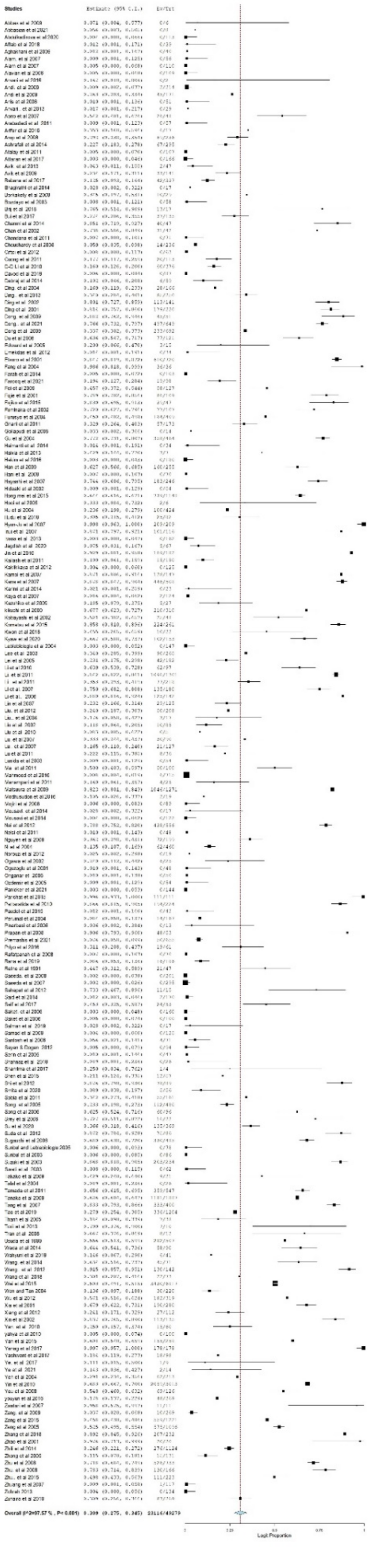

FOREST PLOT D

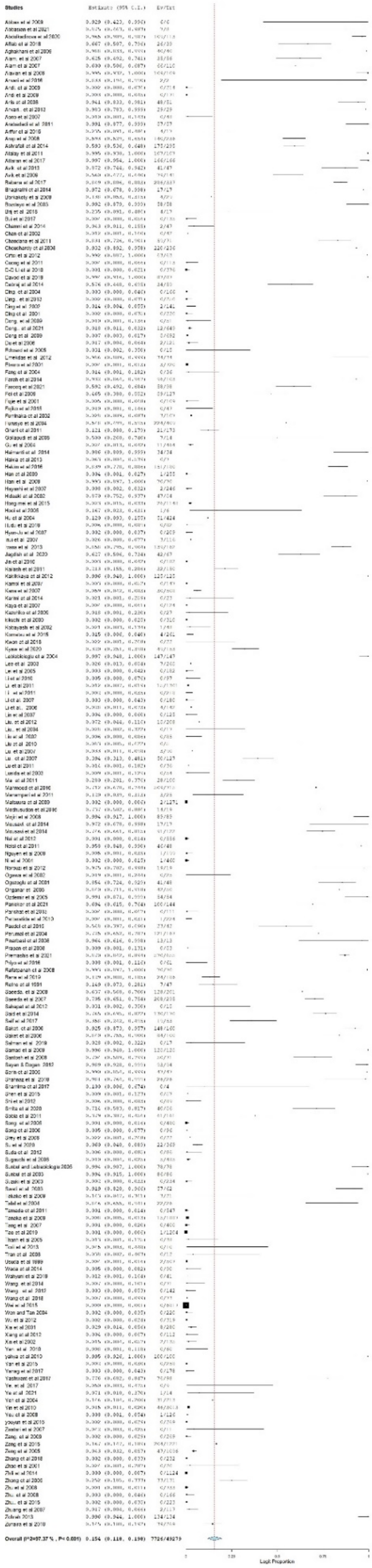

FOREST PLOT E

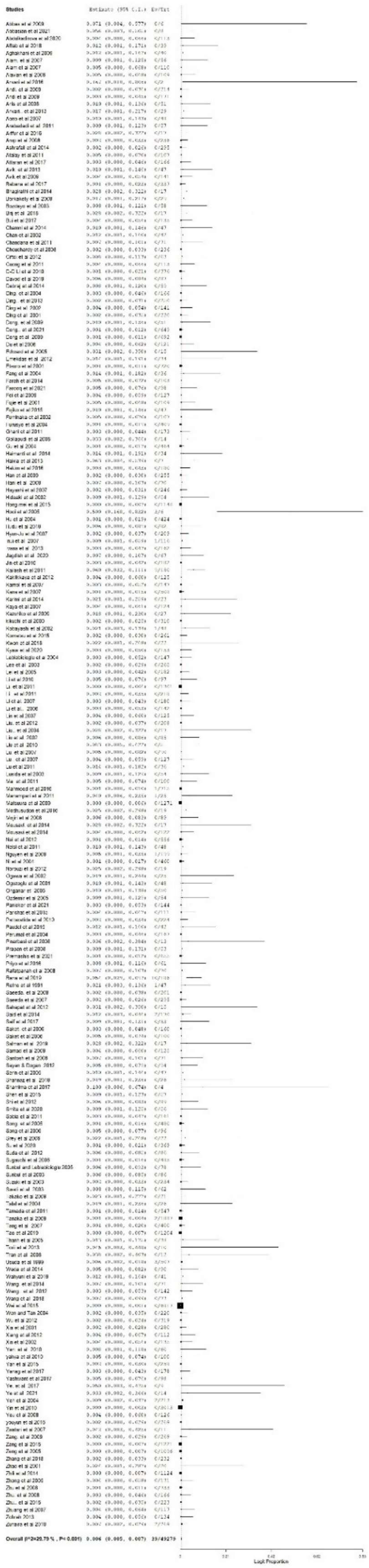

FOREST PLOT F

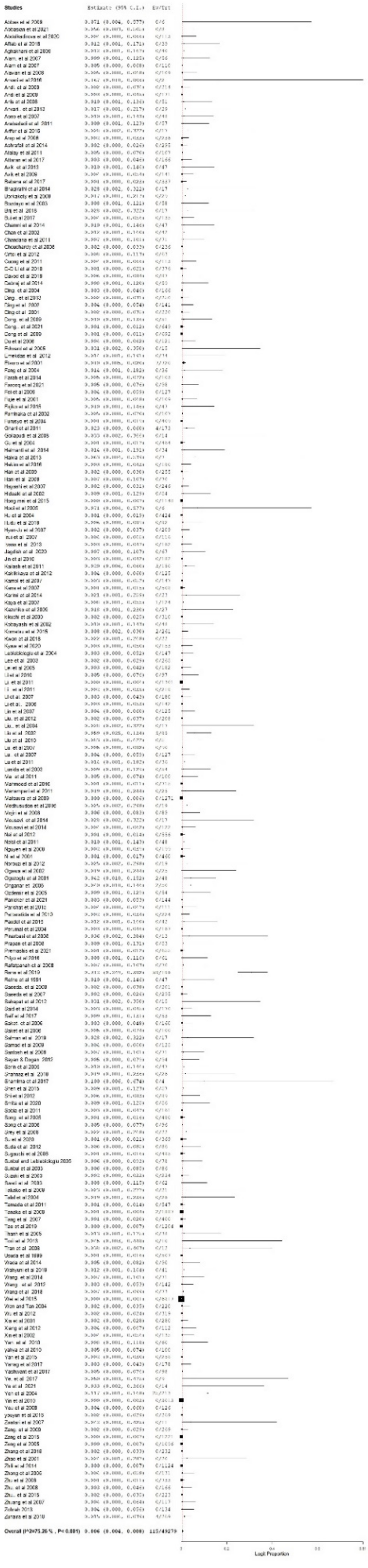

FOREST PLOT G

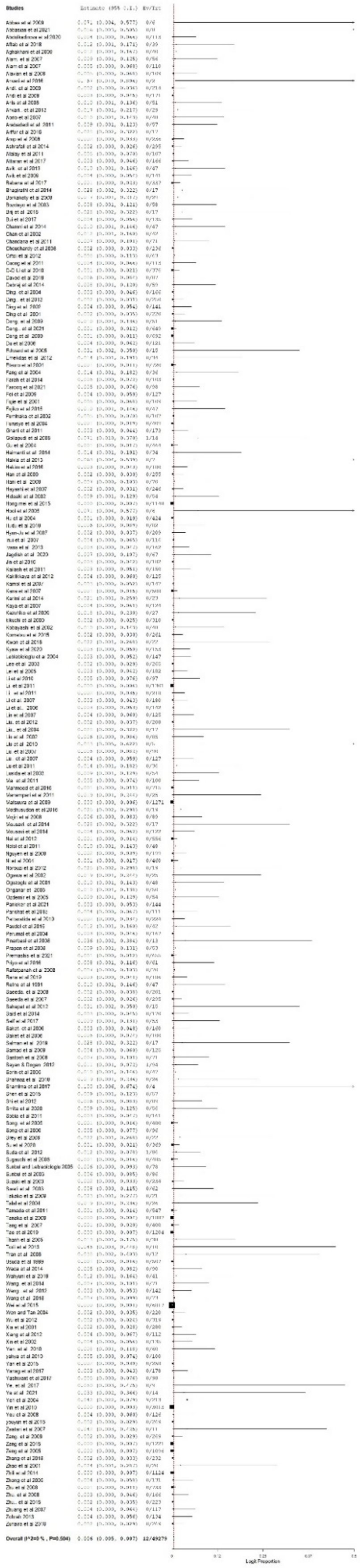

FOREST PLOT H

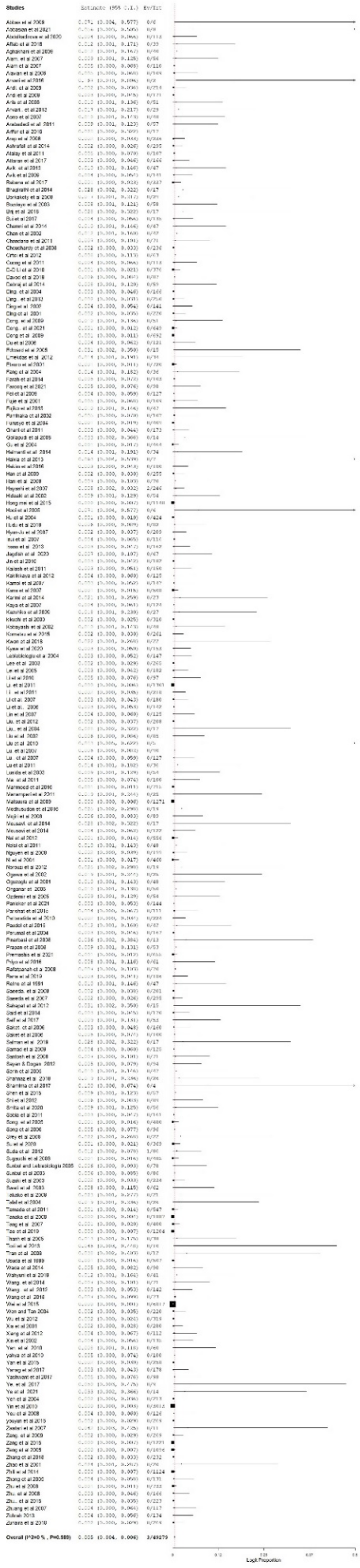

FOREST PLOT I

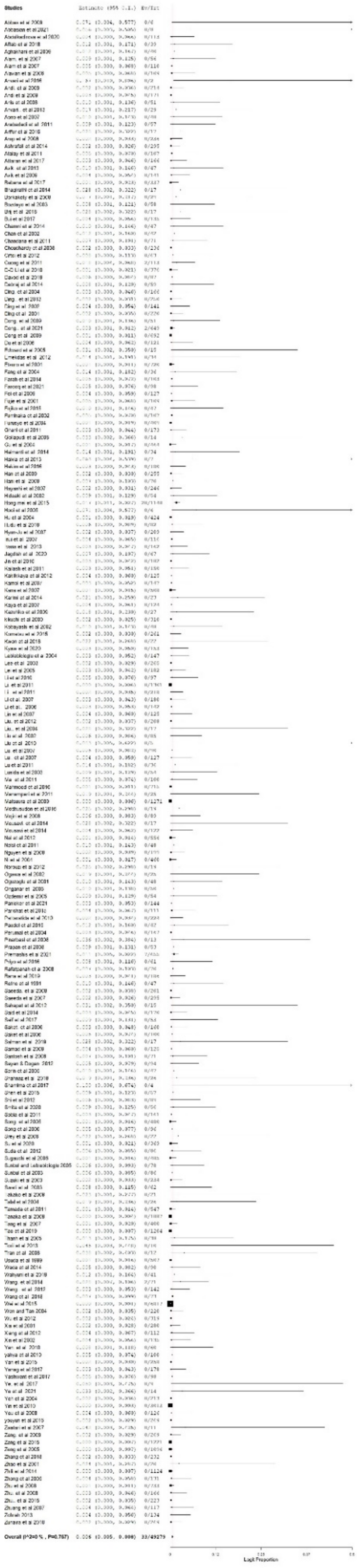

SUBGROUP FOREST PLOT A

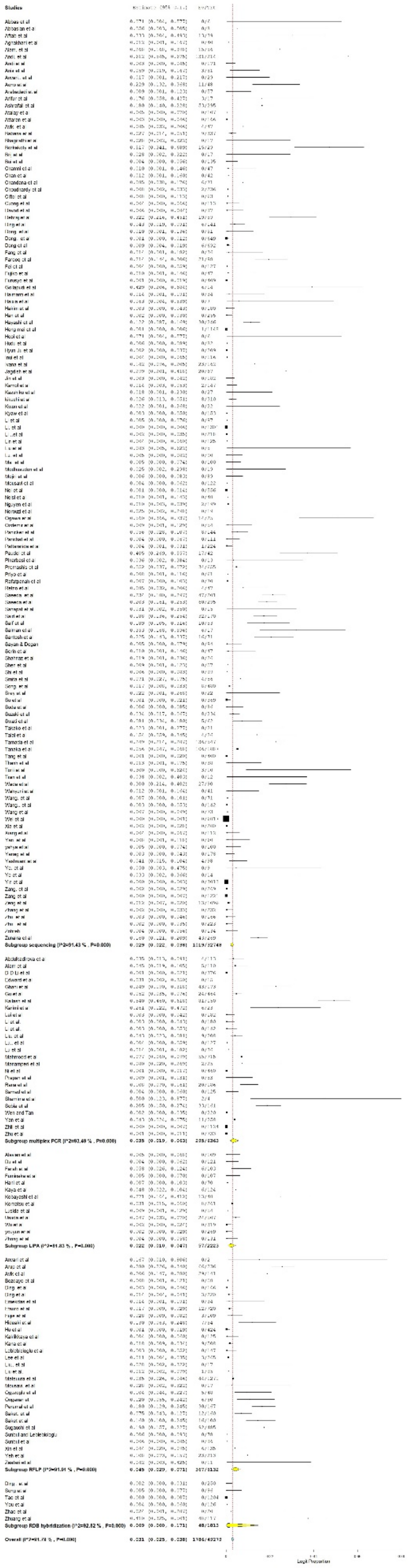

SUBGROUP FOREST PLOT B

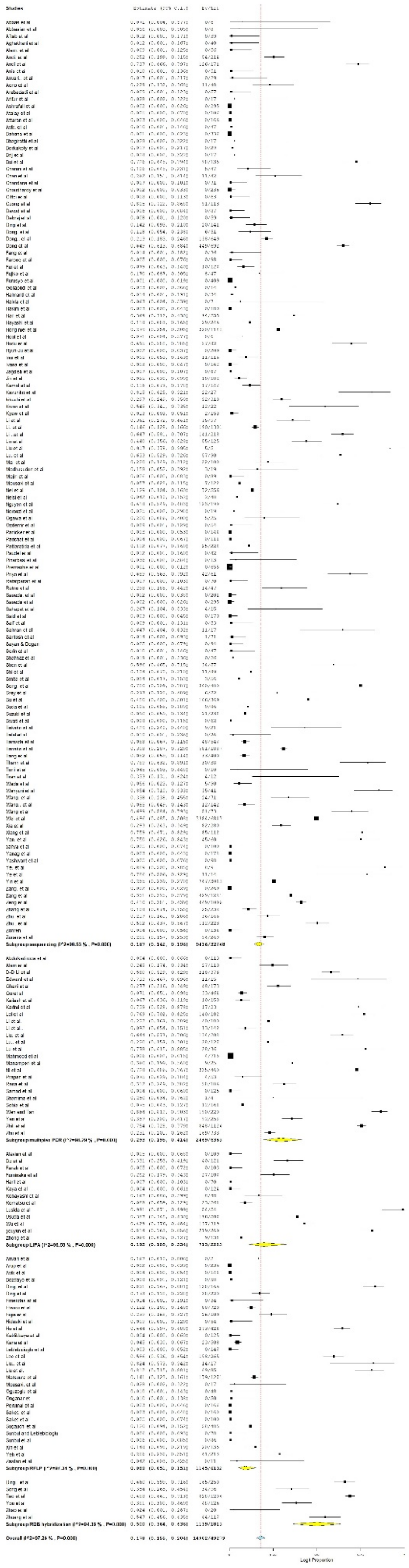

SUBGROUP FOREST PLOT C

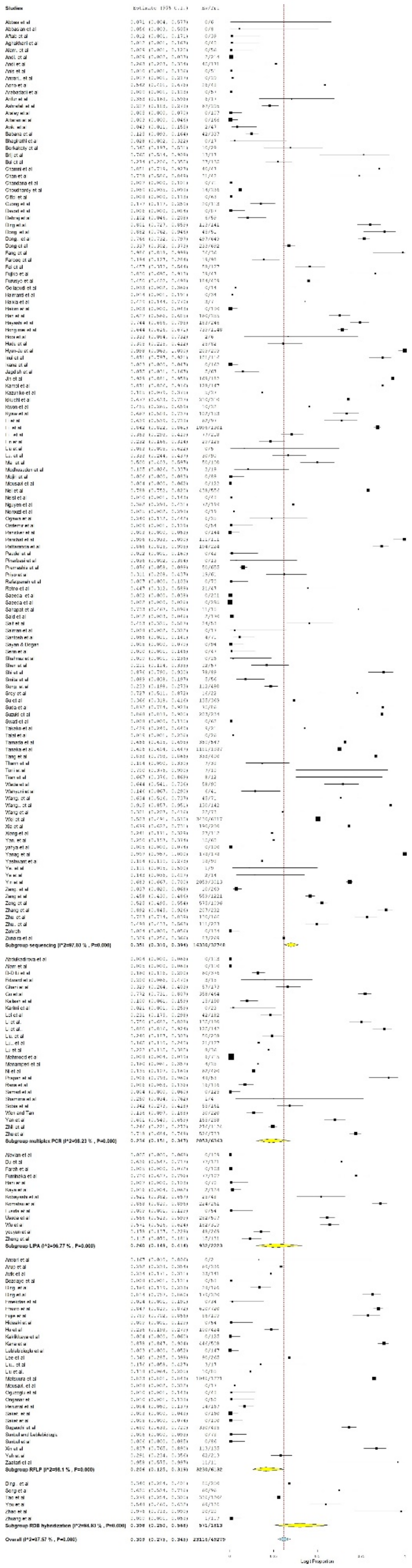

SUBGROUP FOREST PLOT D

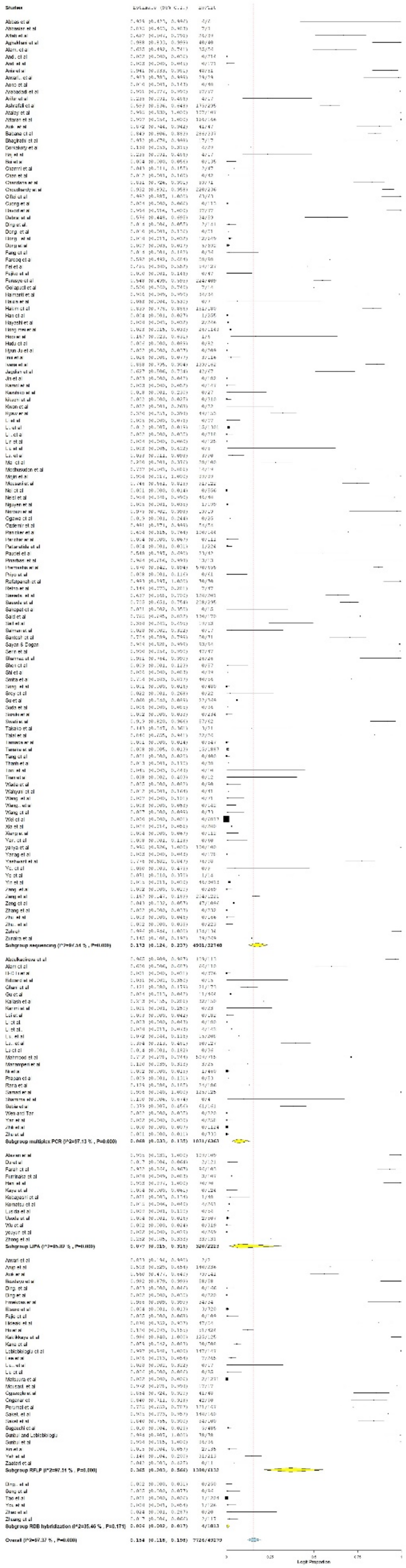

SUBGROUP FOREST PLOT E

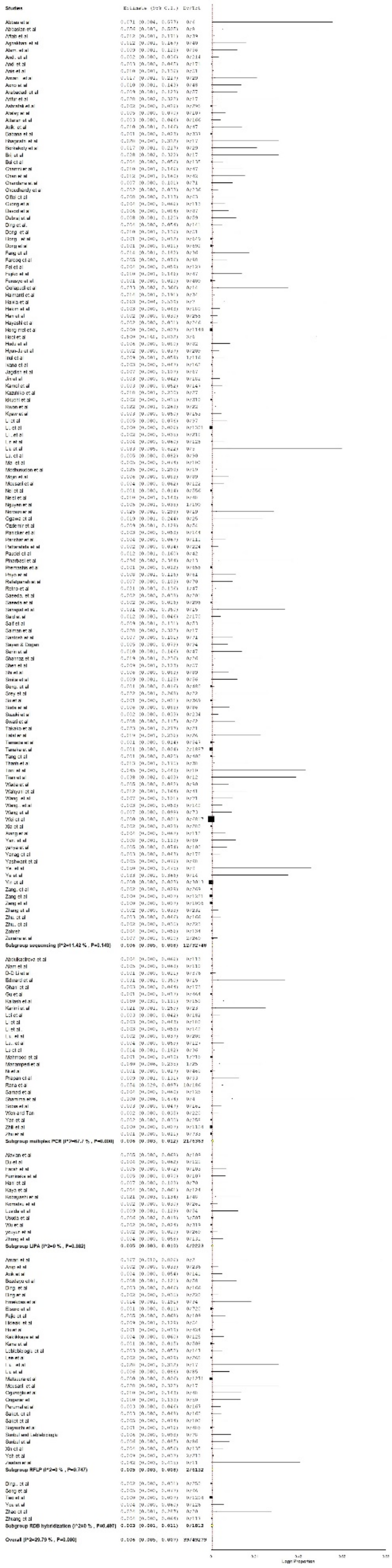

SUBGROUP FOREST PLOT F

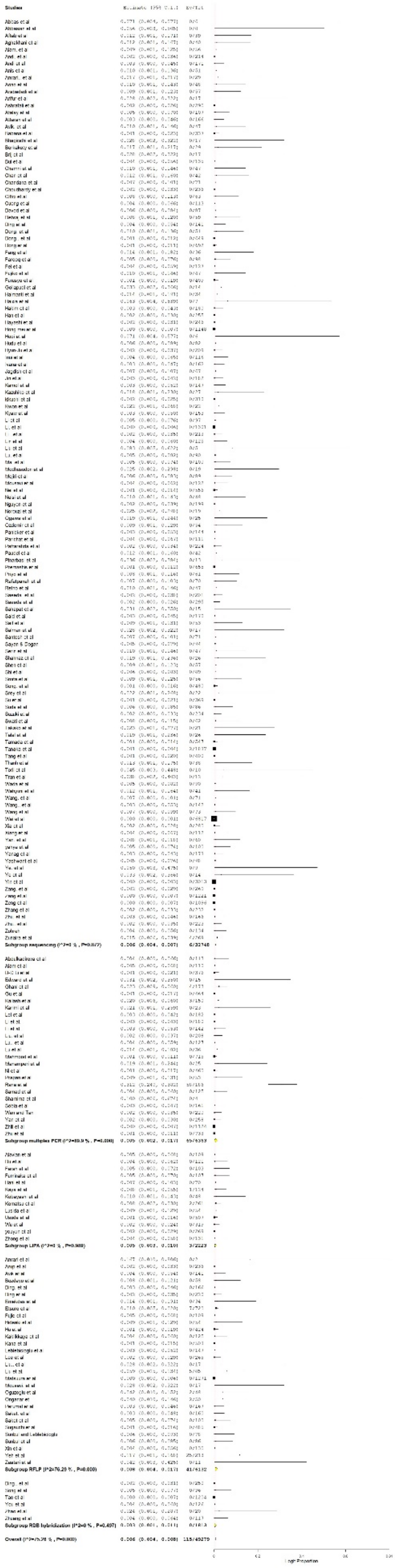

SUBGROUP FOREST PLOT G

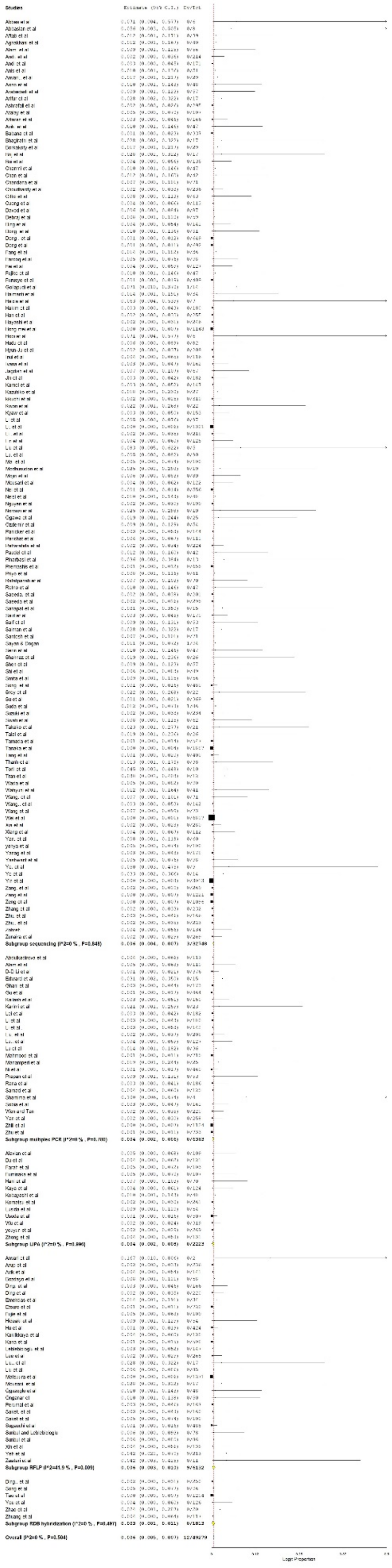

SUBGROUP FOREST PLOT H

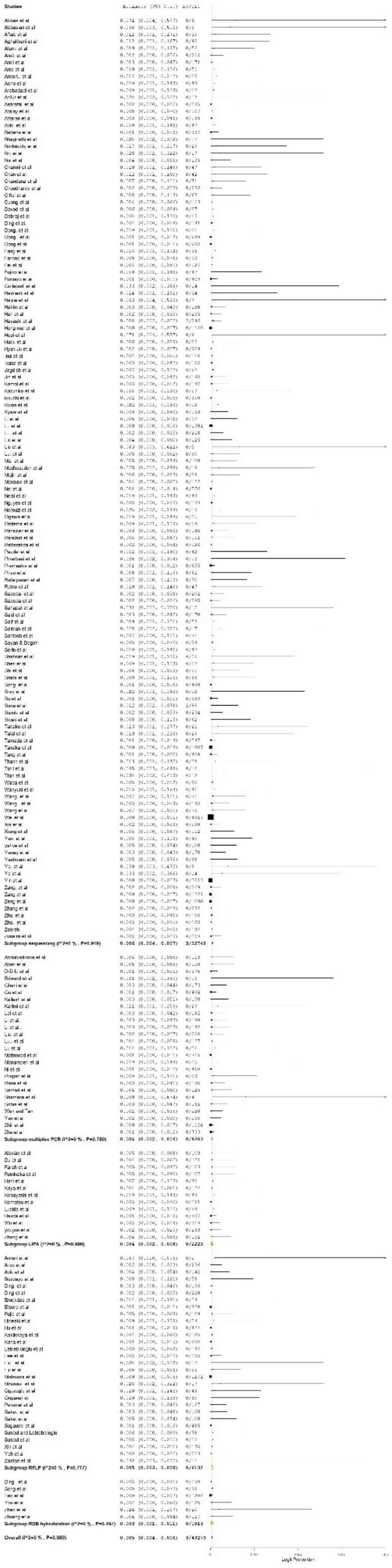

SUBGROUP FOREST PLOT I

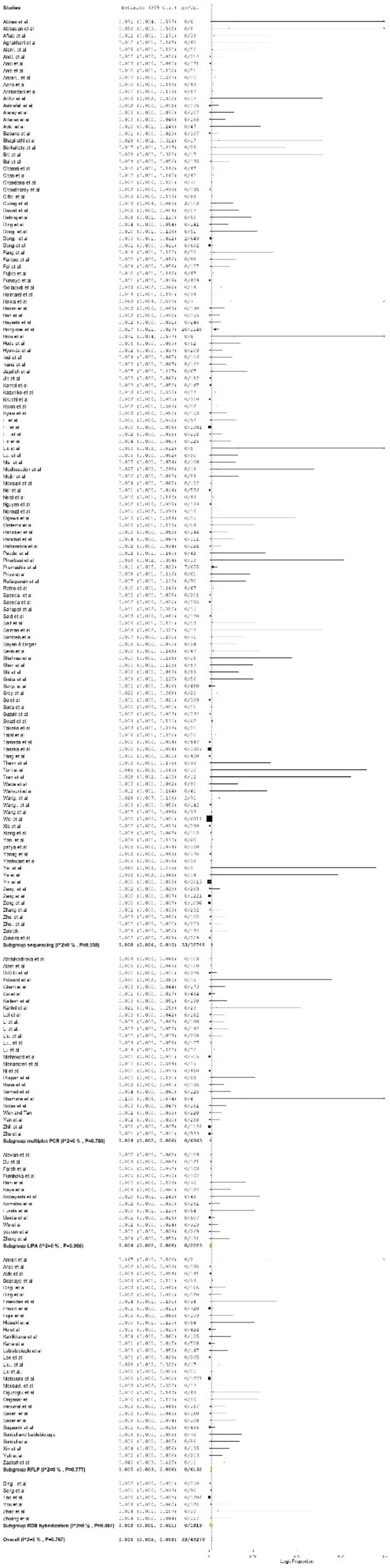

SUBGROUP FOREST PLOT A

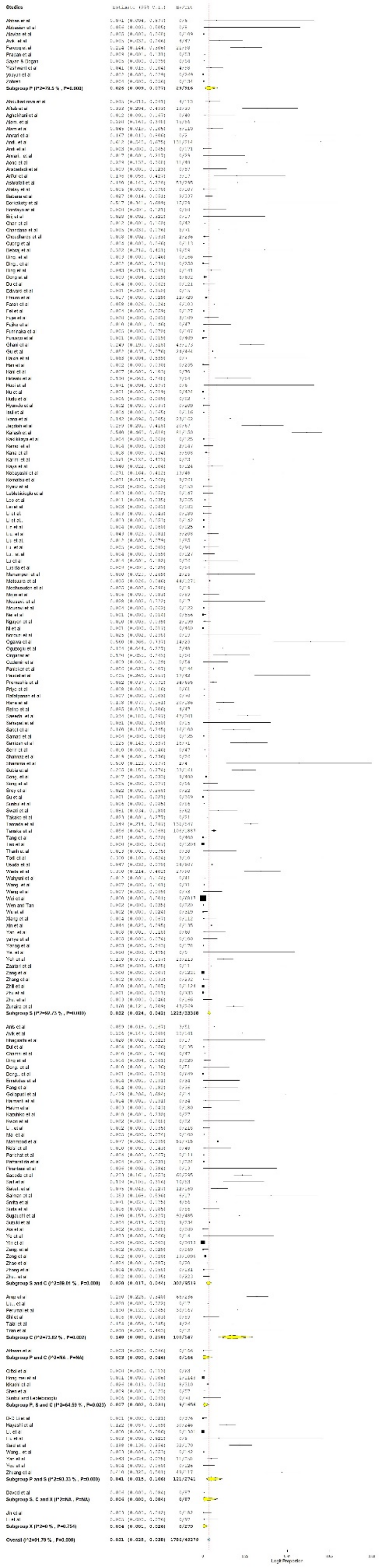

SUBGROUP FOREST PLOT B

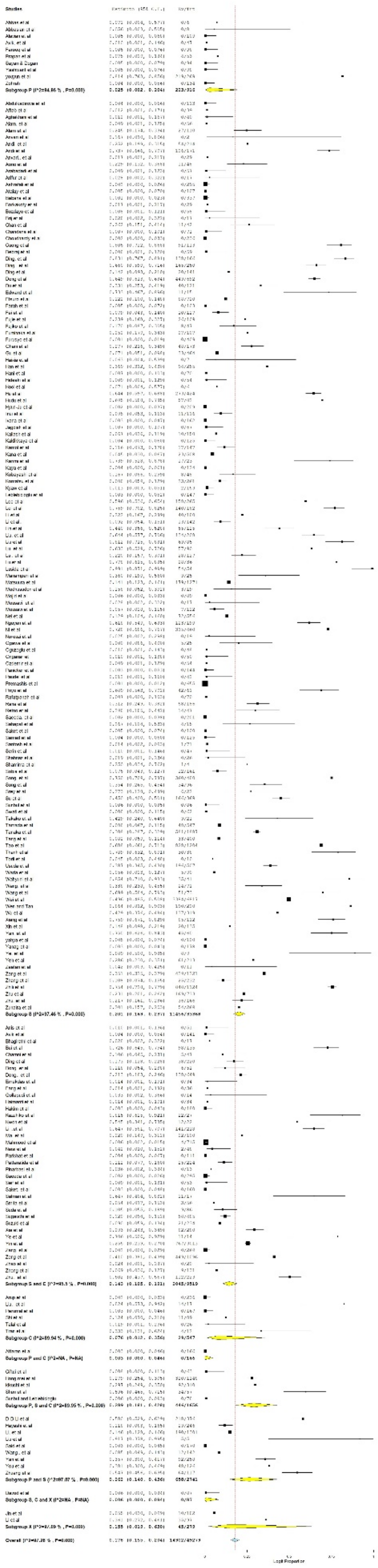

SUBGROUP FOREST PLOT C

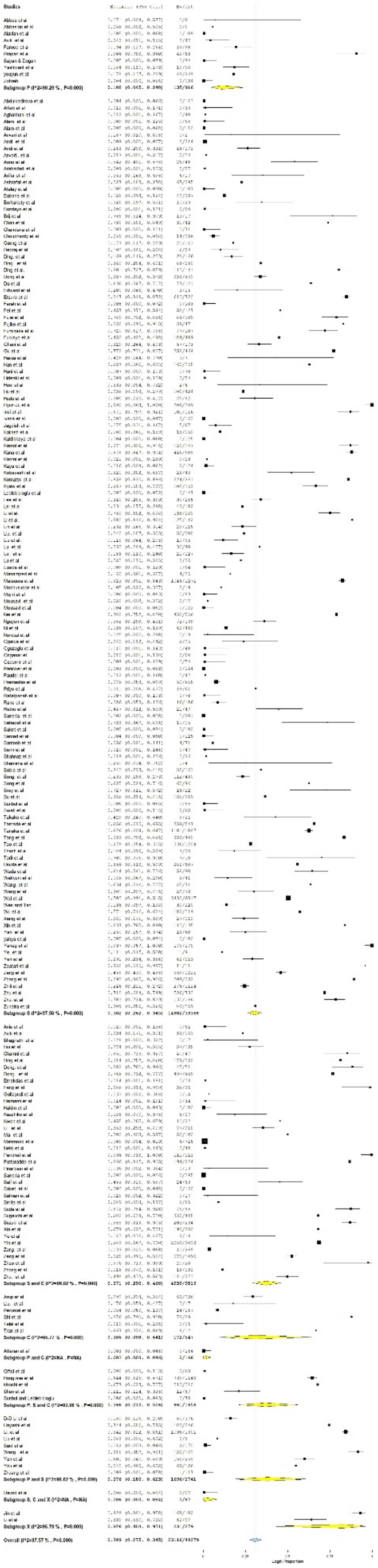

SUBGROUP FOREST PLOT D

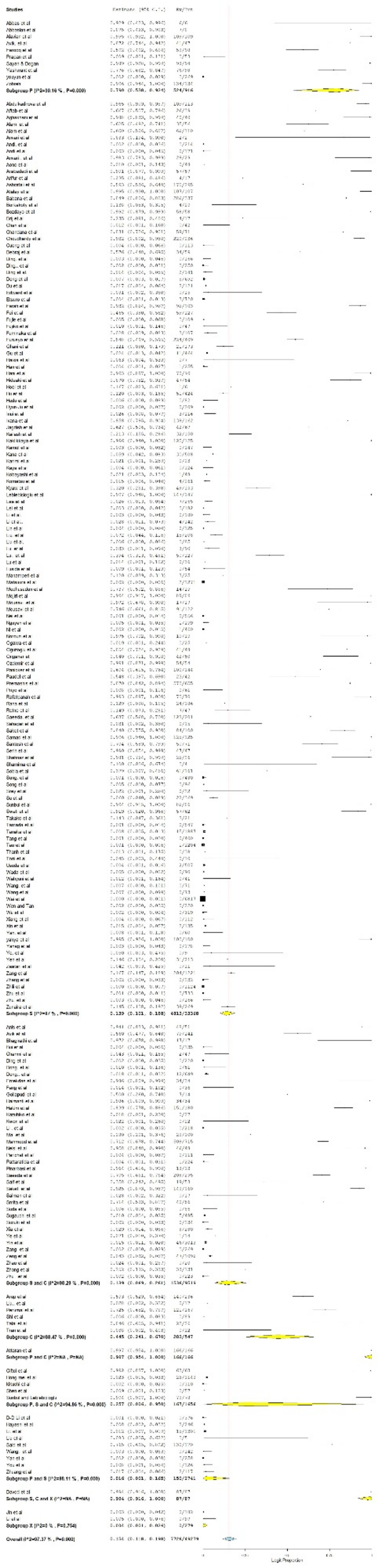

SUBGROUP FOREST PLOT E

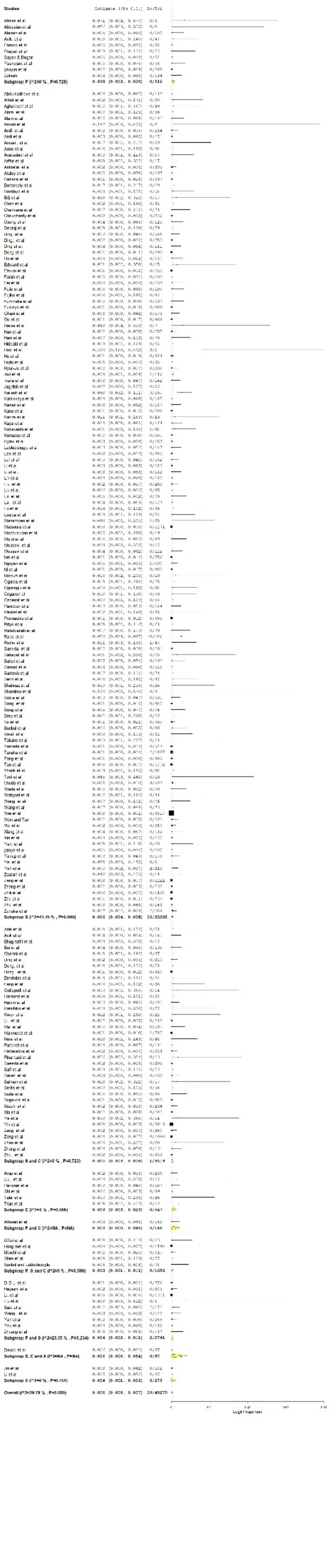

SUBGROUP FOREST PLOT F

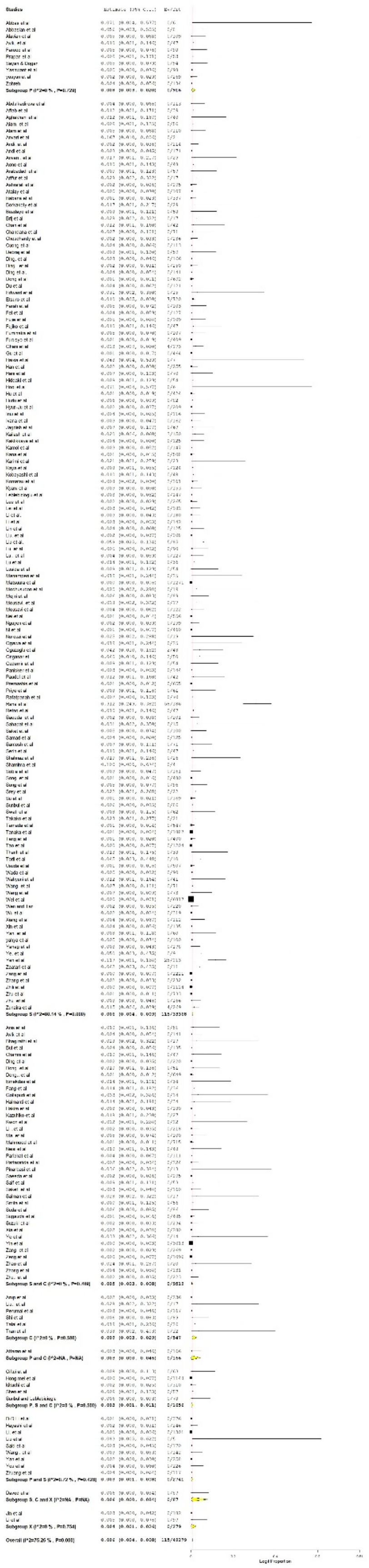

SUBGROUP FORET PLOT G

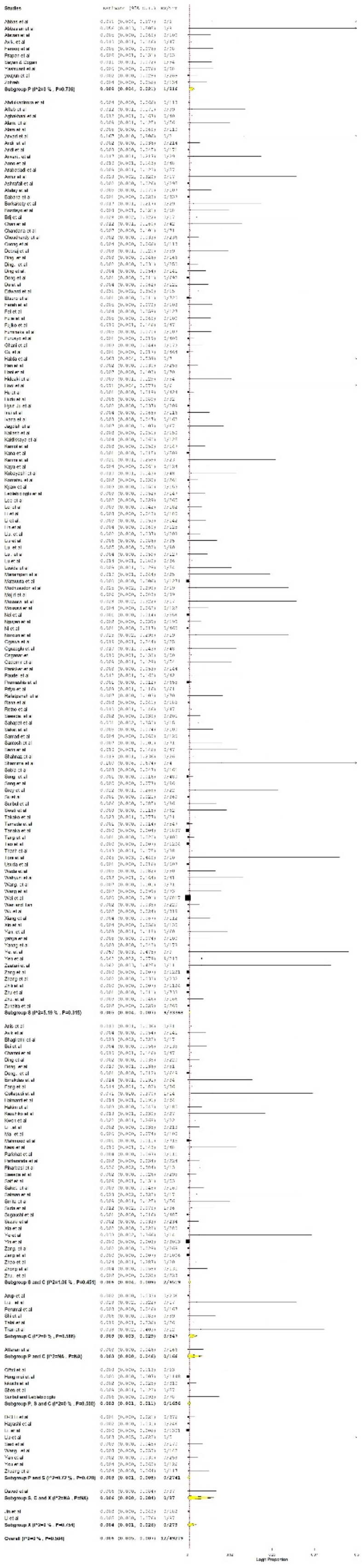

SUBGROUP FOREST PLOT H

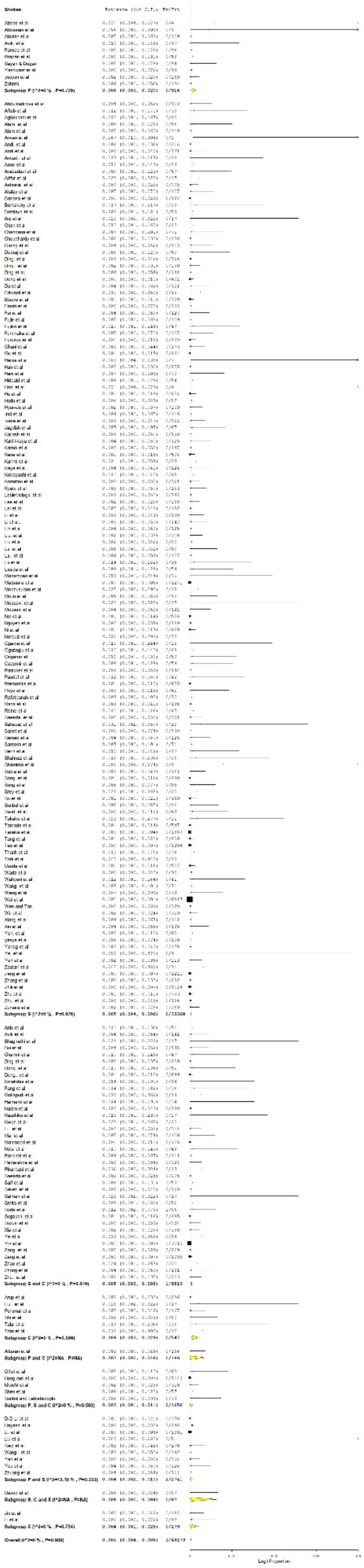

SUBGROUP FOREST PLOT I

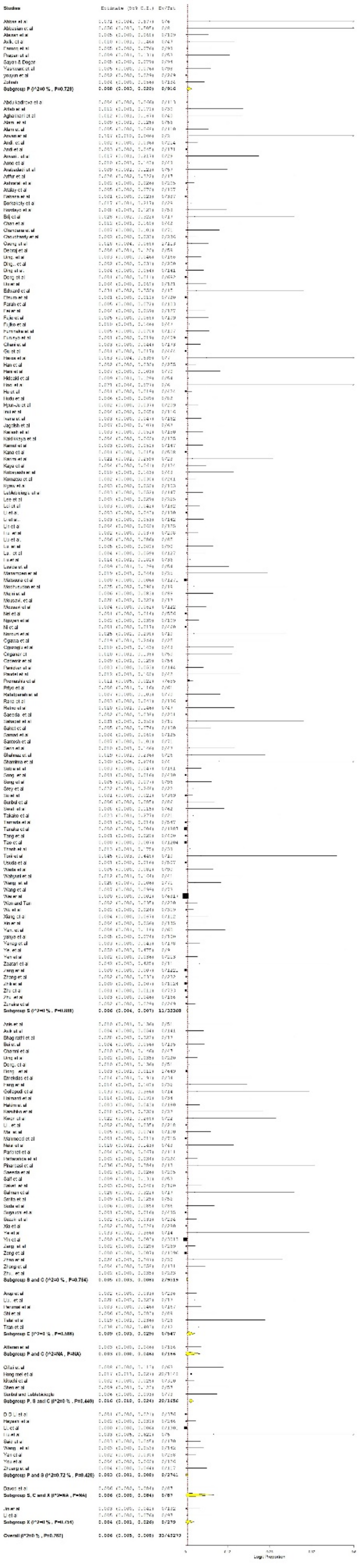

FOREST PLOT AB

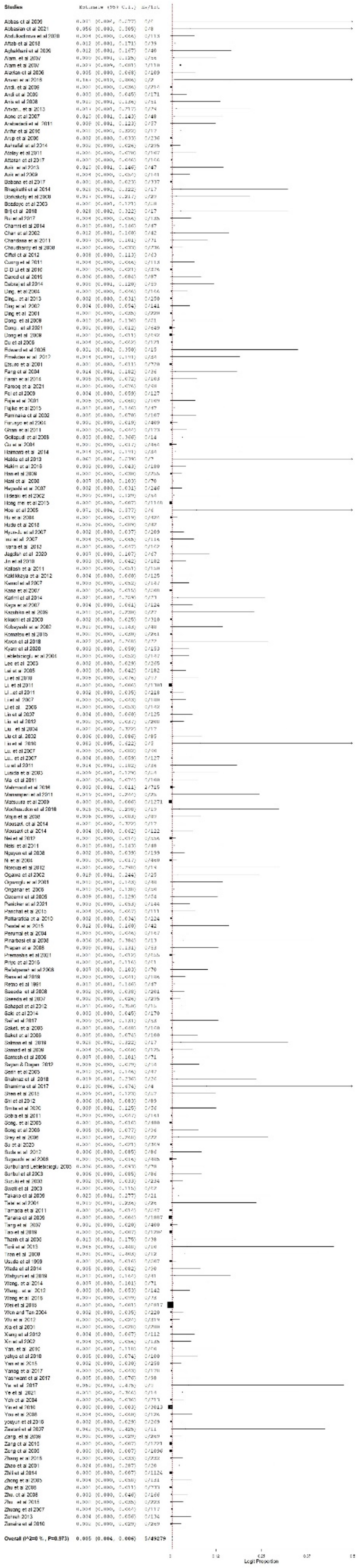

FOREST PLOT AC

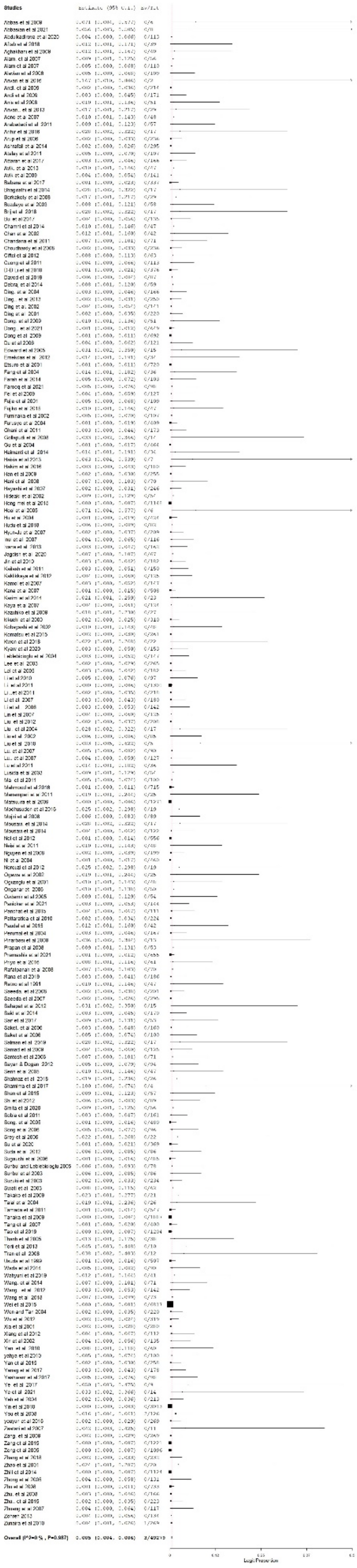

FOREST PLOT AD

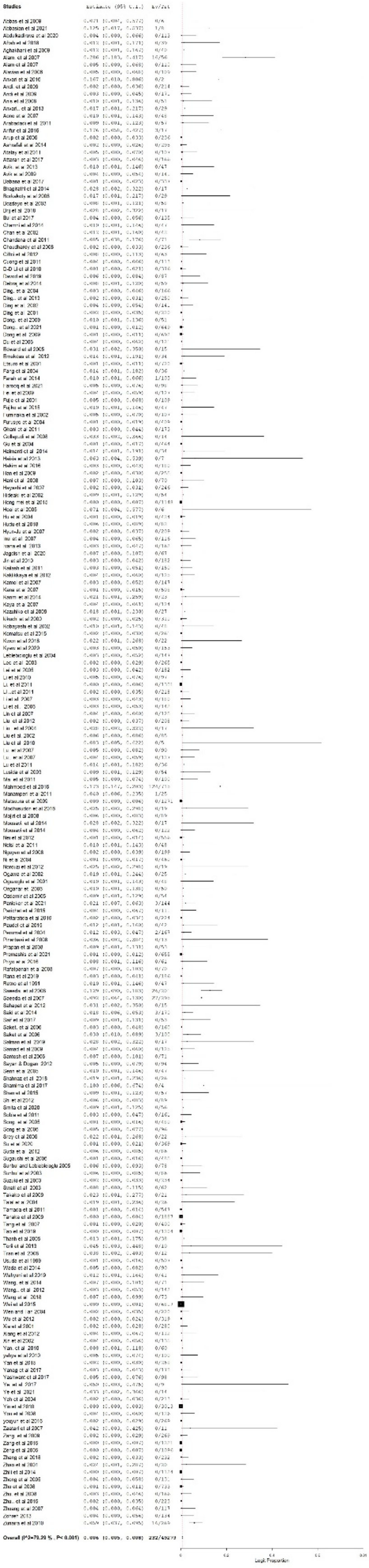

FOREST PLOT AE

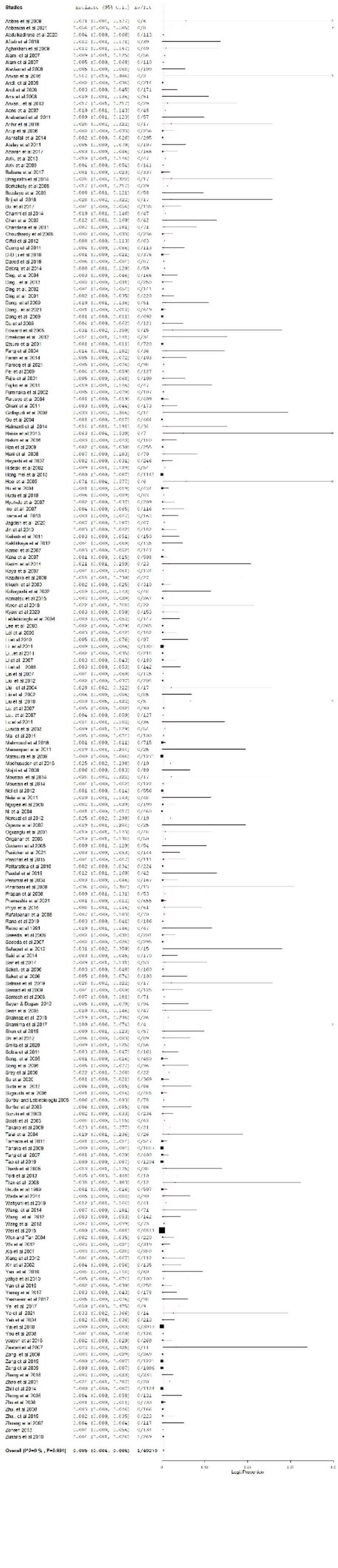

FOREST PLOT AF

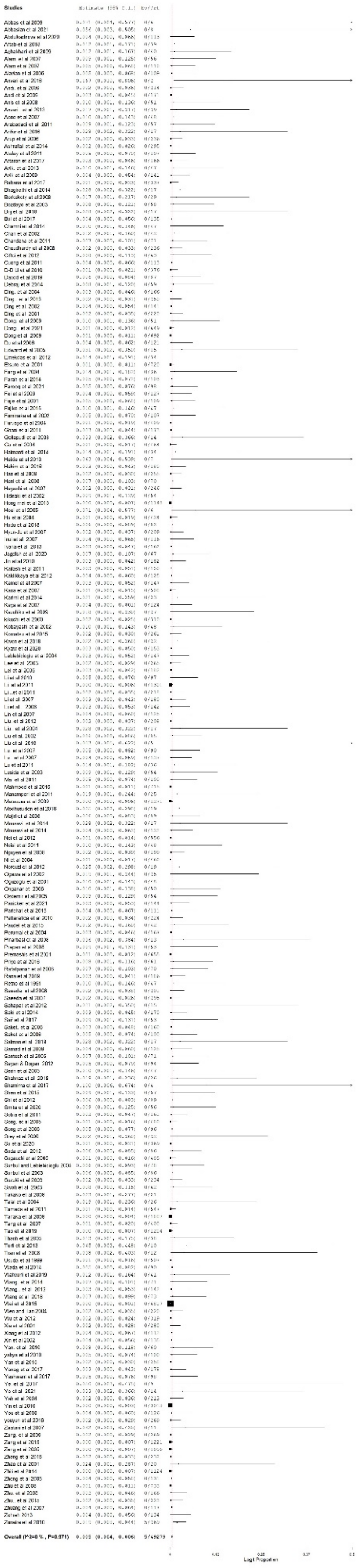

FORST PLOT BC

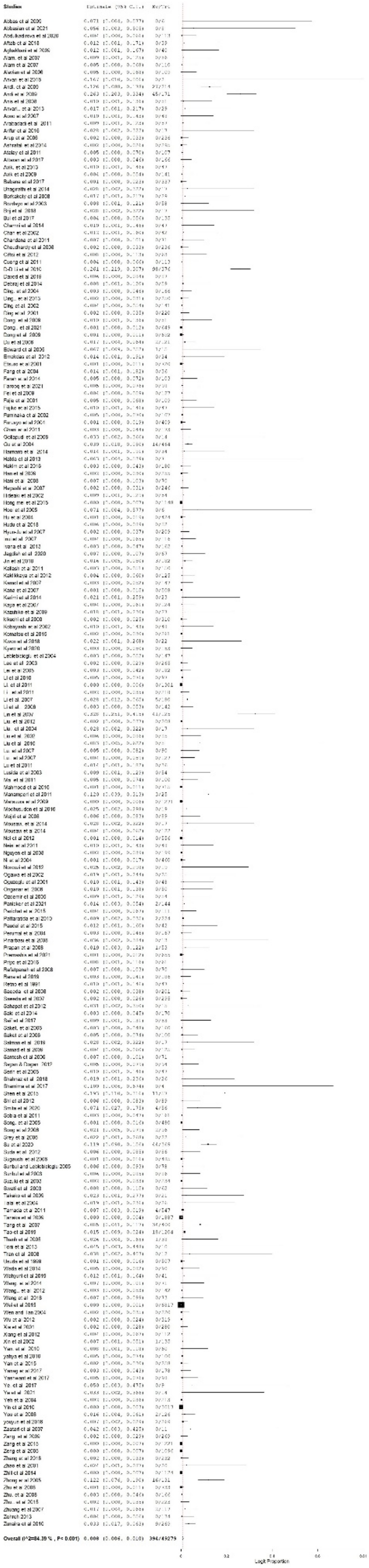

FOREST PLOT BD

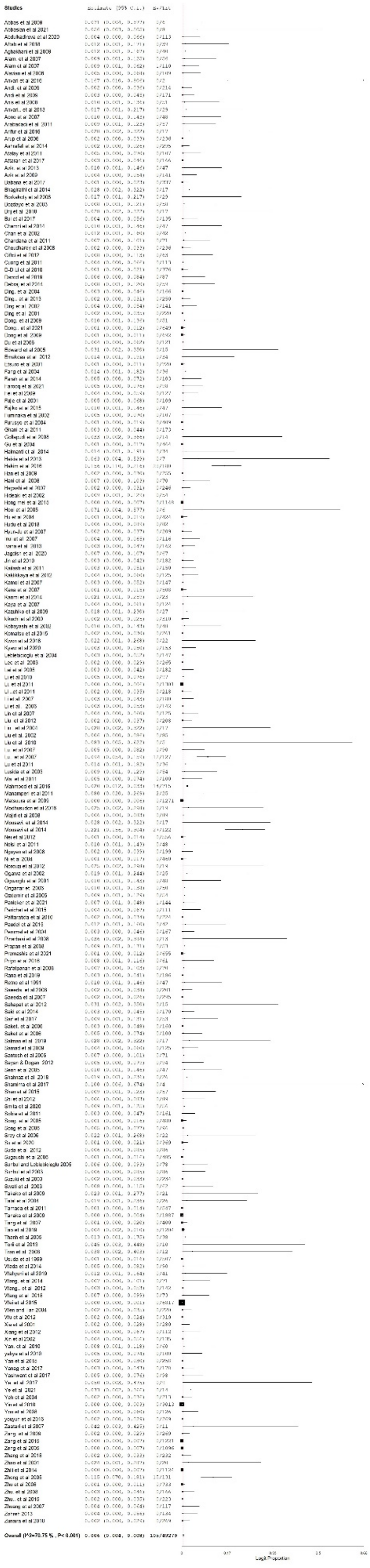

FOREST PLOT BE

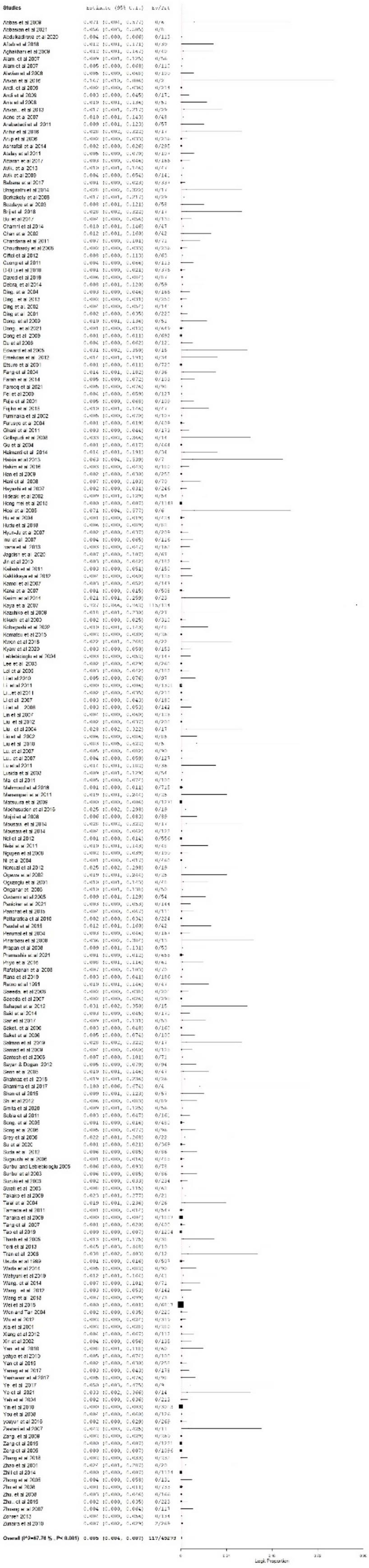

FOREST PLOT CD

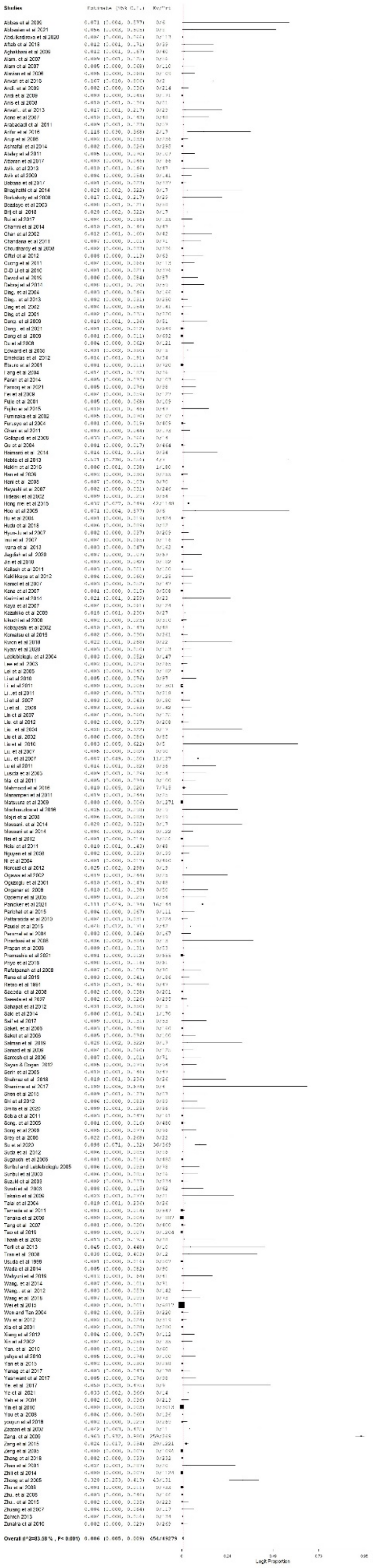

FOREST PLOT ED

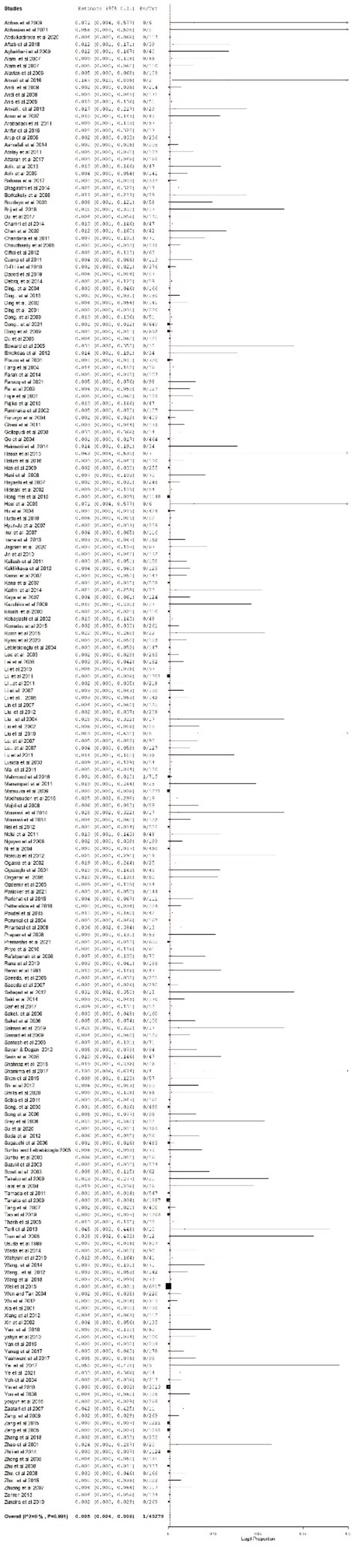

SUBGROUP FOREST PLOT A

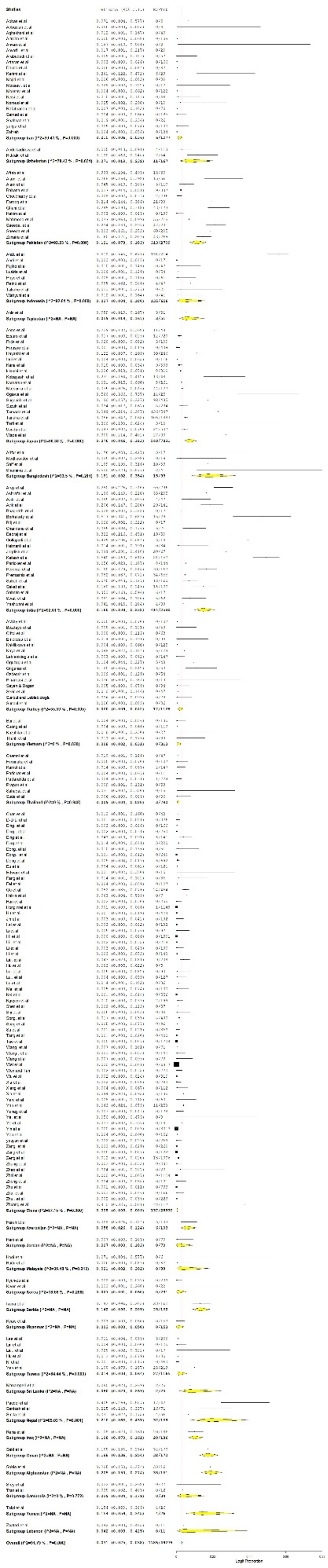

SUBGROUP FOREST PLOT B

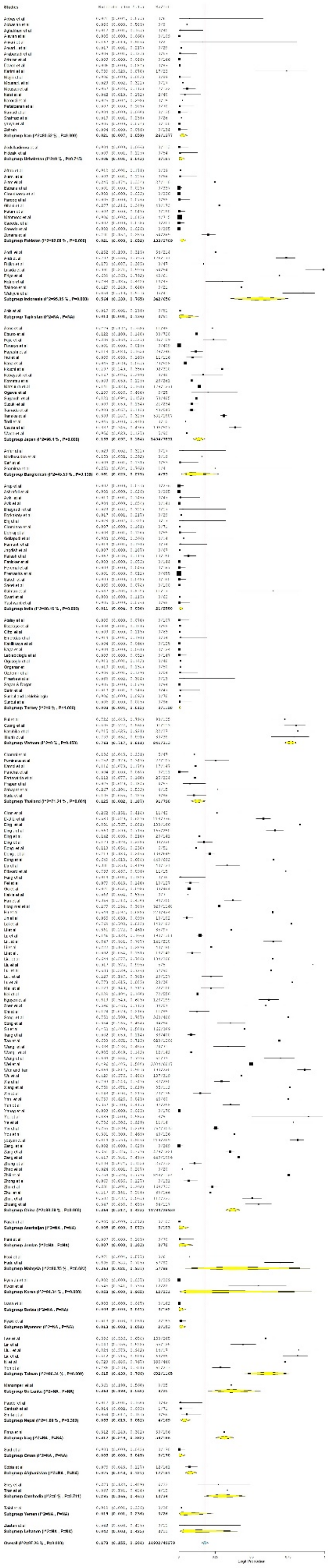

SUBGROUP FOREST PLOT C

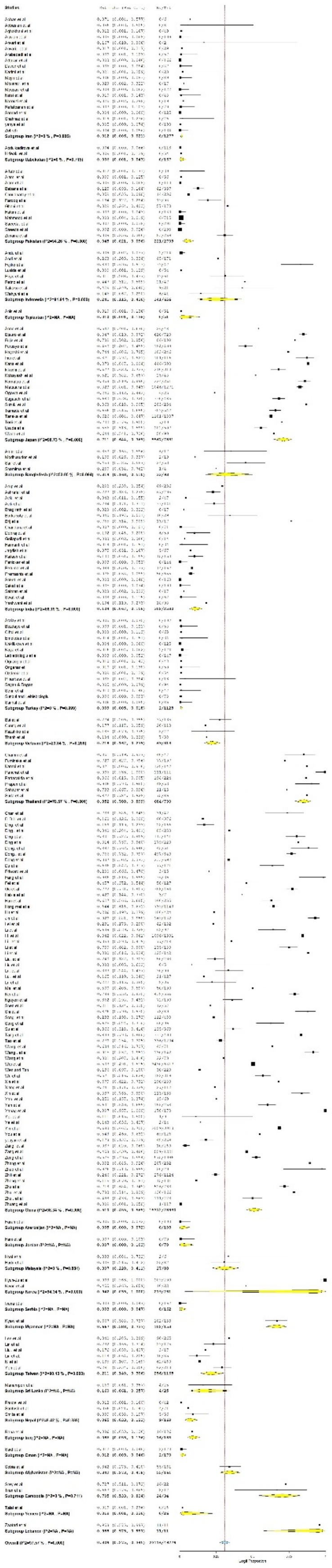

SUBGROUP FOREST PLOT D

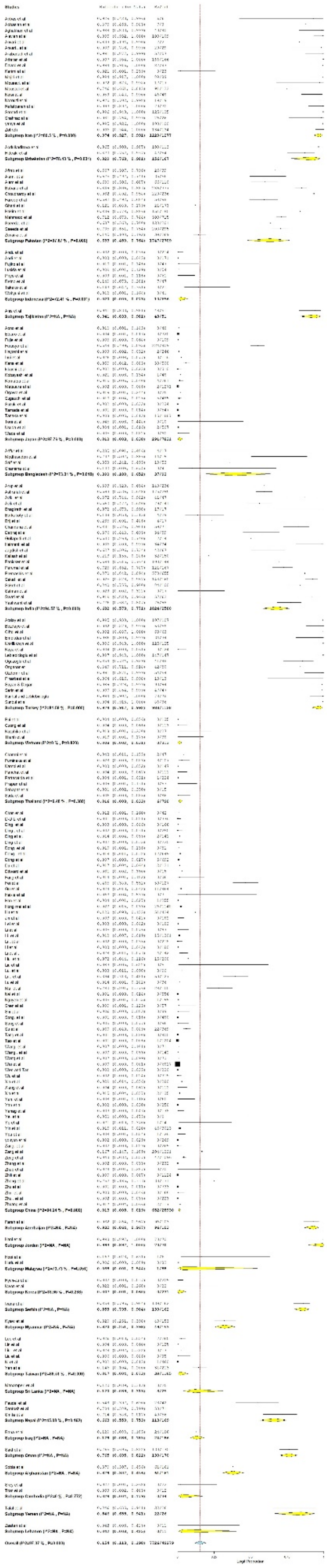

SUBGROUP FOREST PLOT E

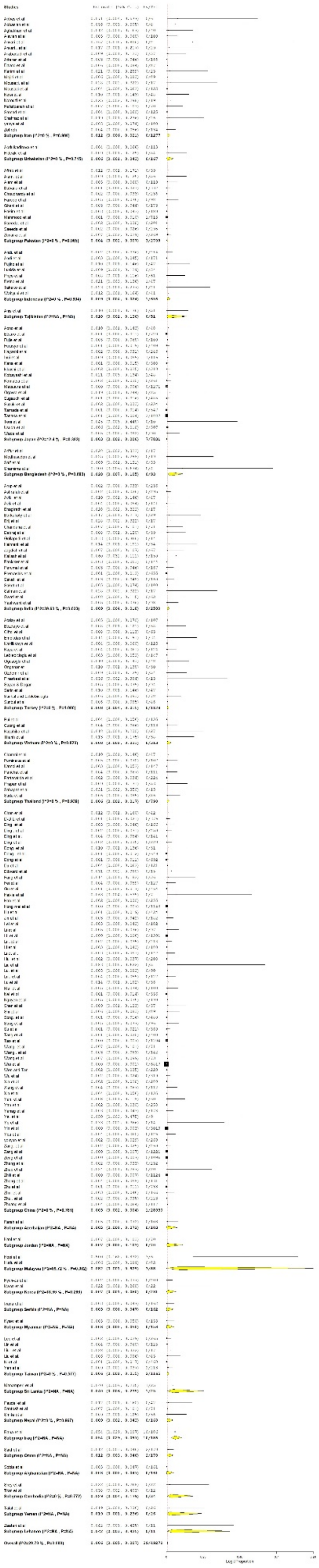

SUBGROUP FOREST PLOT F

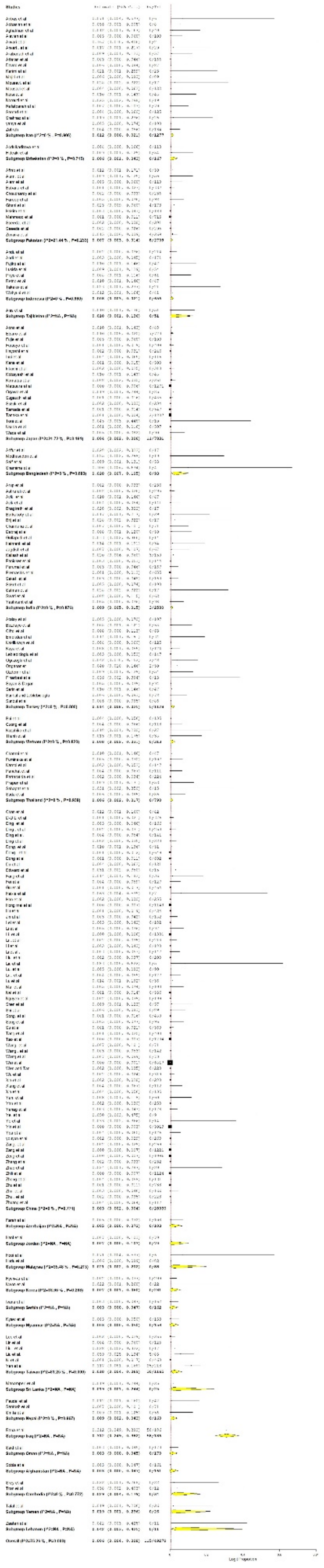

SUBGROUP FOREST PLOT G

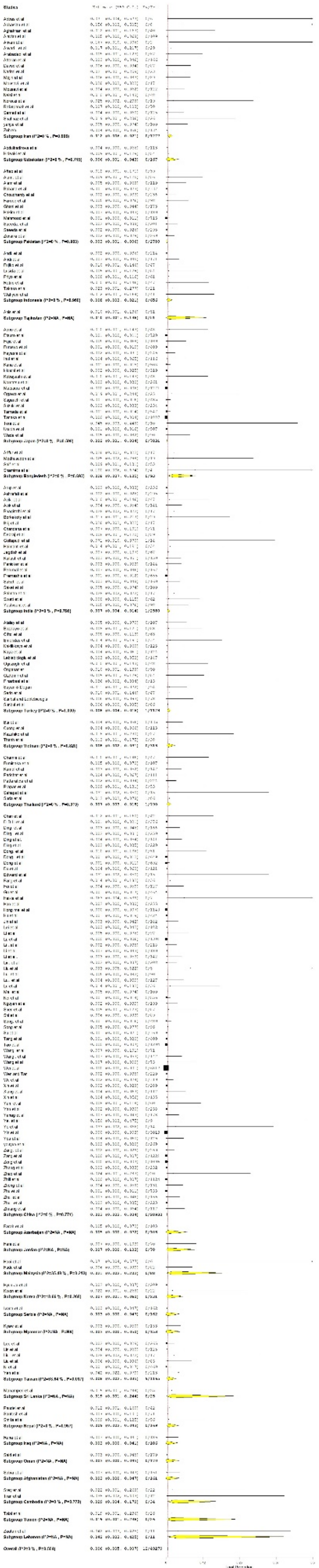

SUBGROUP FOREST PLOT H

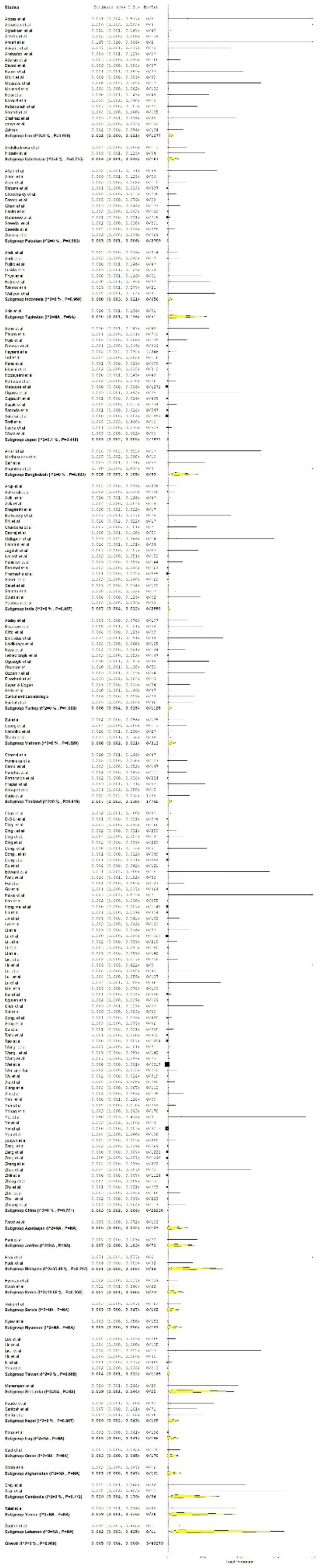

SUBGROUP FOREST PLOT I

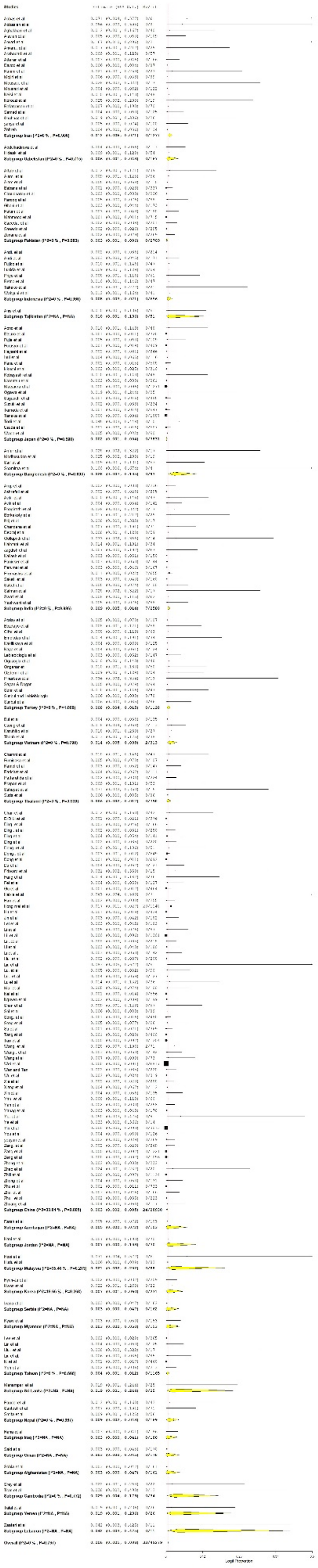

FOREST PLOT ABC

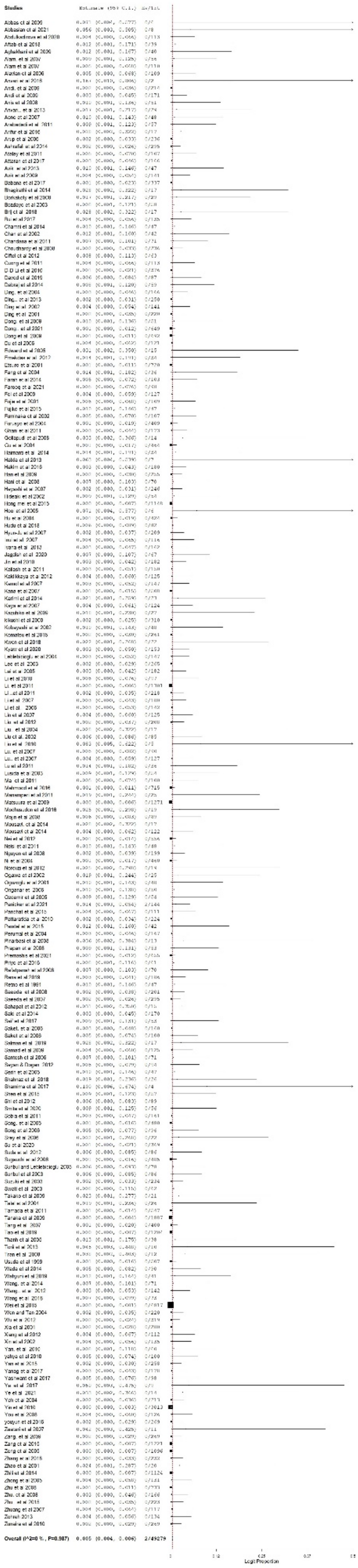

FOREST PLOT ABD

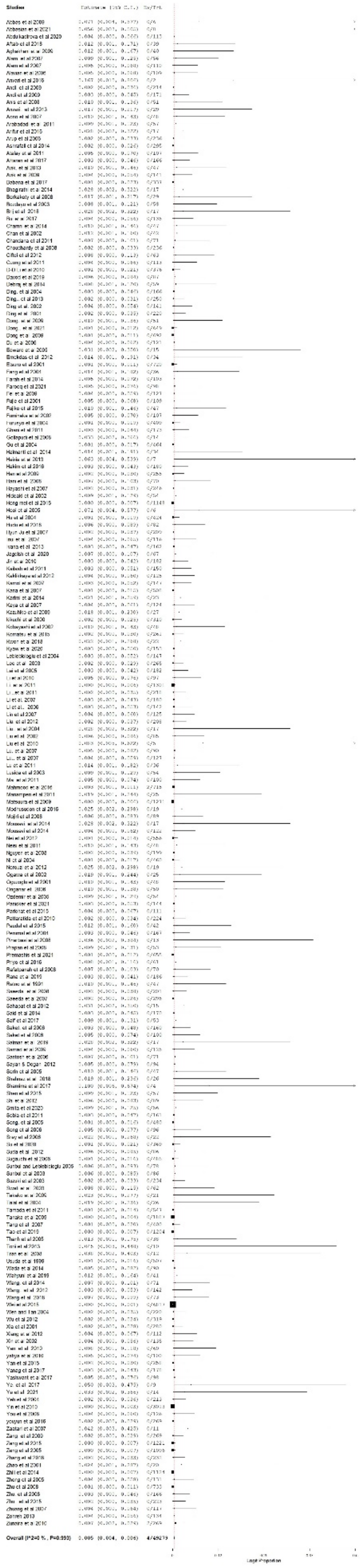

FOREST PLOT ACD

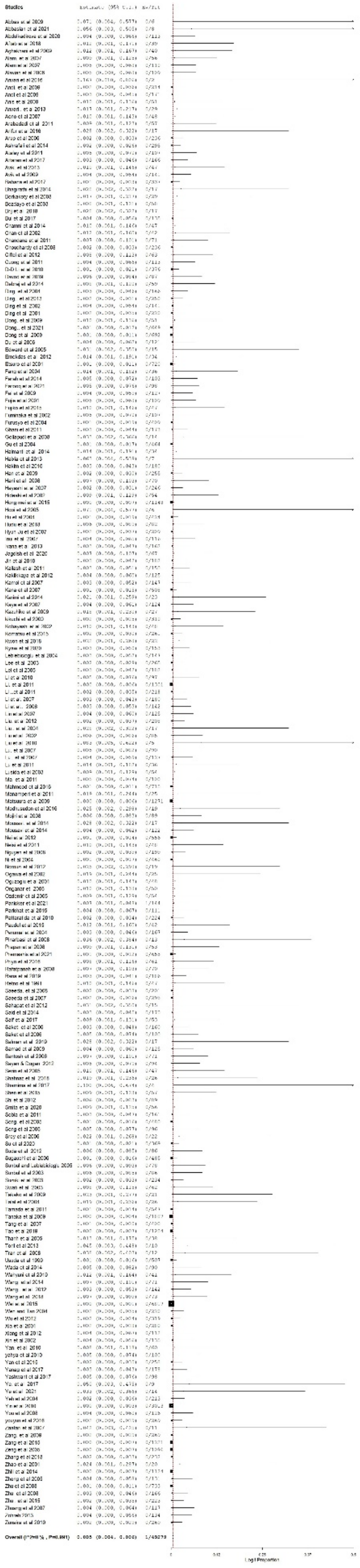

FOREST PLOT BCD

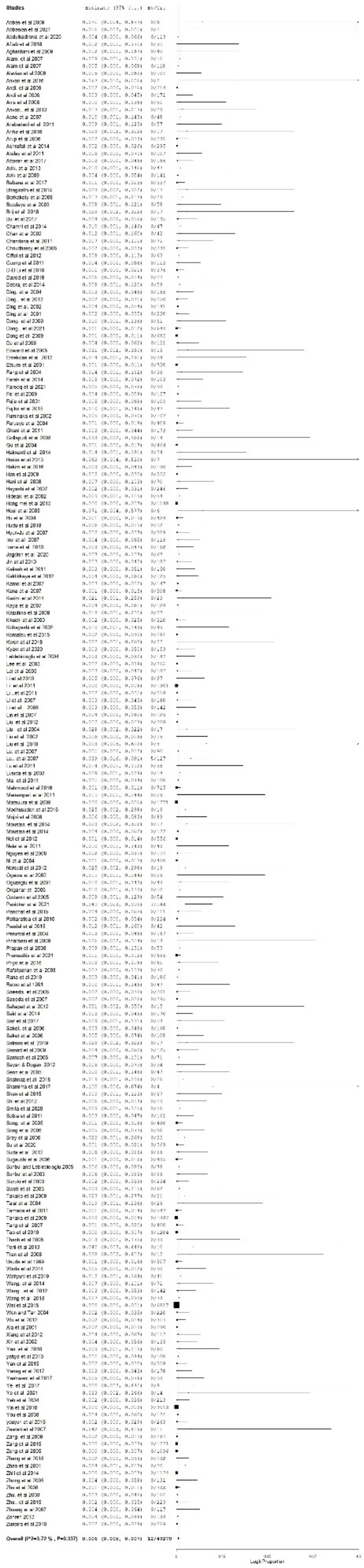

FOREST PLOT BCDE

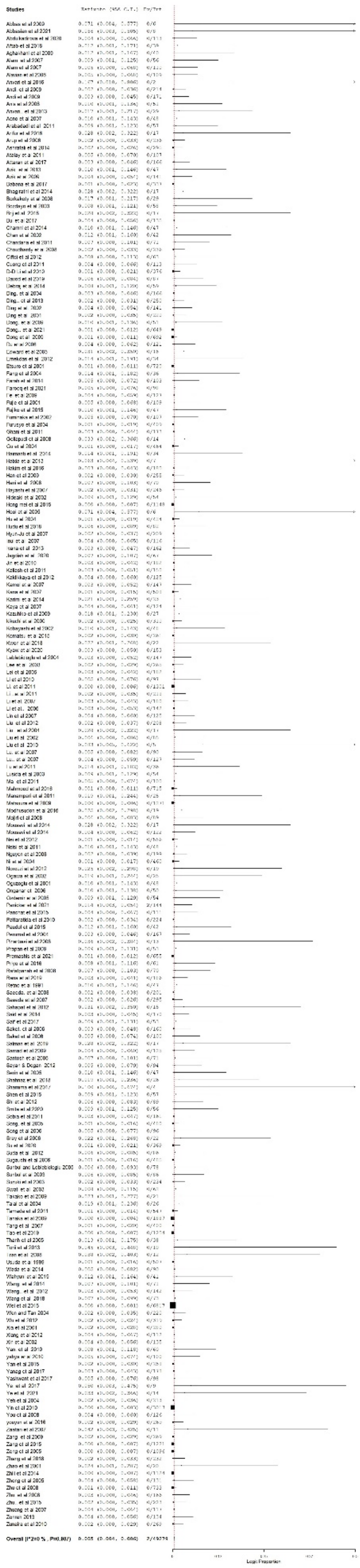

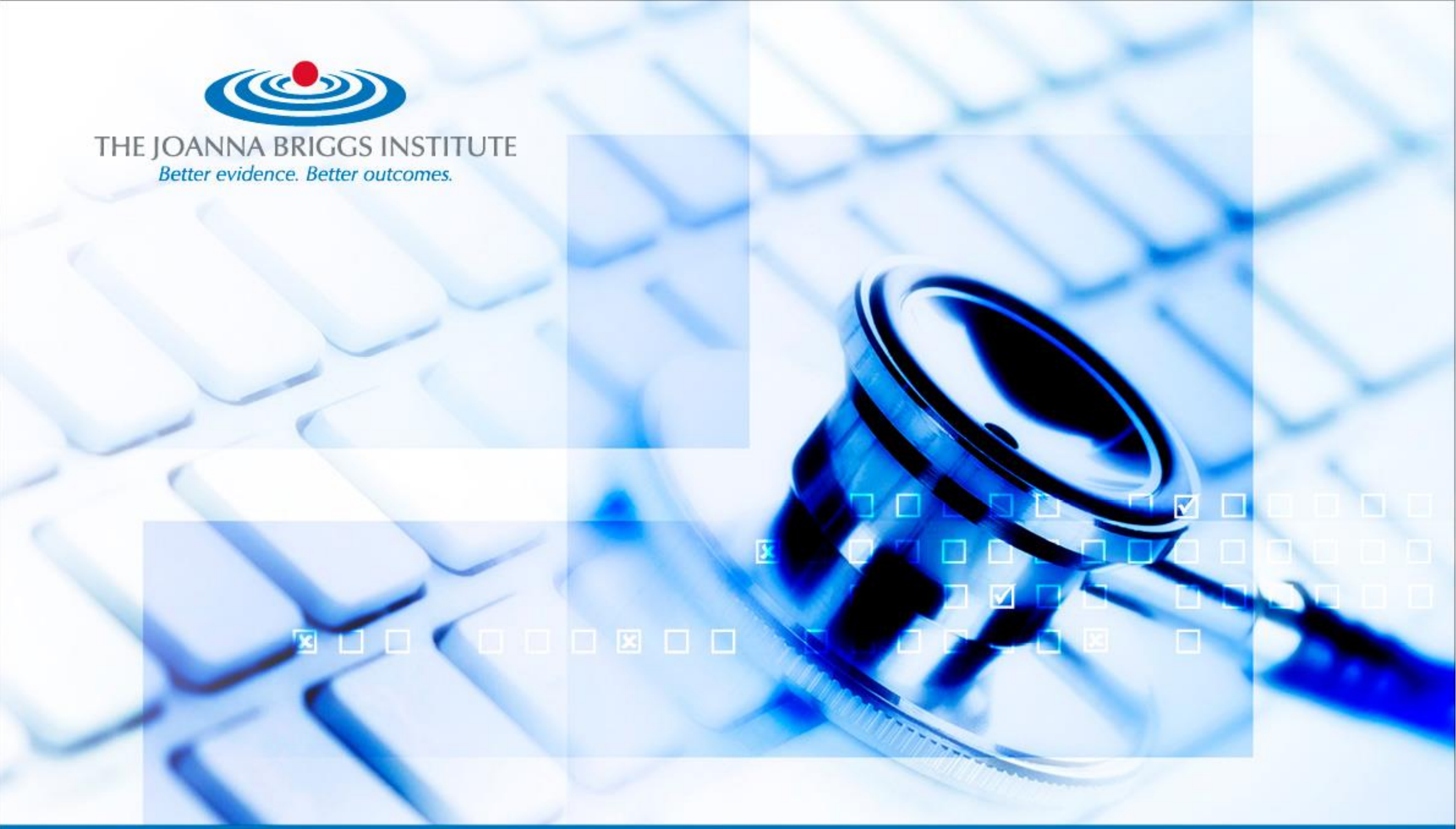

The Joanna Briggs Institute Critical Appraisal tools
for use in JBI Systematic Reviews

Checklist for Prevalence Studies

http://joannabriggs.org/research/critical-appraisal-tools.html

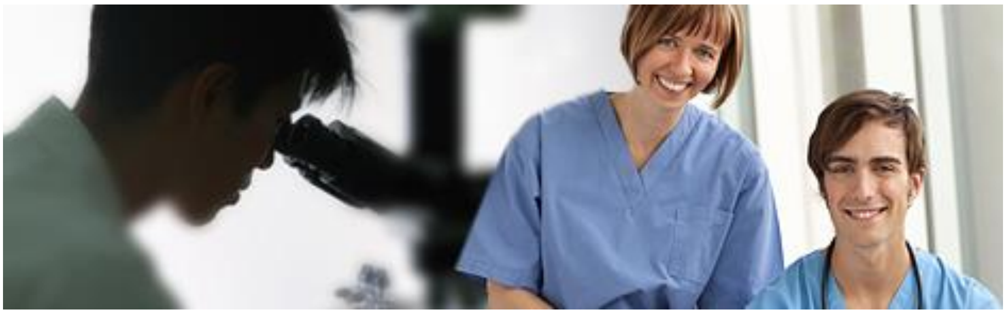

The Joanna Briggs Institute

Introduction

The Joanna Briggs Institute (JBI) is an international, membership based research and development organization within the Faculty of Health Sciences at the University of Adelaide. The Institute specializes in promoting and supporting evidence-based healthcare by providing access to resources for professionals in nursing, midwifery, medicine, and allied health. With over 80 collaborating centres and entities, servicing over 90 countries, the Institute is a recognized global leader in evidence-based healthcare.

JBI Systematic Reviews

The core of evidence synthesis is the systematic review of literature of a particular intervention, condition or issue. The systematic review is essentially an analysis of the available literature (that is, evidence) and a judgment of the effectiveness or otherwise of a practice, involving a series of complex steps. The JBI takes a particular view on what counts as evidence and the methods utilized to synthesize those different types of evidence. In line with this broader view of evidence, the Institute has developed theories, methodologies and rigorous processes for the critical appraisal and synthesis of these diverse forms of evidence in order to aid in clinical decision-making in health care. There now exists JBI guidance for conducting reviews of effectiveness research, qualitative research, prevalence/incidence, etiology/risk, economic evaluations, text/opinion, diagnostic test accuracy, mixed-methods, umbrella reviews and scoping reviews. Further information regarding JBI systematic reviews can be found in the JBI Reviewer's Manual on our website.

JBI Critical Appraisal Tools

All systematic reviews incorporate a process of critique or appraisal of the research evidence. The purpose of this appraisal is to assess the methodological quality of a study and to determine the extent to which a study has addressed the possibility of bias in its design, conduct and analysis. All papers selected for inclusion in the systematic review (that is – those that meet the inclusion criteria described in the protocol) need to be subjected to rigorous appraisal by two critical appraisers. The results of this appraisal can then be used to inform synthesis and interpretation of the results of the study. JBI Critical appraisal tools have been developed by the JBI and collaborators and approved by the JBI Scientific Committee following extensive peer review. Although designed for use in systematic reviews, JBI critical appraisal tools can also be used when creating Critically Appraised Topics (CAT), in journal clubs and as an educational tool.

© Joanna Briggs Institute 2017

THE JOANNA BRIGGS INSTITUTE

JBI Critical Appraisal Checklist for Studies Reporting Prevalence Data

Reviewer______________________________________Date___________________________________

Author ______________________________________Year_________Record Number____________

	Yes	No	Unclear	Not applicable
1. Was the sample frame appropriate to address the target population?	☐	☐	☐	☐
2. Were study participants sampled in an appropriate way?	☐	☐	☐	☐
3. Was the sample size adequate?	☐	☐	☐	☐
4. Were the study subjects and the setting described in detail?	☐	☐	☐	☐
5. Was the data analysis conducted with sufficient coverage of the identified sample?	☐	☐	☐	☐
6. Were valid methods used for the identification of the condition?	☐	☐	☐	☐
7. Was the condition measured in a standard, reliable way for all participants?	☐	☐	☐	☐
8. Was there appropriate statistical analysis?	☐	☐	☐	☐
9. Was the response rate adequate, and if not, was the low response rate managed appropriately?	☐	☐	☐	☐

Overall appraisal: Include ☐ Exclude ☐ Seek further info ☐

Comments (Including reason for exclusion)

© Joanna Briggs Institute 2017

THE JOANNA BRIGGS INSTITUTE

JBI Critical Appraisal Checklist for Studies Reporting Prevalence Data

How to cite: Munn Z, Moola S, Lisy K, Riitano D, Tufanaru C. Methodological guidance for systematic reviews of observational epidemiological studies reporting prevalence and incidence data. Int J Evid Based Healthc. 2015;13(3):147–153.

Answers: Yes, No, Unclear or Not/Applicable

1. Was the sample frame appropriate to address the target population?

This question relies upon knowledge of the broader characteristics of the population of interest and the geographical area. If the study is of women with breast cancer, knowledge of at least the characteristics, demographics and medical history is needed. The term "target population" should not be taken to infer every individual from everywhere or with similar disease or exposure characteristics. Instead, give consideration to specific population characteristics in the study, including age range, gender, morbidities, medications, and other potentially influential factors. For example, a sample frame may not be appropriate to address the target population if a certain group has been used (such as those working for one organisation, or one profession) and the results then inferred to the target population (i.e. working adults). A sample frame may be appropriate when it includes almost all the members of the target population (i.e. a census, or a complete list of participants or complete registry data).

2. Were study participants recruited in an appropriate way?

Studies may report random sampling from a population, and the methods section should report how sampling was performed. Random probabilistic sampling from a defined subset of the population (sample frame) should be employed in most cases, however, random probabilistic sampling is not needed when everyone in the sampling frame will be included/ analysed. For example, reporting on all the data from a good census is appropriate as a good census will identify everybody. When using cluster sampling, such as a random sample of villages within a region, the methods need to be clearly stated as the precision of the final prevalence estimate incorporates the clustering effect. Convenience samples, such as a street survey or interviewing lots of people at a public gatherings are not considered to provide a representative sample of the base population.

3. **Was the sample size adequate?**

The larger the sample, the narrower will be the confidence interval around the prevalence estimate, making the results more precise. An adequate sample size is important to ensure good precision of the final estimate. Ideally we are looking for evidence that the authors conducted a sample size calculation to determine an adequate sample size. This will estimate how many subjects are needed to produce a reliable estimate of the measure(s) of interest. For conditions with a low prevalence, a larger sample size is needed. Also consider sample sizes for subgroup (or characteristics) analyses, and whether these are appropriate. Sometimes, the study will be large enough (as in large national surveys) whereby a sample size calculation is not required. In these cases, sample size can be considered adequate.

When there is no sample size calculation and it is not a large national survey, the reviewers may consider conducting their own sample size analysis using the following formula: (Naing et al. 2006, Daniel 1999)

n= Z2P(1-P)

d2

Where:

n= sample size

Z = Z statistic for a level of confidence

P = Expected prevalence or proportion (in proportion of one; if 20%, P = 0.2)

d = precision (in proportion of one; if 5%, d=0.05)

Ref:

Naing L, Winn T, Rusli BN. Practical issues in calculating the sample size for prevalence studies Archives of Orofacial Sciences. 2006;1:9-14.

Daniel WW. Biostatistics: A Foundation for Analysis in the Health Sciences.

Edition. 7th ed. New York: John Wiley & Sons. 1999.

4. **Were the study subjects and setting described in detail?**

   Certain diseases or conditions vary in prevalence across different geographic regions and populations (e.g. Women vs. Men, sociodemographic variables between countries). The study sample should be described in sufficient detail so that other researchers can determine if it is comparable to the population of interest to them.

5. **Was data analysis conducted with sufficient coverage of the identified sample?**

   Coverage bias can occur when not all subgroups of the identified sample respond at the same rate. For instance, you may have a very high response rate overall for your study, but the response rate for a certain subgroup (i.e. older adults) may be quite low.

6. **Were valid methods used for the identification of the condition?**

   Here we are looking for measurement or classification bias. Many health problems are not easily diagnosed or defined and some measures may not be capable of including or excluding appropriate levels or stages of the health problem. If the outcomes were assessed based on existing definitions or diagnostic criteria, then the answer to this question is likely to be yes. If the outcomes were assessed using observer reported, or self-reported scales, the risk of over- or under-reporting is increased, and objectivity is compromised. Importantly, determine if the measurement tools used were validated instruments as this has a significant impact on outcome assessment validity.

7. **Was the condition measured in a standard, reliable way for all participants?**

   Considerable judgment is required to determine the presence of some health outcomes. Having established the validity of the outcome measurement instrument (see item 6 of this scale), it is important to establish how the measurement was conducted. Were those involved in collecting data trained or educated in the use of the instrument/s? If there was more than one data collector, were they similar in terms of level of education, clinical or research experience, or level of responsibility in the piece of research being appraised? When there was more than one observer or collector, was there comparison of results from across the observers? Was the condition measured in the same way for all participants?

© Joanna Briggs Institute 2017

8. Was there appropriate statistical analysis?

Importantly, the numerator and denominator should be clearly reported, and percentages should be given with confidence intervals. The methods section should be detailed enough for reviewers to identify the analytical technique used and how specific variables were measured. Additionally, it is also important to assess the appropriateness of the analytical strategy in terms of the assumptions associated with the approach as differing methods of analysis are based on differing assumptions about the data and how it will respond.

9. Was the response rate adequate, and if not, was the low response rate managed appropriately?

A large number of dropouts, refusals or "not founds" amongst selected subjects may diminish a study's validity, as can a low response rates for survey studies. The authors should clearly discuss the response rate and any reasons for non-response and compare persons in the study to those not in the study, particularly with regards to their socio-demographic characteristics. If reasons for non-response appear to be unrelated to the outcome measured and the characteristics of non-responders are comparable to those who do respond in the study (addressed in question 5, coverage bias), the researchers may be able to justify a more modest response rate.

© Joanna Briggs Institute 2017

name of auth	year of public	country	target region	method of ge	TOTAL SUBGE	A1
Saif et al	2017	Bangledesh	S and C	sequencing	45	9
Tran et al	2008	Cambodia	C	sequencing	12	0
Dong.. et al	2021	China	S and C	sequencing	636	0
Jin et al	2010	China	X	sequencing	179	0
Li et al	2010	China	X	sequencing	96	0
Li. et al	2011	China	P and S	sequencing	1290	0
Nei et al	2012	China	S	sequencing	470	0
Shen et al	2015	China	P, S and C	sequencing	37	0
Su et al	2020	China	S	sequencing	323	0
Wang. et al	2014	China	S	sequencing	45	0
Xiang et al	2012	China	S	sequencing	112	0
Yan et al	2015	China	P and S	multiplex PCF	247	0
Yin et al	2010	China	S and C	sequencing	2202	0
Gollapudi et a	2008	India	S and C	sequencing	10	7
Andi et al	2009	Indonesia	S	sequencing	171	0
Wahyuni et a	2019	Indonesia	S	sequencing	41	0
Davod et al	2019	Iran	S, C and X	sequencing	87	0
Shahnaz et al	2018	Iran	S	sequencing	26	0
Hayashi et al	2007	Japan	P and S	sequencing	242	0
Hyun-Ju et al	2007	Korea	S	sequencing	209	0
Hudu et al	2018	Malaysia	S	sequencing	75	0
Pattaratida et	2010	Thailand	S and C	sequencing	219	0
Kaklikkaya et	2012	Turkey	S	RFLP	125	0
Ozdemir et al	2005	Turkey	S	sequencing	54	0
Sayan & Doga	2012	Turkey	P	sequencing	93	0
Sunbul and Le	2005	Turkey	P, S and C	RFLP	78	0
Sunbul et al	2003	Turkey	S	RFLP	86	0
Bui et al	2017	Vietnam	S and C	sequencing	135	0
Cuong et al	2011	Vietnam	S	sequencing	112	0
					7457	16

A5	B1	B2	B3	B4	B5	B7
0	0	0	0	0	0	0
0	0	0	0	4	0	0
0	1	118	7	7	0	2
0	0	10	0	0	0	0
0	0	35	0	0	0	0
0	0	182	0	0	0	0
0	0	63	0	0	0	0
0	26	0	0	0	0	0
0	0	162	2	2	0	0
0	0	0	0	0	0	0
0	0	85	0	0	0	0
0	0	92	0	0	0	0
0	2	601	6	2	0	0
0	0	0	0	0	0	0
0	0	5	107	1	5	8
0	0	0	35	0	0	0
0	0	0	0	0	0	0
0	0	0	0	0	0	0
30	8	0	0	0	0	0
0	0	0	0	0	0	0
0	0	37	0	13	0	0
0	0	8	9	8	0	0
0	0	0	0	0	0	0
0	0	0	0	0	0	0
0	0	0	0	0	0	0
0	0	0	0	0	0	0
0	0	0	0	0	0	0
0	0	12	0	86	0	0
0	0	1	0	0	90	0
30	37	1411	166	123	95	10

B8	C1	C2	C3	C4	C5	C6
0	21	0	0	0	0	0
0	12	0	0	0	0	0
1	394	60	0	0	17	0
0	0	169	0	0	0	0
0	4	57	0	0	0	0
0	0	1096	0	0	0	0
0	0	407	0	0	0	0
0	0	0	0	0	11	0
0	27	93	3	6	4	2
0	10	28	0	0	3	0
0	0	27	0	0	0	0
0	30	125	0	0	0	0
0	236	1277	4	2	15	0
0	0	0	0	0	0	0
0	37	1	7	0	0	0
0	6	0	0	0	0	0
0	0	0	0	0	0	0
0	0	0	0	0	0	0
21	0	176	0	0	7	0
0	0	209	0	0	0	0
0	0	22	0	3	0	0
0	192	0	0	0	2	0
0	0	0	0	0	0	0
0	0	0	0	0	0	0
0	0	0	0	0	0	0
0	0	0	0	0	0	0
0	0	0	0	0	0	0
0	37	0	0	0	0	0
0	20	0	0	0	0	0
22	1026	3747	14	11	59	2

C7	C8	C9	C10	C17	D1	D2
0	0	0	0	0	0	15
0	0	0	0	0	0	0
0	24		1	1	0	0
0	0	0	0	0	0	0
0	0	0	0	0	0	0
0	0	0	0	0	12	0
0	0	0	0	0	0	0
0	0	0	0	0	0	0
0	0	0	0	0	19	0
4	0	0	0	0	0	0
0	0	0	0	0	0	0
0	0	0	0	0	0	0
0	0	31	0	0	21	5
0	0	0	0	0	1	2
0	0	0	0	0	0	0
0	0	0	0	0	0	0
0	0	0	0	0	87	0
0	0	0	0	0	24	2
0	0	0	0	0	0	0
0	0	0	0	0	0	0
0	0	0	0	0	0	0
0	0	0	0	0	0	0
0	0	0	0	0	2	122
0	0	0	0	0	7	44
0	0	0	0	0	76	9
0	0	0	0	0	3	67
0	0	0	0	0	4	74
0	0	0	0	0	0	0
0	0	0	0	0	0	0
4	24	31	1	1	256	340

D3	D4	D5	D6	D/Ddel
0	0	0	0	0
0	0	0	0	0
0	0	0	0	0
0	0	0	0	0
0	0	0	0	0
0	0	0	0	0
0	0	0	0	0
0	0	0	0	0
2	1	0	0	0
0	0	0	0	0
0	0	0	0	0
0	0	0	0	0
0	0	0	0	0
0	0	2	0	0
0	0	0	0	0
0	0	0	0	0
0	0	0	0	0
0	0	0	0	0
0	0	0	0	0
0	0	0	0	0
0	0	0	0	0
0	0	0	0	0
0	0	0	0	1
0	0	0	3	0
8	0	0	0	0
1	0	0	0	7
2	0	0	0	0
0	0	0	0	0
0	0	0	0	0
13	1	2	3	8
